# RADX protects against intestinal inflammation by restraining IFI16-mediated innate immunity

**DOI:** 10.1038/s44321-026-00494-6

**Published:** 2026-07-24

**Authors:** Huifang Xian, Wanming Huang, Zhanghua Chen, Lili Li, Liya Xiong, Jianheng Zhu, Haolan Jin, Xiaotian Li, Lizhi Song, Peiyu Chen, Lu Ren, Wei Wang, Rongli Fang, Xixi Chen, Wen Lei, Dengfeng Zhang, Weiliang Liao, Yuxin Huang, Andrew M Lew, Jun Cui, Sitang Gong, Jun Huang, Jaime S Rosa Duque, Min Zhi, Lanlan Geng, Wenhao Zhou, Yuxia Zhang

**Affiliations:** 1 Department of Gastroenterology, Guangzhou Women and Children’s Medical Center, Guangzhou Medical University, Guangzhou, China; 2 Chongqing International Institute for Immunology, Chongqing, China; 3 MOE Laboratory of Biosystems Homeostasis and Protection and Innovation Center for Cell Signaling Network, Life Sciences Institute, Zhejiang University, Hangzhou, China; 4 Guangdong Provincial Key Laboratory of Colorectal and Pelvic Floor Diseases, the Six Affiliated Hospital, Sun Yat-sen University, Guangdong, China; 5 Pediatric Immunity and Healthcare (Guangzhou) Biotechnology Co., Ltd, Guangzhou, China; 6 Walter and Eliza Hall Institute of Medical Research and Department of Medical Biology, University of Melbourne, Melbourne, VIC Australia; 7 Guangdong Province Key Laboratory of Pharmaceutical Functional Genes, MOE Key Laboratory of Gene Function and Regulation, State Key Laboratory of Biocontrol, School of Life Sciences, Sun Yat-sen University, Guangzhou, China; 8 Department of Pediatrics and Adolescent Medicine, The University of Hong Kong, Hong Kong SAR, China; 9 The Third Affiliated Hospital of Zhengzhou University, Zhengzhou, China

**Keywords:** Digestive System, DNA Replication, Recombination & Repair, Immunology

## Abstract

Genomic instability is increased in patients with inflammatory bowel disease (IBD), yet whether it contributes directly to disease pathogenesis remains unclear. Here, we identify RADX, a structural antagonist to RAD51 and a key regulator of replication fork stability, as a critical suppressor of intestinal inflammation by limiting innate immune sensing of replication-associated DNA damage. RADX deficiency exacerbates experimental colitis, with macrophages serving as the principal mediators of this phenotype. Mechanistically, RADX competes with the DNA sensor IFI16 for binding to single-stranded DNA (ssDNA). Loss of RADX promotes ssDNA accumulation, triggering IFI16-dependent activation of NF-κB signaling and inflammasome assembly, thereby driving intestinal inflammation. Consistent with these findings, two RADX variants identified in patients with IBD associate with reduced RADX protein expression, increased DNA damage signaling, and elevated IL-1β levels. Pharmacological inhibition of RAD51 with RI-1 alleviated colitis in both wild-type and *Radx*-deficient mice. Together, these findings establish a mechanistic link between genome instability and intestinal inflammation, identify a RADX-IFI16 checkpoint that restrains pathogenic innate immune activation, and nominate modulation of replication stress as a therapeutic strategy for IBD.

## Synopsis

Genomic instability is observed in IBD, yet its pathogenic mechanism is unclear. Here, RADX, replication fork protector, is shown to suppress colitis by limiting ssDNA accumulation and IFI16-dependent inflammation, revealing a new therapeutic vulnerability.

- Colitis exacerbation caused by RADX deficiency was shown to be predominantly driven by macrophages in acute murine models.
- ssDNA was found to be competitively bound by RADX and IFI16, and loss of RADX unleashed NF-κB and inflammation activation.
- Two loss-of-function RADX variants were identified in IBD patients and were associated with increased DNA damage and elevated IL-1β.
- Genomic instability was suggested as a significant risk factor for IBD by cohort analysis.
- Colitis phenotypes were alleviated by the RAD51 inhibitor RI-1 in both wild-type and Radx-deficient mice.

## Abstract (web summary)

Genomic instability is observed in IBD, yet its pathogenic mechanism is unclear. Here, RADX, replication fork protector, is shown to suppress colitis by limiting ssDNA accumulation and IFI16-dependent inflammation, revealing a new therapeutic vulnerability.

## Graphical abstract

**Figure:**
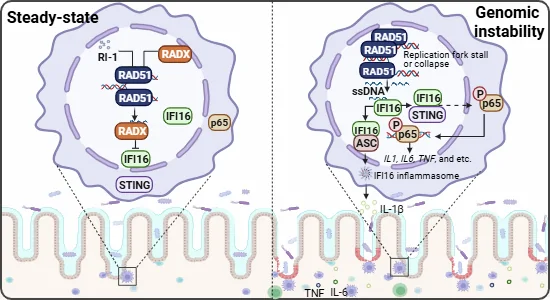

**Figure:**
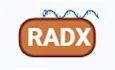

The paper explained **Problem**

Inflammatory bowel disease (IBD) is a chronic, relapsing intestinal disorder with rising incidence worldwide, including in children. Although genomic instability is elevated in IBD patients, the specific genes and pathways that connect DNA damage to intestinal inflammation have remained unclear. Identifying these links is essential for developing precise diagnostic and targeted therapies.

**Results**

This study reveals that RADX, a protein that protects stalled replication forks, is crucial for intestinal health. RADX maintains genomic stability and prevents excessive DNA damage, while also limiting IFI16-mediated NF-κB and inflammasome activation in macrophages. Mice lacking RADX develop more severe colitis, and bone marrow transplantation experiments show that exacerbation is largely driven by macrophages. In patients with IBD, two rare RADX variants were identified; these variants are associated with lower RADX protein levels, elevated DNA damage signaling, and increased IL-1β production. Importantly, treating mice with RI-1, a drug that inhibits RAD51, reduced DNA damage and alleviated colitis in both normal and RADX-deficient animals.

**Impact**

These findings establish a mechanistic link between genome maintenance and intestinal inflammation, providing a rationale for exploring genomic stability pathways as a therapeutic entry point in IBD.

## Introduction

Inflammatory bowel disease (IBD) is a chronic and relapsing inflammatory disorder of the intestine, primarily comprises Crohn’s disease (CD) and ulcerative colitis (UC) (Friedrich et al, 2019). Globally, more than 6 million people are affected by IBD, with approximately one-quarter diagnosed during childhood or adolescence. The incidence of pediatric-onset IBD (PIBD, defined as diagnosis before the age of 16) has been rising worldwide (Ng et al, 2018). This increase is largely attributed to modern environmental exposures, which are thought to exert profound adverse effects on genetically susceptible individuals (Huang et al, 2019; Jostins et al, 2012; Okou and Kugathasan, 2014). Supporting the role of genetic factors, elevated DNA damage and increased somatic mutation burdens have been observed in peripheral blood mononuclear cells (PBMCs) (Pereira et al, 2016) as well as in colonic biopsies (Kakiuchi et al, 2020; Nanki et al, 2020; Olafsson et al, 2020) of IBD patients. However, the specific genes that promote genomic instability to confer PIBD risk remain to be elucidated.

DNA double-strand breaks (DSBs) and solitary DNA ends, arising from events such as chromosome breakage or replication stress, pose a severe threat to genomic integrity (Scully et al, 2019). These lesions can trigger chromosomal rearrangements and disrupt gene integrity, thereby compromising genomic stability (Scully et al, 2019). As a hallmark of various human diseases, genomic instability is a well-established driver of inflammation, cancer, and age-related diseases (Li and Chen, 2018). DNA damage induced by irradiation or ATM deficiency can lead to the accumulation of cytoplasmic DNA, activating the STING-dependent type I interferon pathway (Hartlova et al, 2015). Furthermore, etoposide-induced DNA damage has been shown to activate IFI16/STING-dependent NF-κB pathway (Dunphy et al, 2018).

RAD51, a key recombinase, forms filaments on single-stranded DNA (ssDNA) at resected DSBs ends and catalyzes strand exchange to initiate homology-directed repair (HDR) (Kowalczykowski, 2015; Symington, 2014). It also facilitates the reversal of stalled replication forks and protects them from nuclease-catalyzed degradation of nascent DNA strands (Hashimoto et al, 2010; Zellweger et al, 2015). However, the excessive RAD51 activity causes fork collapse (Dungrawala et al, 2017). Dungrawala and colleagues identified RADX as a replication fork-associated factor that antagonizes RAD51 accumulation, thereby preserving fork stability and genomic integrity (Dungrawala et al, 2017). Given that RADX deficiency elevates γH2AX (DNA damage marker) levels both in the absence and the presence of hydroxyurea (Dungrawala et al, 2017), we wonder if RADX is involved in IBD pathogenesis.

In this study, we found that RADX protects against intestinal inflammation by maintaining genomic stability, limiting ssDNA accumulation, and competing with IFI16 for DNA binding, thereby attenuating NF-κB and inflammasome activation in macrophages. Pharmacological inhibition of RAD51 with RI-1 alleviated DNA damage, inflammation, and colitis phenotypes. Furthermore, cohort data reveal that genomic instability represents a risk factor for IBD, rather than an isolated observation. Although IBD pathogenesis involves multiple genetic, immunological, and environmental factors, this study identifies the RADX-IFI16 axis as a contributor to intestinal inflammation. Our findings provide new insights for therapeutic interventions by targeting the interplay between genomic instability and inflammation.

## Results

### *Radx* deficiency aggravates colitis in mouse models

**Figure EV1:**
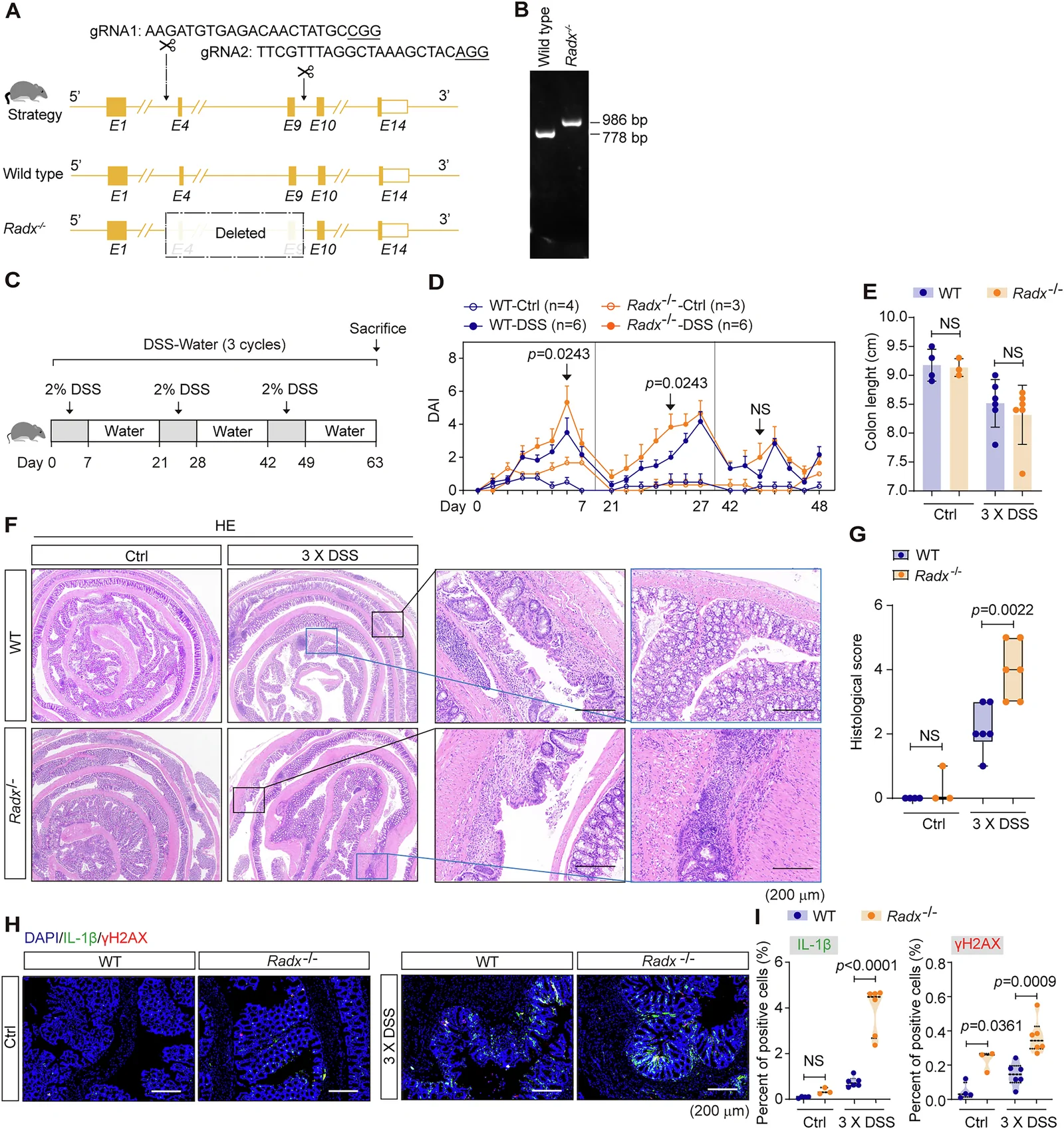
*Radx*^−/−^ mice show an exacerbated colitis phenotype in DSS-induced chronic colitis model. (**A**) CRISPR/Cas9 strategy was used to generate the *Radx*^−/−^ mouse line on C57BL/6 background. (**B**) Gel electrophoresis showing representative genotyping data for WT and *Radx*^−/−^ mice. (**C**) Schematic of DSS-induced chronic colitis model. (**D**,** E**) Disease activity index (DAI) (**D**) and colon lengths (**E**) of WT or *Radx*^−/−^ mice in DSS-induced acute colitis model. *n* = 4 in WT-Ctrl group, *n* = 6 in WT-DSS group, *n* = 3 in *Radx*^−/−^-Ctrl group, and *n* = 6 in *Radx*^−/−^-DSS group. Each data point represents one biologically independent individual. (**F**–**I**) Representative images of hematoxylin-eosin (H&E) staining (**F**), histological scores (**G**), immunofluorescence analysis (**H**), and quantification of immunofluorescence staining (**I**) of distal colon segments from WT or *Radx*^−/−^ mice in DSS-induced chronic colitis model. Each data point represents one biologically independent individual. Data were presented as mean ± SEM and analyzed using one-way (**E**, **G**, **I**) or two-way (**D**) ANOVA followed by Tukey–Kramer multiple-comparisons test. In box plot, the centre line represents the median, the box boundaries represent the 25th and 75th percentiles, the whiskers extend from the minimum to the maximum values, and all individual data points are shown. The exact *P* values are indicated in the figures. Source data are available online for this figure.

To investigate the role of RADX in colitis, we generated a *Radx*-deficiency mouse line (*Radx*^-/-^) on a C57BL/6 background using CRISPR-Cas9 (Fig. EV1A and B). We employed two established models: DSS-induced acute colitis, which resembles human ulcerative colitis (UC) (Okayasu et al, 1990), and 2,3,6-trinitrobenzene sulfonic acid (TNBS)/ethanol-induced acute colitis, which shares features with human Crohn’s disease (CD) (Antoniou et al, 2016). Despite their differences, both UC and CD are characterized by hyperinflammation (Huang et al, 2019; Mitsialis et al, 2020).

**Figure 1:**
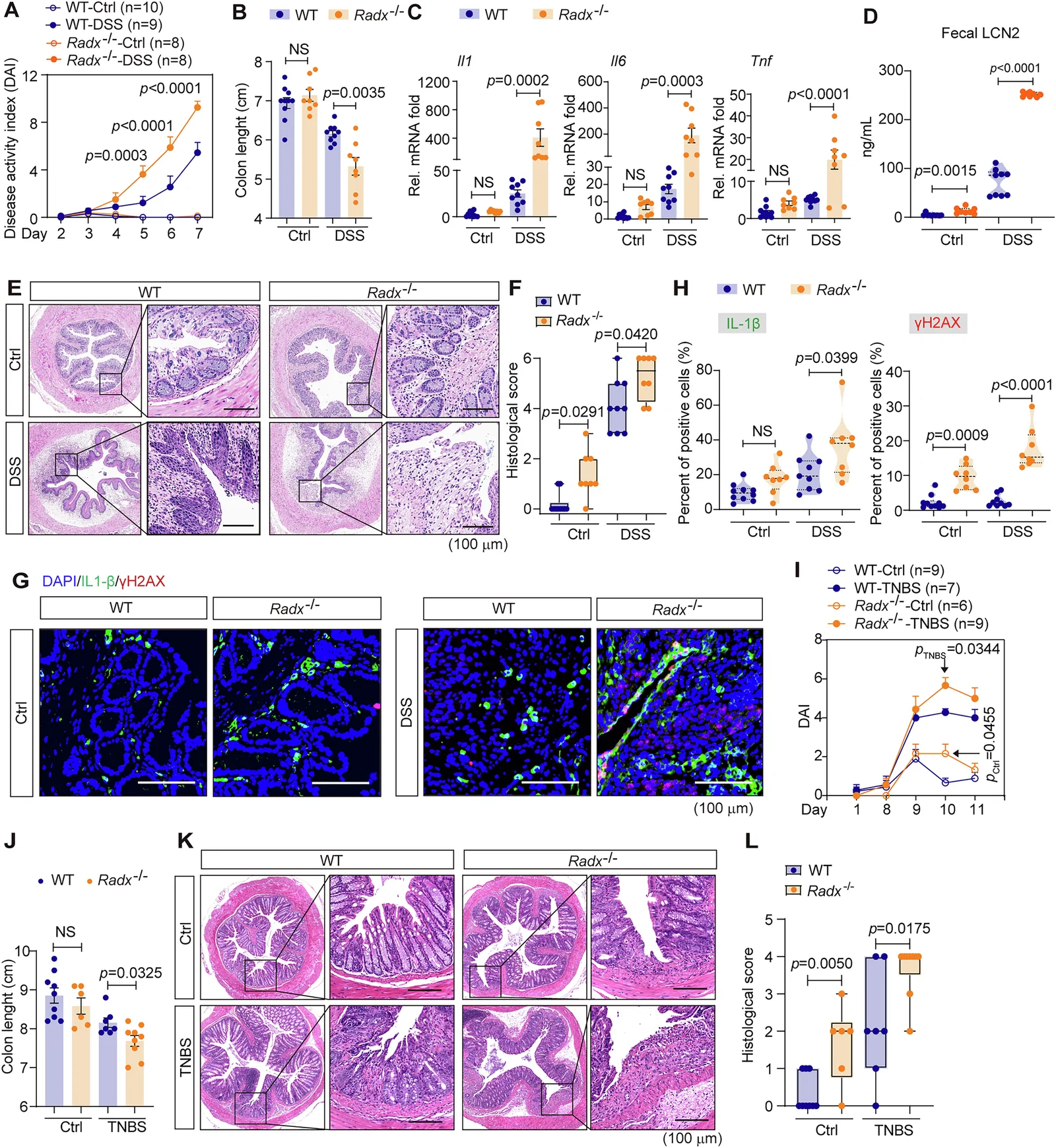
*Radx* deficiency aggravates DSS- and TNBS-induced acute colitis. (**A**, **B**) Disease activity index (DAI) (**A**) and colon lengths (**B**) of WT or *Radx*^−/−^ mice in DSS-induced acute colitis model. *n* = 10 in WT-Ctrl group, *n* = 9 in WT-DSS group, *n* = 8 in *Radx*^−/−^-Ctrl group, and *n* = 8 in *Radx*^−/−^-DSS group. Data were pooled from two independent experiments. (**C**) Real-time PCR of distal colonic homogenates of WT or *Radx*^−/−^ mice from DSS-induced acute colitis model of two independent experiments. Each data point represents one biologically independent individual. (**D**) Fecal LCN2 detection of WT or *Radx*^−/−^ mice from DSS-induced acute colitis model of two independent experiments. Each data point represents one biologically independent individual. (**E**–**H**) Representative images of hematoxylin-eosin (H&E) staining (**E**), histological scores (**F**), immunofluorescence analysis (**G**), and quantification of immunofluorescence staining (**H**) of distal colon segments from WT or *Radx*^−/−^ mice from DSS-induced acute colitis model of two independent experiments. Each data point represents one biologically independent individual. (**I**–**L**) DAI (**I**), colon lengths (**J**), representative images of H&E staining (**K**), and histological scores (**L**) of WT or *Radx*^−/−^ mice in TNBS-induced acute colitis model. *n* = 9 in WT-Ctrl group, *n* = 7 in WT-TNBS group, *n* = 6 in *Radx*^−/−^-Ctrl group, and *n* = 9 in *Radx*^−/−^-TNBS group of two independent experiments. Each data point represents one biologically independent individual. Data were presented as mean ± SEM and analyzed using one-way (**B**, **D**, **F**, **H**, **J**, **L**) or two-way (**A**, **I**) ANOVA followed by Tukey–Kramer multiple-comparisons test. In box plot, the centre line represents the median, the box boundaries represent the 25th and 75th percentiles, the whiskers extend from the minimum to the maximum values, and all individual data points are shown. The exact *P* values are indicated in the figures. Source data are available online for this figure.

In the DSS-induced model, *Radx*^*−/−*^ mice exhibited increased disease activity index (DAI) and reduced colon length compared to wild-type (WT) littermates (Fig. 1A,B). This was accompanied by elevated mRNA levels of proinflammatory cytokines in colonic tissue and higher fecal lipocalin-2 (LCN2) (Fig. 1C,D). Histological analysis and immunofluorescence staining revealed exacerbated pathology and increased levels of IL-1β and γH2AX in distal colons of *Radx*^*−/−*^ (Fig. 1E–H). Similarly, in the TNBS/ethanol-induced acute colitis model, *Radx*^*−/−*^ mice developed more severe clinical and histological damage (Fig. 1I–L). Notably, the elevated histological scores and fecal LCN2 under basal conditions suggested spontaneous inflammation in *Radx*^−/−^ mice (Fig. 1D,F,L). Collectively, these results demonstrate that *Radx* deficiency aggravates colitis in mice.

We further examined the chronic colitis phenotype in *Radx*^−/−^ mice using repeated DSS administration (Wirtz et al, 2017) (Fig. EV1C). Although colon length did not differ between WT and *Radx*^−/−^ mice after 3 cycles of DSS/water administration, we observed increased DAI, histological scores, and IL-1β and γH2AX signals in distal colon of *Radx*^−/−^ mice (Fig. EV1D–I), which is consistent with acute colitis phenotype. However, whether RADX contributes to chronic, relapsing colitis is needed further confirmation using genetically manipulated mouse model (e.g., *Il10*^*−/−*^ mice and *Muc1/2*^−/−^ mice) (Valatas et al, 2013).

### *Radx* deficiency exacerbates colitis development dominantly via macrophages

**Figure 2:**
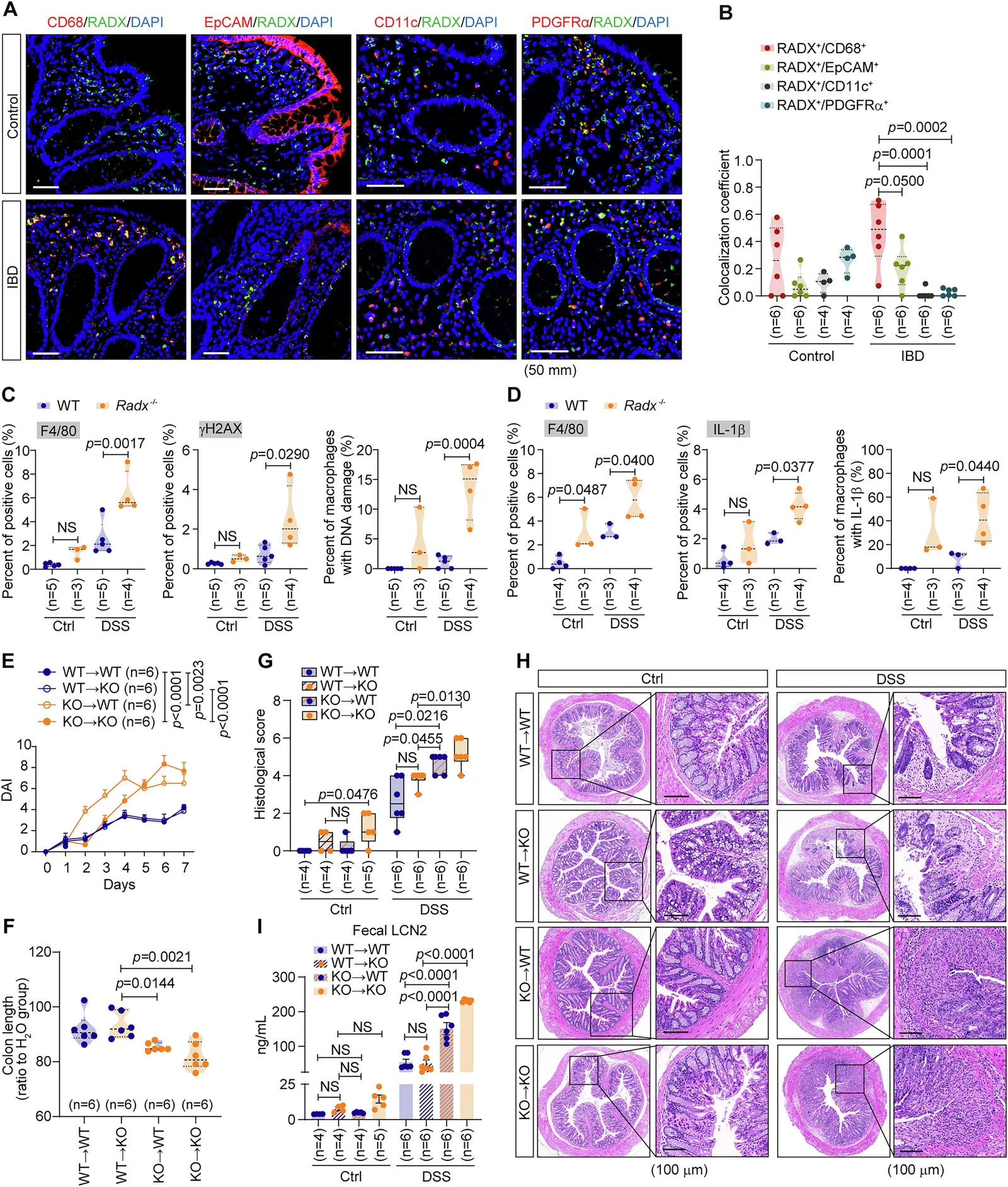
RADX regulates colitis development via hematopoietic cells. (**A**, **B**) Immunofluorescence staining (**A**) and quantification (**B**) of colonic biopsies from control and IBD patients. Each data point represents one biologically independent individual. (**C**) Immunofluorescence (F4/80 and γH2AX) quantifications of distal colon from WT or *Radx*^−/−^ mice in DSS-induced colitis model. *n* = 5 in WT-Ctrl group, *n* = 5 in WT-DSS group, *n* = 3 in *Radx*^−/−^-Ctrl group, and *n *= 4 in *Radx*^−/−^-DSS group. Each data point represents a measurement from independent individual. (**D**) Immunofluorescence (F4/80 and IL-1β) quantifications of distal colon from WT or *Radx*^−/−^ mice in DSS-induced colitis model. *n* = 4 in WT-Ctrl group, *n* = 3 in WT-DSS group, *n* = 3 in *Radx*^−/−^-Ctrl group, and *n* = 4 in *Radx*^−/−^-DSS group. Each data point represents one biologically independent individual. (**E**–**H**) Four groups of bone marrow transplantation were generated with WT or *Radx*^−/−^ mice and subjected to DSS-induced colitis model. DAI (**E**), colon length changes (**F**), histological scores (**G**), representative images of H&E staining (**H**), and fecal LCN2 (**I**) were shown. *n* = 4 for the WT → WT-Ctrl, WT → KO-Ctrl, and KO → WT-Ctrl groups; *n* = 5 for KO → KO-Ctrl group; and *n* = 6 for all DSS-treated groups. Each data point represents one biologically independent individual. ※ As the experimental groups exhibited significant differences in baseline colon length following bone marrow transplantation, we accounted for this variation by normalizing the post-treatment colon length of each group to the average initial length of its respective control. This normalization yielded a change ratio for comparative analysis. Data were presented as mean ± SEM and analyzed using one-way (**B**–**D**, **F**, **I**) or two-way (**E**) ANOVA followed by Tukey–Kramer multiple-comparisons test, or two-tailed Mann–Whitney *U* test (**G**). The exact *P* values are indicated in the figures. Source data are available online for this figure.

**Figure EV2:**
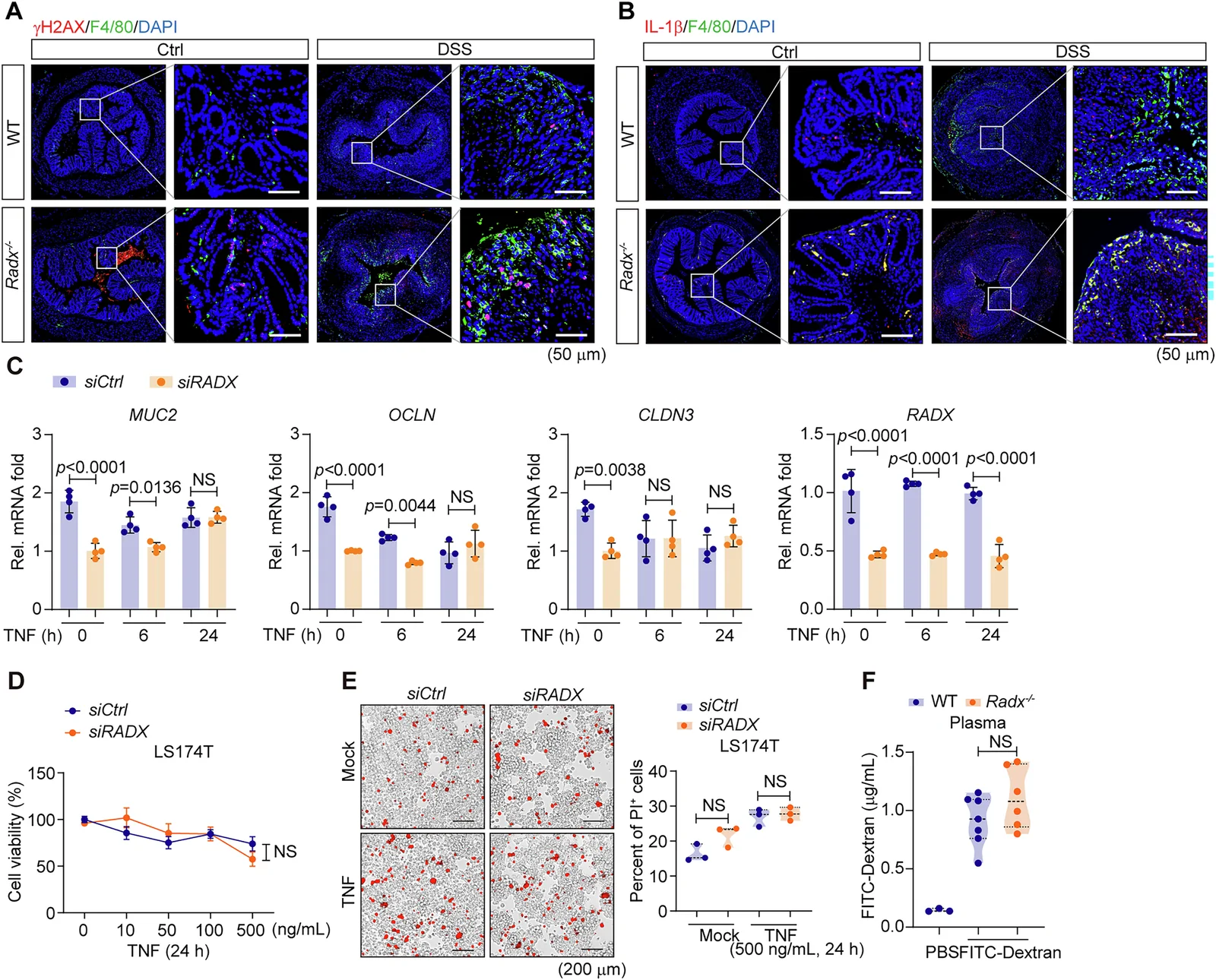
RADX function in intestinal epithelial cells. (**A**, **B**) Representative immunofluorescence images of distal colon from WT or *Radx*^−/−^ mice in DSS-induced colitis model. Quantification results were shown in Fig. 2C,D, respectively. (**C**) LS174T cells transfected with *siCtrl* or *siRADX* were treated with TNF (100 ng/mL) for indicated time course. Real-time PCR was conducted to analyze the mRNA expression. Data are from *n* = 3 independent biological replicates. (**D**) LS174T cells transfected with *siCtrl* or *siRADX* were treated with indicated dosage of TNF for 24 h. Cell viability was assessed by CCK-8 assay. Data are from *n* = 3 independent biological replicates. (**E**) LS174T cells transfected with *siCtrl* or *siRADX* were treated with TNF (500 ng/mL) for 24 h. Cell death was assessed by propidium iodide (PI) staining and fluorescence microscopy. Data are from *n* = 3 independent biological replicates. (**F**) 8-week-old mice were administered with FITC-dextran (600 mg/kg) by oral gavage. After 4 h blood samples were collected and plasma FITC fluorescence was measured using a fluorescence microplate reader. Each data point represents one biologically independent individual. Data were presented as mean ± SEM and analyzed using one-way ANOVA followed by Tukey–Kramer multiple-comparisons test. The exact *P* values are indicated in the figures. Source data are available online for this figure.

To identify the cell type responsible for RADX-mediated colitis, we first analyzed RADX expression in colonic biopsies from healthy controls and PIBD patients. RADX was predominantly expressed in macrophages (CD68^+^), with minimal expression detected in epithelial cells (EpCAM^+^), dendritic cells (CD11c^+^), or stromal cells (PDGFRα^+^) (Fig. 2A,B). Consistently, F4/80^+^ macrophages from *Radx*^−/−^ mice exhibited significantly elevated levels of γH2AX and IL-1β (Figs. 2C,D and  EV2A,B), suggesting that *Radx* deficiency may promote colitis through macrophages.

To determine the contribution of hematopoietic cells, we performed the bone marrow transplantation (BMT). In DSS-induced colitis models, mice receiving *Radx*^−/−^ bone marrow cells (KO → WT and KO → KO groups) developed more severe disease, as evidenced by significantly higher disease activity indices, greater colon shortening, increased histological scores, and elevated fecal LCN2 compared to mice receiving WT bone marrow cells (Fig. 2E–I).

We further investigated whether RADX deficiency impacts the expression of intestinal barrier proteins and epithelial cell death. In human goblet cell line LS174T, siRNA-mediated *RADX* knockdown reduced the expression of MUC2, occludin (OCLN), and CLDN3 under basal conditions; however, these effects were diminished under inflammatory stimulation (Fig. EV2C). Moreover, *RADX* deficiency did not significantly affect cell death (Fig. EV2D,E). This is consistent with the observation that *Radx*^−/−^ mice did not exhibit increased intestinal barrier permeability, as measured by a plasma FITC-Dextran assay, compared to WT mice (Fig. EV2F).

Taken together, these results suggest that Radx deficiency in macrophages accounts for much of the exacerbated colitis phenotype. However, contributions from additional cell types, including epithelial cells, are not yet fully excluded and require further investigation.

### *RADX* deficiency promotes NF-κB pathway activation

**Figure 3:**
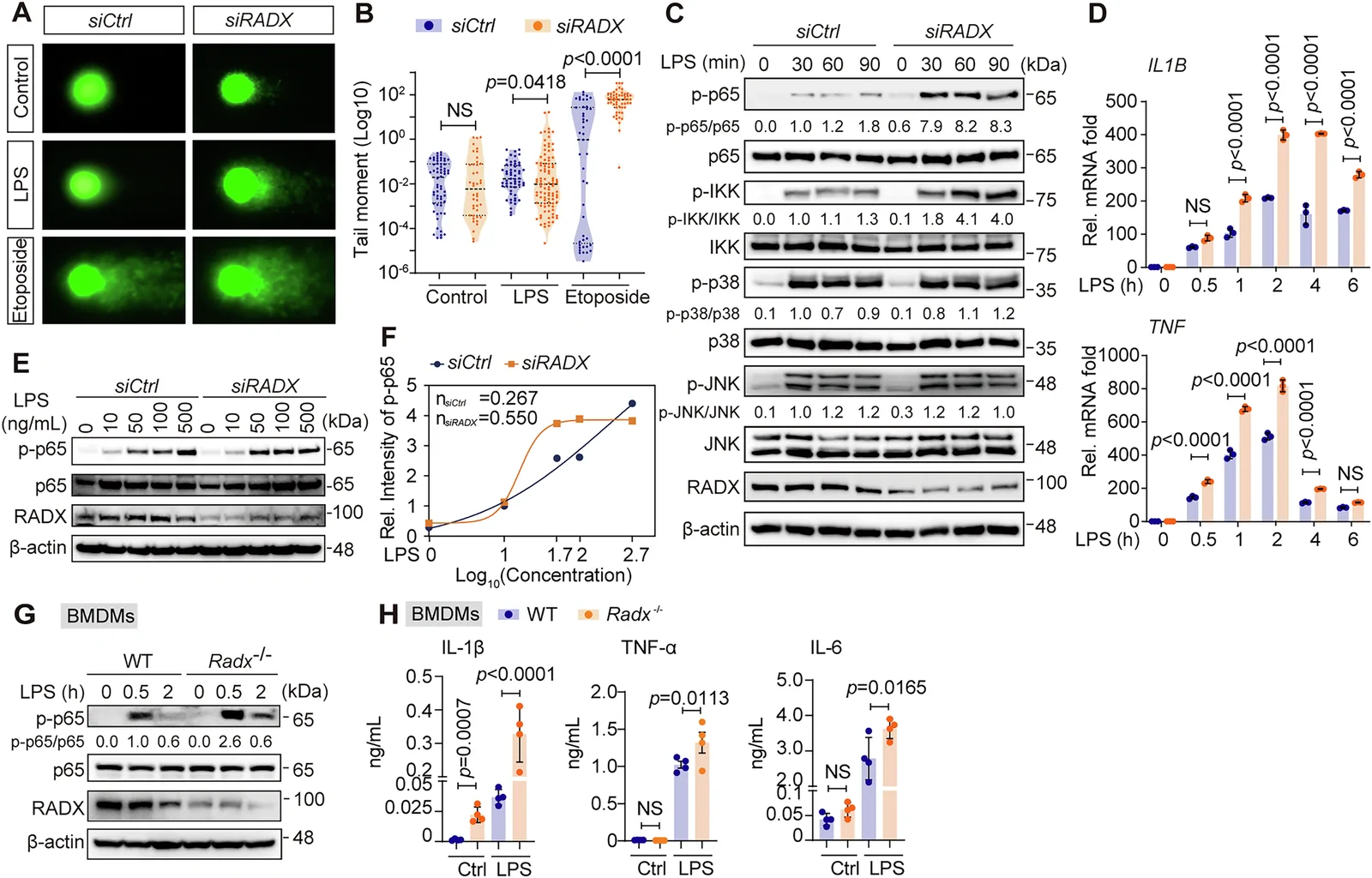
*RADX* deficiency promotes NF-κB activation. (**A**, **B**) Representative images of comet assay (**A**) of THP-1 cells transfected with *siRADX* compared with those transfected with *siCtrl* under LPS or etoposide (50 μM, the same concentration was used in following experiments, unless otherwise specified) stimulation for 2 h. The tail moments statistics were performed with Comet Assay Software Project (CASP) (**B**). Each data point represents a measurement from independent cell. *n* = 62 for *siCtrl*-control, *n* = 42 for *siRADX*-control, *n* = 77 for *siCtrl*-LPS, *n* = 97 for *siRADX*-LPS, *n* = 45 for *siCtrl*-Etoposide, and *n* = 54 for *siRADX*-Etoposide. (**C**, **D**) Immunoblotting analysis (**C**) and real-time PCR analysis (**D**) of macrophages transfected with *siCtrl* or *siRADX* and treated with LPS for indicated time course. Data are from *n* = 3 independent biological replicates. (**E**, **F**) Macrophages transfected with *siCtrl* or *RADX*-specific siRNA were stimulated with indicated doses of LPS for 0.5 h. Cell lysates were harvested for immunoblotting (**E**) and hill coefficient of p-p65 intensity was shown (**F**). (**G**, **H**) Bone-marrow-derived macrophages (BMDMs) of WT or *Radx*^−/−^ mice were treated with LPS for indicated time (**G**) or 6 h (**H**). Cell lysates were subjected to immunoblotting (**G**) and supernatants were collected for ELISA (**H**). *n* = 3 biological replicates for ELISA. Data were presented as mean ± SEM and analyzed using one-way ANOVA (**D**, **H**) followed by Tukey–Kramer multiple-comparisons test, or two-tailed Mann–Whitney *U* test (Fig. 3B). All immunoblotting assays were repeated at least three times with independent biological replicates. The exact *P* values are indicated in the figures. Source data are available online for this figure.

**Figure EV3:**
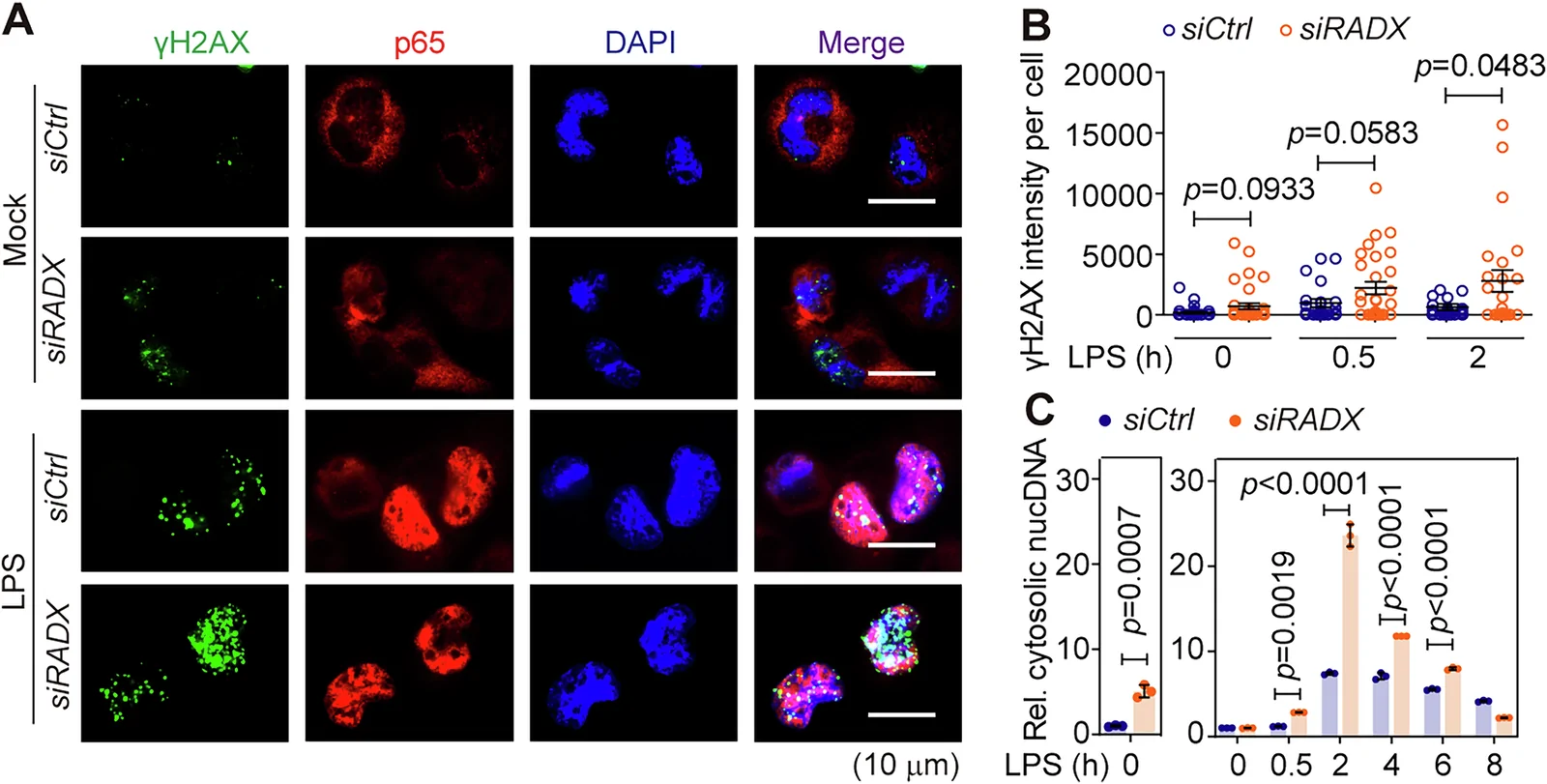
RADX deficiency induces DNA damage in macrophages. (**A**, **B**) Representative images (**A**) and quantification result (**B**) of confocal fluorescence analysis of THP-1-derived macrophages transfected with *siCtrl* or *RADX-*specific siRNA and stimulated with LPS for 2 h. *n* = 31 for *siCtrl*-Mock group, *n* = 36 for *siCtrl*-LPS 0.5 h group, *n* = 30 for *siCtrl*-LPS 2 h group, *n* = 29 for *siRADX*-Mock group, *n* = 36 for *siRADX*-LPS 0.5 h group, and *n* = 24 for *siRADX*-LPS 2 h group. Each data point represents a measurement from independent cell. (**C**) THP-1-derived macrophages transfected with *siCtrl* or *RADX-*specific siRNA were stimulated with LPS for indicated time. Cytoplasmic DNA were extracted for quantitative PCR analysis using nuclear DNA primers (Tert). Data are from *n* = 3 independent biological replicates. Data were presented as mean ± SEM and analyzed using one-way ANOVA (**C**) followed by Tukey–Kramer multiple-comparisons test, or two-tailed Mann–Whitney *U* test (**B**). The exact *P* values are indicated in the figures. Source data are available online for this figure.

Given that *RADX*-deficiency cells accumulate DSBs (Bhat et al, 2018), and nuclear DNA damage activates ATM, and IFI16 mediates NF-κB signaling (Dunphy et al, 2018), we sought to establish a causal link between RADX deficient and hyperinflammation. Upon LPS or etoposide stimulation, *RADX*-deficiency THP-1-derived macrophages (hereafter referred to as macrophages) displayed a significant increase in DNA damage, as measured by comet assay and γH2AX foci formation (Figs. 3A,B and EV3A,B). Cytosolic nuclear DNA levels were significantly elevated in *RADX*-deficient compared with WT macrophages (Fig. EV3C).

In *RADX*-deficiency macrophages, we observed significantly elevated phosphorylated p65 and mRNA levels of proinflammatory cytokines (Fig. 3C,D). Furthermore, *RADX*-deficient rendered macrophages more sensitive to LPS treatment, as indicated by a leftward shift in the dose-response curve (Fig. 3E,F; Hill coefficient, n_*siCtrl*_ = 0.267, n_*siRADX*_ = 0.550). Consistently, bone marrow derived macrophages (BMDMs) from *Radx*-deficiency mice exhibited increased p65 phosphorylation and inflammatory cytokines secretion (Fig. 3G,H).

These results demonstrated that RADX-deficient in macrophages leads to increased DNA damage, accompanied with hyperactivation and hypersensitization of the NF-κB signaling.

### RADX competes with IFI16 for ssDNA binding

**Figure 4:**
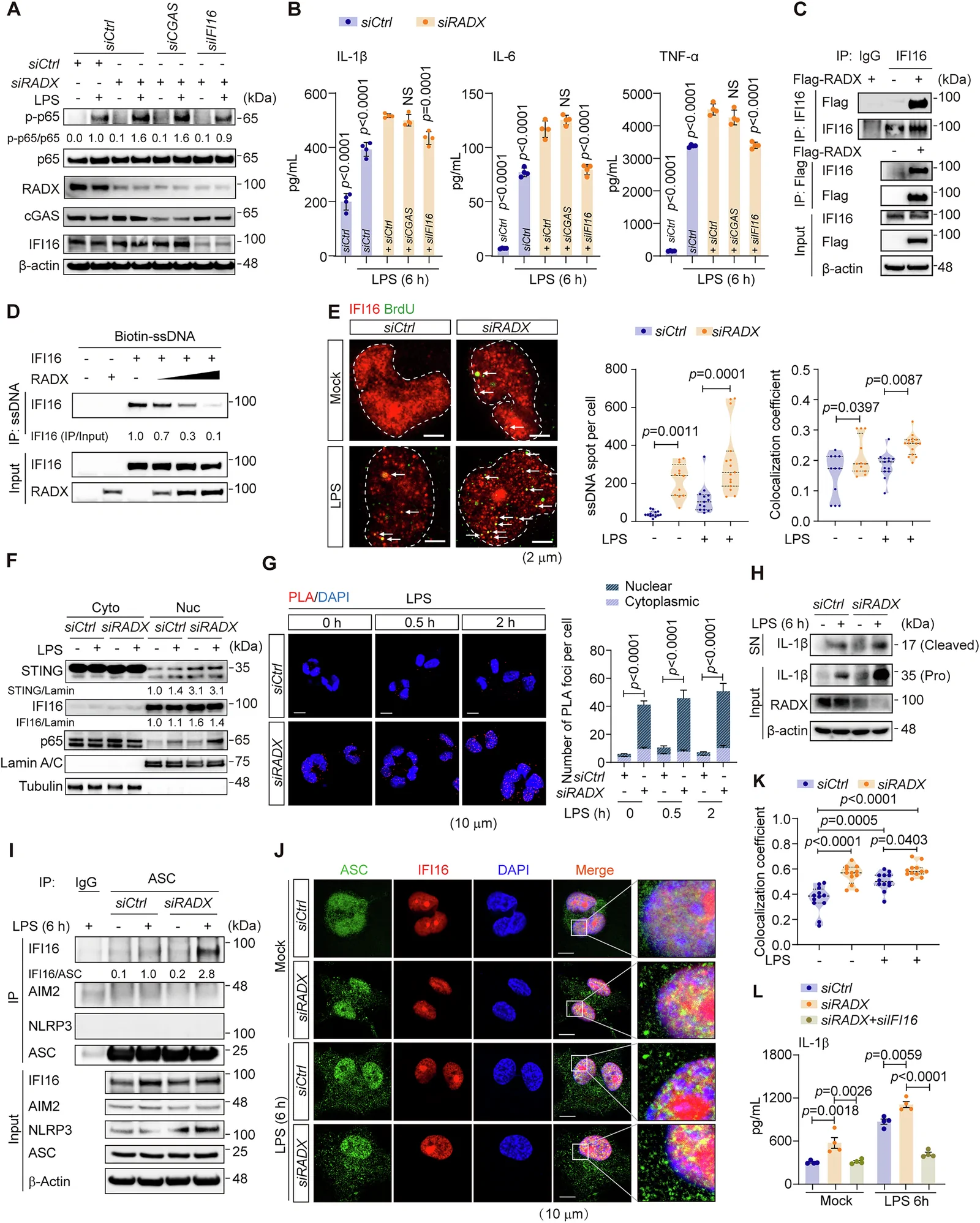
RADX inhibits IFI16-meidated NF-κB activation and inflammasome assembly by competing DNA binding. (**A**, **B**) *siCtrl-* or *siRADX-*transfected macrophages were co-transfected with *Ctrl*, *CGAS*, or *IFI16*-specific siRNA and treated with LPS for 0.5 h (**A**) or 6 h (**B**). *n* = 3 biological replicates for ELISA. Significance test was versus to group *siCtrl* & *siRADX* with LPS stimulation. (**C**) Immunoprecipitation and immunoblotting analysis of HEK 293 T cells transfected with Flag-RADX plasmids. (**D**) In vitro ssDNA binding assay of IFI16 with increasing dose of RADX protein (1  μg, 10 μg, and 50 μg). (**E**) Representative confocal imaging (left) and statistical analysis (right) of macrophages transfected with *siCtrl* or *siRADX* and pre-labelled with BrdU for 48 h. LPS was applied for stimulation for 2 h. The dashed lines represented the position of nuclear membranes. The corresponding images of different channels are shown in the Fig. EV4E. Each data point represents a measurement from independent cell. *n* = 11 for *siCtrl*-Mock group, *n* = 12 for *siRADX*-Mock group, *n* = 13 for *siCtrl*-LPS group, and *n* = 15 for *siRADX*-LPS group. (**F**) Immunoblotting analysis of cytosolic (Cyto) and nuclear (Nuc) fractions from *siCtrl* or *siRADX* transfected macrophages treated with LPS for 30 min. (**G**) Proximity Ligation Assay (PLA) of *siCtrl* or *siRADX* transfected macrophages treated with LPS for indicated time. Red foci indicate IFI16-STING interaction. Each data point represents a measurement from independent cell. *n* = 31 for *siCtrl*-Mock group, *n* = 34 for *siCtrl-*LPS 0.5 h group, *n* = 37 for *siCtrl*-LPS 2 h group, *n* = 33 for *siRADX*-Mock group, *n* = 21 for *siRADX*-LPS 0.5 h group, and *n* = 31 for *siRADX*-LPS 2 h group. (**H**) Macrophages transfected with *siCtrl* or *RADX*-specific siRNA were stimulated with LPS for 6 h. Cell lysates and concentrated protein from supernatant were harvested for immunoblotting. (**I**) *siCtrl* or *siRADX* transfected macrophages were treated with LPS for 6 h. Cell lysates were subjected to immunoblot and co-immunoprecipitation with ASC antibody. (**J**, **K**) Representative images (**J**) and statistical analysis (**K**) of immunofluorescence of *siCtrl* or *siRADX* transfected macrophages treated with LPS for 6 h with confocal microscope. Each data point represents a measurement from independent cell. *n* = 12 for each group. (**L**) Macrophages transfected with *siCtrl*, *siRADX* or *siRADX* together with *siIFI16* were stimulated with LPS for 6 h. Supernatants were collected for ELISA. *n* = 3 biological replicates for ELISA. Data were presented as mean ± SEM and analyzed using one-way ANOVA followed by Tukey–Kramer multiple-comparisons test. All immunoblotting assays were repeated at least three times with independent biological replicates. The exact *P* values are indicated in the figures. Source data are available online for this figure.

**Figure EV4:**
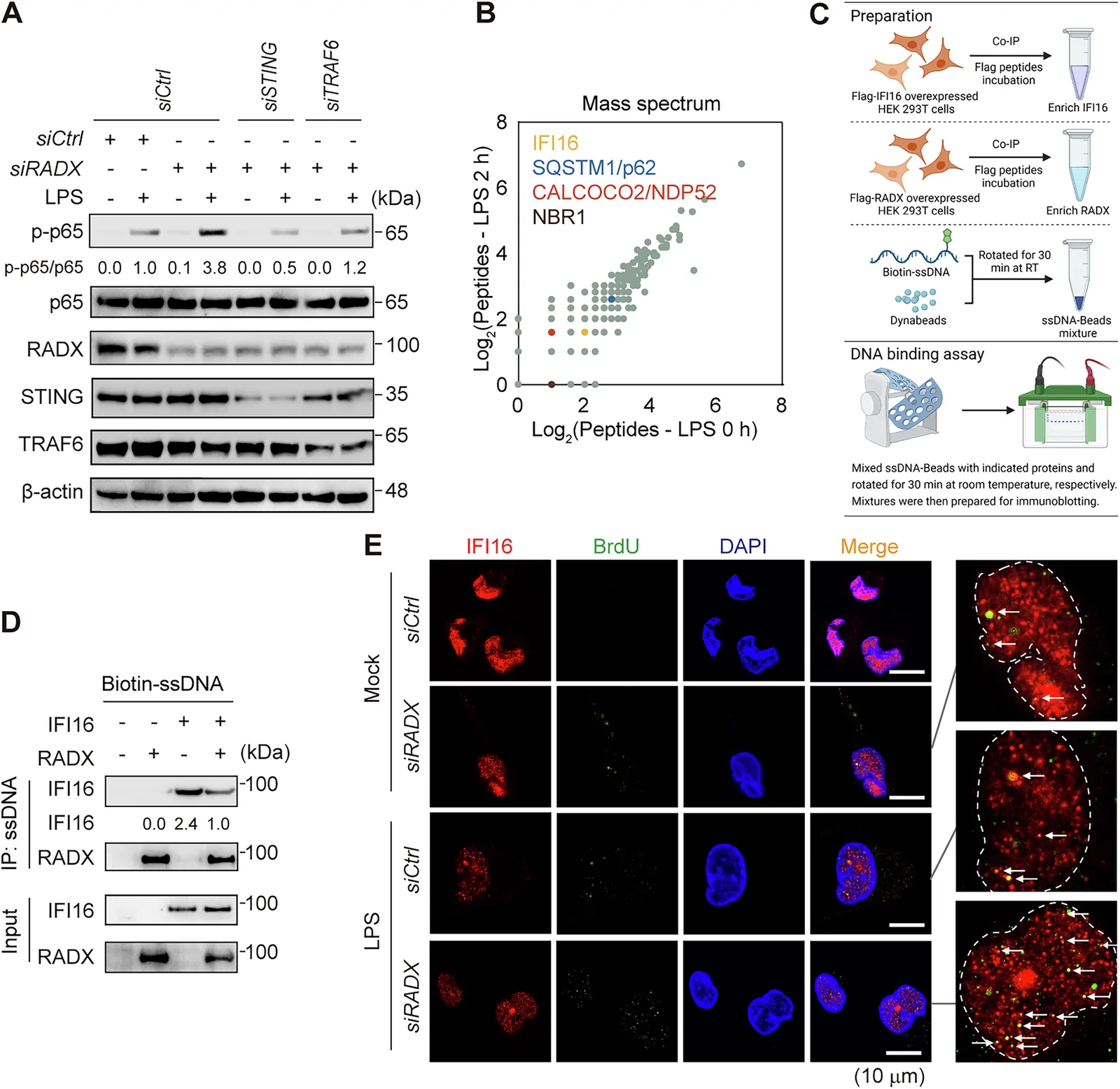
RADX regulates NF-κB pathway activation. (**A**) *siCtrl-* or *siRADX-*transfected THP-1-derived macrophages were co-transfected with *Ctrl*, *STING*, or *TRAF6*-specific siRNA and treated with LPS for 0.5 h. (**B**) Endogenous RADX in THP-1-derived macrophages was enriched by co-immunoprecipitation for mass spectrum analysis. (**C**) Workflow for in vitro ssDNA binding assay. (**D**) In vitro ssDNA binding assay of IFI16 in the presence (10 μg enriched protein for each) or absence of RADX. (**E**) Representative confocal imaging of macrophages transfected with *siCtrl* or *siRADX* and pre-labelled with BrdU for 48 h. LPS was applied for stimulation for 2 h. The dashed lines represented the position of nuclear membranes. Immunoblotting assays were repeated at least three times with independent biological replicates. Source data are available online for this figure.

As reported, the IFI16-STING pathway is critical for DNA damage-induced NF-κB pathway hyperactivation (Dunphy et al, 2018). IFI16 silencing abolished the NF-κB hyperactivation caused by RADX deficiency (Figs. 4A,B and  EV4A). Given that RADX binds with ssDNA (Dungrawala et al, 2017), we hypothesized that RADX might regulate ssDNA sensing by IFI16. Mass spectrometry (Fig. EV4B) and co-immunoprecipitation (Fig. 4C) confirmed an interaction between RADX and IFI16. In vitro DNA binding assay further demonstrated that RADX inhibits the binding of IFI16 to ssDNA (Figs. EV4C,D and  4D). Accordingly, knockdown of endogenous RADX in macrophages led to increased formation of ssDNA foci and enhanced IFI16-ssDNA association in nucleus compared with controls (Fig. 4E). By immunoblotting cytosolic (Cyto) and nuclear (Nuc) fractions, we observed increased STING abundance upon LPS treatment in control macrophages, whereas RADX deficiency significantly increased nuclear-localized STING (Fig. 4F). STING is known to be recruited to and interact with IFI16, triggering downstream signaling, upon DNA stimulation (Unterholzner et al, 2010). To further confirm the STING-IFI16 interaction, we performed a proximity ligation assay (PLA) and found that RADX deficiency significantly increased the STING-IFI16 interaction in the nucleus (Fig. 4G). These findings indicate that RADX competes with IFI16 for ssDNA binding and restricts IFI16-STING mediated signaling.

IFI16 recognizes DNA to activate not only the non-canonical NF-κB pathway (Dunphy et al, 2018), but also the assembly of IFI16 inflammasome (Jin et al, 2012). Upon LPS treatment, RADX-deficiency macrophages showed elevated levels of both pro-IL-1β and cleaved-IL-1β (Fig. 4H), prompting us to investigate whether RADX regulates IFI16 inflammasome assembly. Co-immunoprecipitation and confocal immunofluorescence results revealed that RADX deficiency enhanced ASC-IFI16 interaction (Fig. 4I–K). Moreover, silencing IFI16 in RADX-deficiency macrophages abolished excessive production of IL-1β (Fig. 4L).

Collectively, these data demonstrate that RADX competes with IFI16 for ssDNA binding, thereby suppressing IFI16-dependent NF-κB pathway activation and inflammasome assembly.

### RADX mutations identified from PIBD cohort

**Figure 5:**
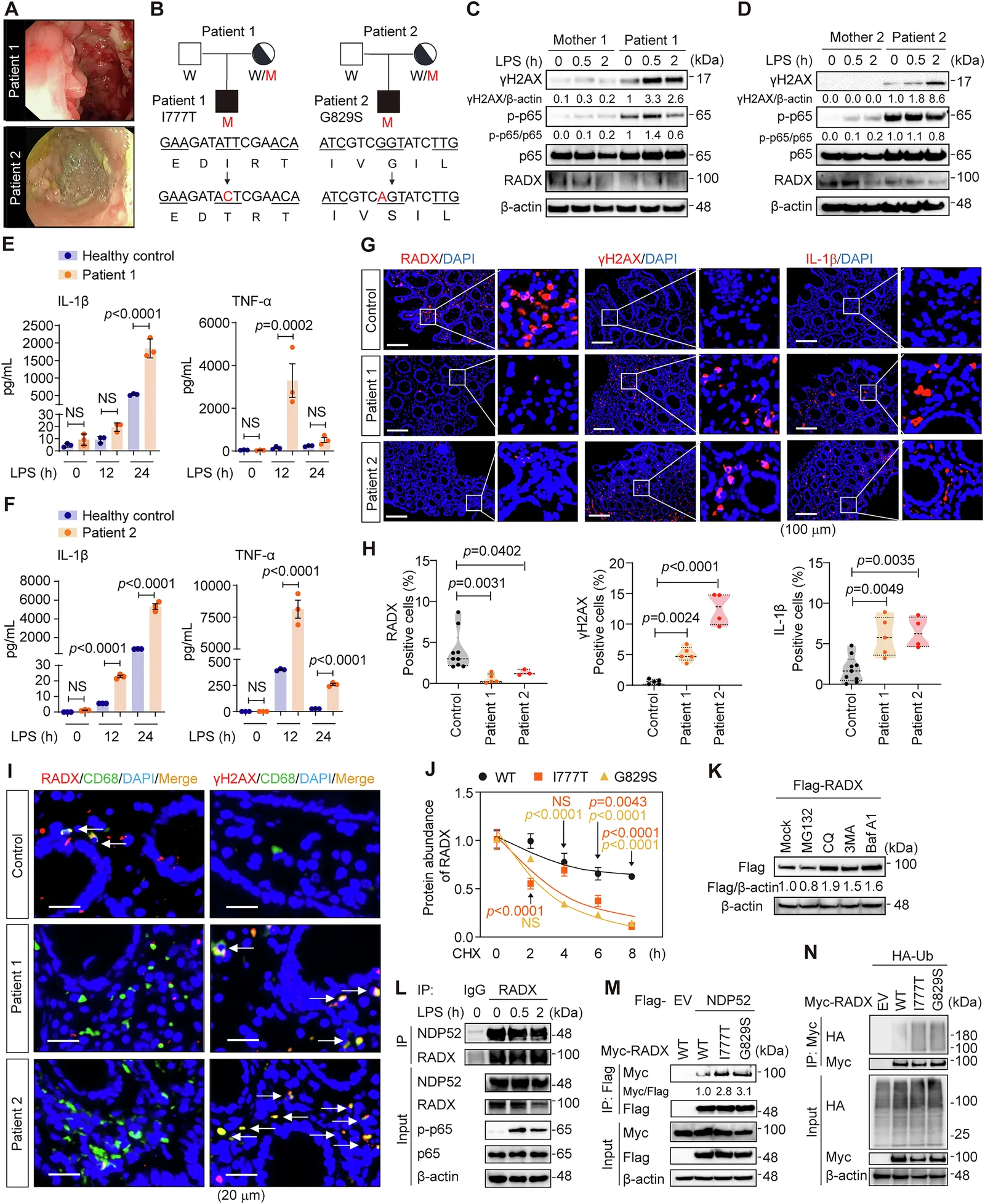
Variants promote autophagic degradation of RADX, leading to excessive DNA damage and hyperinflammation. (**A**) Endoscopic images showing relapsing CD-like presentations in the patients with RADX mutations. (**B**) Inheritance of *RADX* mutations in the two CD patients. M mutant allele, W wild-type allele. (**C**, **D**) Immunoblotting analysis of PBMCs from the patients and their mothers treated with LPS (500 ng/mL) at indicated time. (**E**, **F**) PBMCs from the patients and healthy controls were treated with LPS (500 ng/mL). Supernatants were collected for ELISA. *n* = 3 biological replicates. (**G**, **H**) Representative immunofluorescence (**G**) and quantitative analysis (**H**) of RADX, γH2AX and IL-1β expression in colonic sections from healthy donors (*n* = 9 individuals), Patient 1 (*n* = 5 separate colonoscopy examinations), and Patient 2 (*n* = 3 separate colonoscopy examinations in RADX detection, and *n* = 4 in γH2AX and IL-1β detection). (**I**) Immunofluorescent analysis of colonic section of control, patient 1 and patient 2. (**J**) Quantification of RADX abundance from cycloheximide (CHX)-treated HEK 293 T cells transfected with Flag-RADX WT, I777T or G829S mutants. CHX working concentration: 100 μg/mL. *n* = 3 biological replicates. (**K**) Cells transfected with Flag-RADX were treated with proteasome inhibitor MG132 (10 μM), or autophagy inhibitors CQ (50 μM), 3MA (10 mM) or Baf A1 (0.2 μM) for 6 h. RADX abundance was analyzed with anti-Flag immunoblotting. (**L**) Co-immunoprecipitation and immunoblotting analysis of macrophages stimulated with LPS (100 ng/mL, the same concentration was used in following experiments, unless otherwise specified) at indicated time. (**M**, **N**) Co-immunoprecipitation and immunoblotting analysis of HEK 293T cells transfected with indicated plasmids. Data were presented as mean ± SEM and analyzed using one-way (**E**, **F**, **H**) or two-way (**J**) ANOVA followed by Tukey–Kramer multiple-comparisons test. Immunoblotting assays in 5K-5N were repeated at least three times with independent biological replicates. The exact *P* values are indicated in the figures. Source data are available online for this figure.

**Figure EV5:**
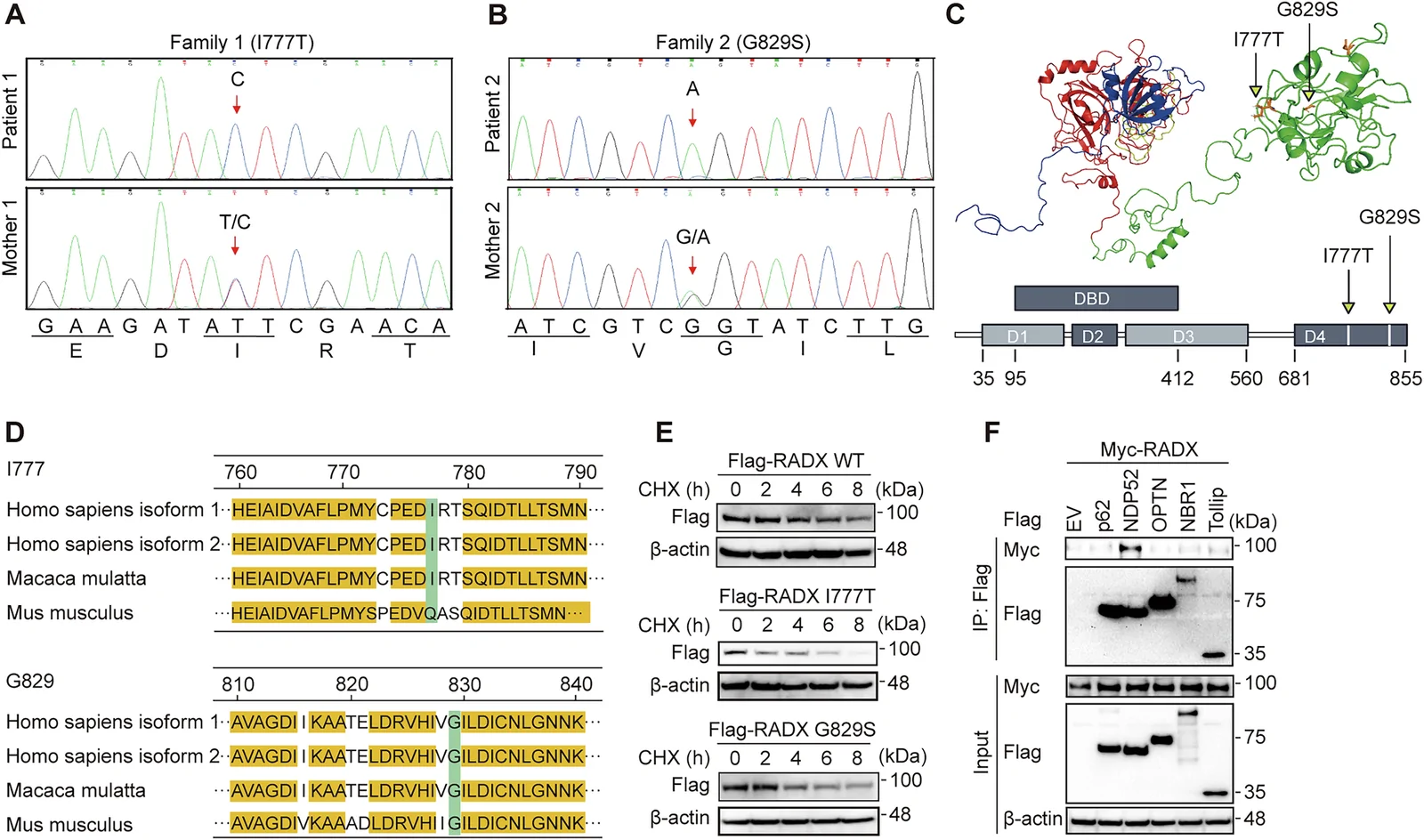
Characteristics of RADX I777T and G829S variants. (**A**, **B**) Sanger sequencing of patient 1 (I777T), patient 2 (G829S) and their mothers. M mutant allele, W wild-type allele. (**C**) Prediction of RADX protein structure by PyMoL. (**D**) Homology analysis of RADX I777T and G829S. (**E**) Immunoblotting of RADX abundance from cycloheximide (CHX)-treated HEK 293T cells transfected with Flag-RADX WT, I777T or G829S mutants. CHX working concentration: 100 μg/mL. *n* = 3 biological replicates. (**F**) HEK 293T cells were transfected with Myc-RADX, together with Flag-EV, p62, NDP52, OPTN, NBR1 or Tollip. Cell lysates were subjected to co-immunoprecipitation and immunoblotting. Immunoblotting assays were repeated at least three times with independent biological replicates. Source data are available online for this figure.

When investigating the clinical relevance of RADX, we identified two missense variants, c.2330 T > C: p.I777T (rs749335623) and c.2485 G > A: p.G829S (rs184295976), in two male patients with Crohn’s disease (CD) (Figs. 5A,B and EV5A,B). I777 and G829 are located in the C-terminal domain of RADX (Fig. EV5C). Homology analysis revealed that I777 is conserved among primates, whereas G829 is conserved across mammals (Fig. EV5D). Compared to their non-IBD mother carriers, peripheral blood mononuclear cells (PBMCs) from both patients exhibited decreased RADX protein expression (Fig. 5C,D). In contrast, PBMCs from patients show markedly elevated levels of γH2AX and p65 phosphorylation upon LPS stimulation (Fig. 5C,D), and secreted significantly higher levels of IL-1β and TNF-α compared to unrelated healthy controls (Fig. 5E,F). Consistent with these findings, colonic biopsies from the patients displayed reduced RADX protein, increased γH2AX and IL-1β signal (Fig. 5G,H). Moreover, reduced RADX level and elevated γH2AX were detected in macrophages (CD68^+^) from patients’ colonic biopsies (Fig. 5I). Thus, these data from the two patients carrying RADX mutations suggest a correlation among reduced RADX expression, increased DNA damage, and hyperactivated inflammatory signaling.

Impaired protein production or accelerated degradation are major causes of decreased protein abundance. By using cycloheximide (CHX) to inhibit new protein synthesis, we observed accelerated degradation rate of RADX variants compared with WT (Figs. 5J and  EV5E). Using proteasome (MG132) and autophagy inhibitors (chloroquine/CQ, 3-methyladenine/3MA, and bafilomycin A1/Baf A1), we found that RADX degradation was primarily mediated by the autophagic pathway (Fig. 5K). Mass spectrum and co-immunoprecipitation analysis revealed that RADX interacts with the cargo receptor NDP52 (Figs. EV4B and 5F,L). The I777T and G829S variants enhanced interactions with NDP52 (Fig. 5M), which may be due to the increased ubiquitination of variants (Fig. 5N). Therefore, I777T and G829S variants accelerate NDP52-dependent autophagic degradation of RADX.

### RADX mutants do not affect RADX-RAD51 binding or replication fork progression

**Figure EV6:**
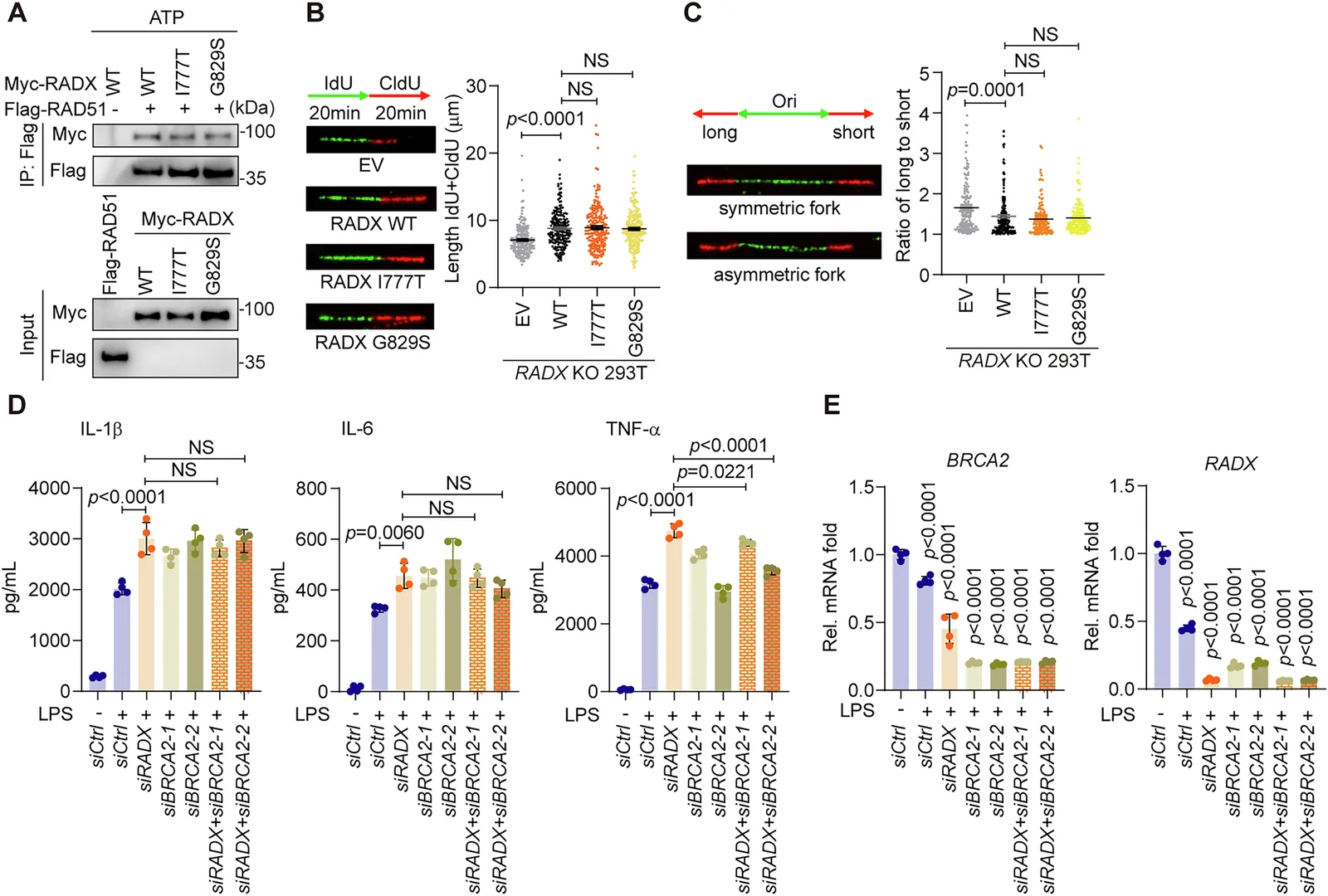
RADX mutations show no effects on RAD51-ATP binding or replication fork progression. (**A**) Purified Myc-RADX WT, I777T, G829S and ATP pre-bound Flag-RAD51 were incubated as indicated for 1 h. Mixtures were subjected to co-immunoprecipitation. (**B**, **C**) *RADX* KO HEK 293T cells transfected with EV, RADX WT, I777T or G829S were labeled with CldU and IdU as indicated and analyzed by DNA combing. Each data point represents a measurement from independent individual. *n* = 201 for each in (**B**); *n* = 159 for EV group, *n* = 172 for WT group, *n* = 134 for I777T group, *n* = 129 for G829S group in (**C**). (**D**) THP-1-derived macrophages transfected with *Ctrl*-, *RADX*- or *BRCA2*- specific siRNA were treated with LPS for 6 h. Supernatant were collected for ELISA. Data are from *n* = 4 independent biological replicates. (**E**) Knockdown efficiency of THP-1-derived macrophages transfected with *Ctrl*-, *RADX*- or *BRCA2*- specific siRNA with real-time PCR. Data are from *n* = 4 independent biological replicates. Data were presented as mean ± SEM and analyzed using one-way ANOVA followed by Tukey–Kramer multiple-comparisons test (**D**, **E**), or two-tailed Mann–Whitney *U* test (**B**, **C**). The exact *P* values are indicated in the figures. Source data are available online for this figure.

To evaluate whether RADX variants affect their interaction with RAD51, we tested RADX-RAD51 binding and replication fork progression. First, we used purified proteins in an in vitro binding assay (Adolph et al, 2021) and found that the I777T and G829S variants did not interfere with RADX binding to ATP-bound RAD51 (Fig. EV6A). Next, we performed a DNA fiber assay with iododeoxyuridine (IdU) and chlorodeoxyuridine (CldU) labeling in *RADX* knockout (KO) 293T cells. *RADX* deficiency significantly decreased the replication elongation rate and increased asymmetric fork elongation compared to WT RADX-reconstituted cells (Fig. EV6B,C). However, no differences were observed between WT and mutant RADX in replication fork elongation rates or fork symmetry (Fig. EV6B,C).

BRCA2 has been proposed to facilitate RAD51 nucleofilament formation; however, *BRCA2* deficiency does not affect replication-associated DSB formation in *RADX*-deficiency cells (Davies and Pellegrini, 2007; Dungrawala et al, 2017; Esashi et al, 2007). To investigate whether BRCA2 regulates hyperinflammation in *RADX*-deficiency cells, we performed siRNA-mediated knockdown of *BRCA2*. We found that *BRCA2*-deficiency promoted inflammatory cytokine expression in LPS-stimulated THP-1-derived macrophages. However, *BRCA2* knockdown did not alter the inflammatory response in *RADX*-deficiency cells (Fig. EV6D,E).

Taken together, these results indicate that the RADX I777T and G829S mutants reduce RADX protein stability but retain activity in RAD51-ATP binding and DNA replication progression. Furthermore, BRCA2 depletion does not rescue LPS-induced hyperinflammation in *RADX*-deficiency macrophages.

### Chemical inhibition of RAD51 alleviates genomic instability, inflammation, and colitis severity

**Figure 6:**
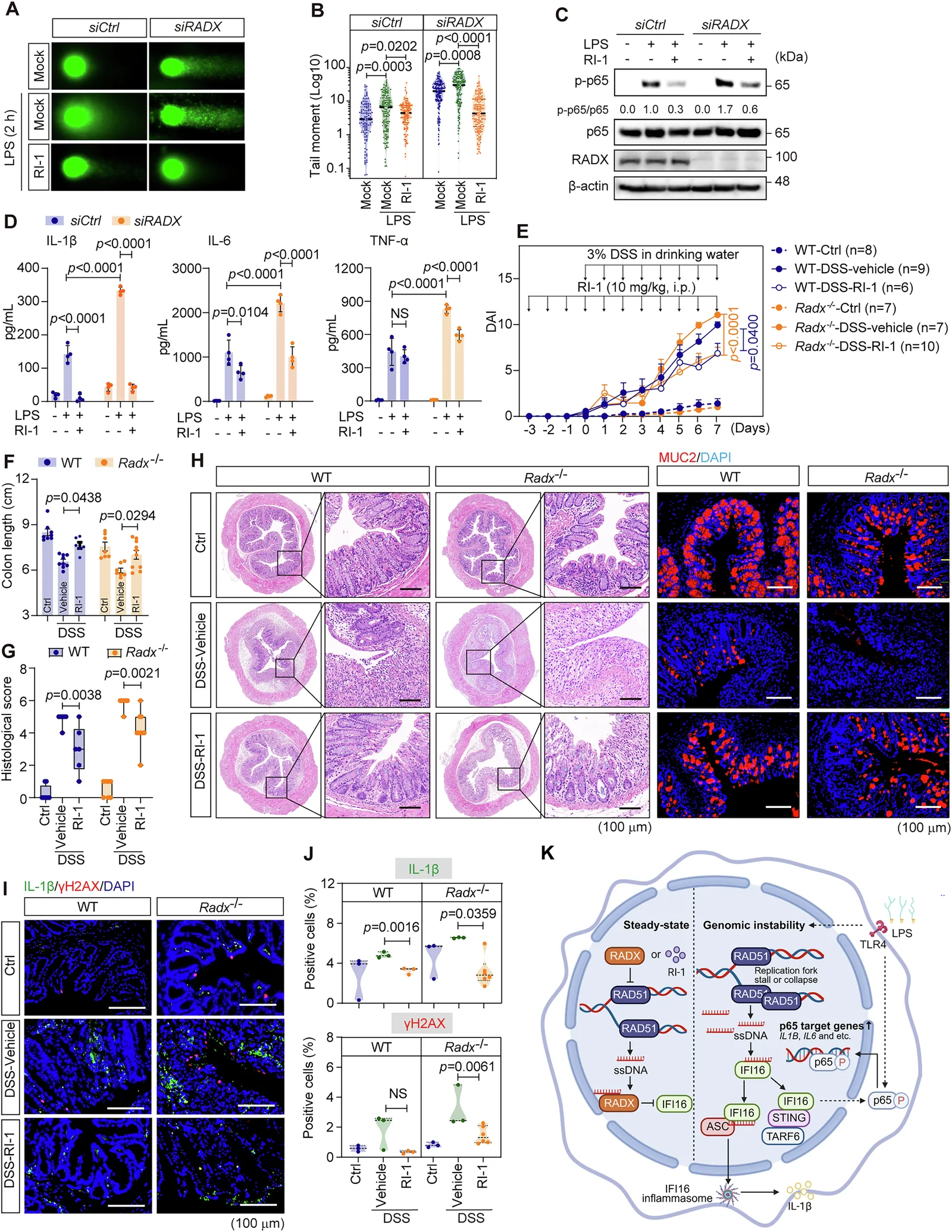
Chemical inhibition of RAD51 alleviates colitis severity of WT and *Radx*^−/−^ mice. (**A**, **B**) Representative comet assay (**A**) and tail moment statistics (**B**) showing that treatment with RI-1 (25 μM for 6 h) significantly decreased DNA damage compared with untreated groups in both *siCtrl* or *siRADX*-transfected THP-1 cells under LPS stimulation for 2 h. Each data point represents a measurement from independent cell. *n* = 110 for *siCtrl*-Mock group, *n* = 138 for *siCtrl*-LPS group, *n* = 130 for *siCtrl*-LPS with RI-1 treatment group, *n* = 166 for *siRADX*-Mock group, *n* = 133 for *siRADX*-LPS group, and *n* = 149 for *siRADX*-LPS with RI-1 treatment group. (**C**, **D**) Immunoblotting (**C**) and ELISA (**D**) of macrophages transfected with *siCtrl* or *siRADX* and treated with RI-1 for 6 h and followed with LPS stimulation for 0.5 h (**C**) or 6 h (**D**). *n* = 3 biological replicates for ELISA. (**E**–**H**) DAI (**E**), colon lengths (**F**), histological scores (**G**), and representative H&E staining images (**H**) of distal colon from WT or *Radx*^−/−^ mice in DSS-induced acute colitis model with or without RI-1 treatment of two independent experiments. Each data point represents one biologically independent individual. (**I**, **J**) Representative immunofluorescence images (**I**) and quantification (**J**) of distal colon from WT or *Radx*^−/−^ mice in DSS-induced acute colitis model with or without RI-1 treatment of two independent experiments. *n* = 8 for WT-Ctrl group, *n* = 9 for WT-DSS-vehicle group, *n* = 6 for WT-DSS with RI-I treatment group, *n* = 7 for *Radx*^−/−^-Ctrl group, *n* = 7 for *Radx*^−/−^-DSS-vehicle group, and *n* = 10 for *Radx*^−/−^-DSS with RI-I treatment group. Each data point represents one biologically independent individual. (**K**) Schematic showing the role of RADX in macrophages upon genotoxin stress. Data were presented as mean ± SEM and analyzed using one-way (**D**, **F**, **J**) ANOVA followed by Tukey–Kramer multiple-comparisons test, or two-tailed Mann–Whitney *U* test (**B**, **G**). Immunoblotting assays was repeated at least three times with independent biological replicates. The exact *P* values are indicated in the figures. Source data are available online for this figure.

Since RADX maintains genomic stability by restraining excessive RAD51 binding at replication forks (Dungrawala et al, 2017), we hypothesized that pharmacologically inhibiting RAD51 could ameliorates DNA damage and inflammatory signaling. We treated cells with RI-1, a small molecule inhibitor that binds to RAD51 and disrupt its activity (Budke et al, 2012). Comet assay results indicated that RI-1 treatment reduced DNA damage not only in RADX-deficiency THP-1 cells, but also in LPS-stimulated control cells (Fig. 6A,B). Accordingly, RI-1 treatment decreased p65 phosphorylation and suppressed inflammatory cytokines secretion in both control and RADX-deficiency macrophages (Fig. 6C,D).

To evaluate the therapeutic potential of RI-1 in vivo, we administered RI-1 (10 mg/kg/day, i.p.) to mice with DSS-induced colitis. RI-1 treatment improved disease outcomes in both WT and *Radx*^−/−^ mice, as evidenced by lower DAI, longer colon lengths, reduced histological scores, enhanced mucus production, and decreased levels of inflammatory cytokines and DNA damage marker (Fig. 6E–J).

Collectively, pharmacological inhibition of RAD51 by RI-1 effectively mitigates DNA damage, dampens inflammatory signaling, and ameliorates experimental colitis.

### DNA damage correlates with inflammation in PIBD patients

**Figure EV7:**
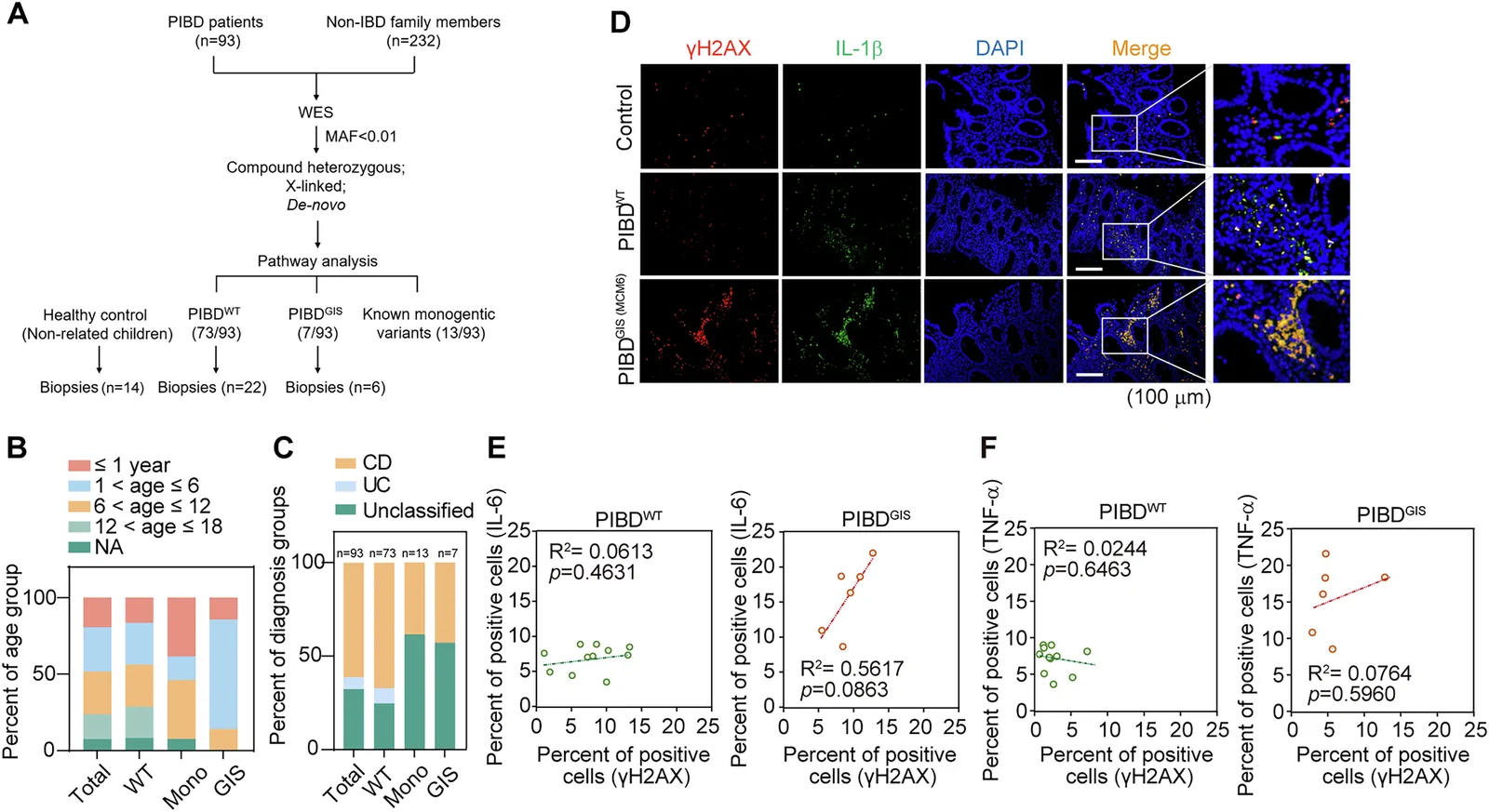
Characteristics of PIBD cohort, genetic instability and inflammation. (**A**) Schematic for patient enrollment. Non-IBD family members and affected PIBD patients were enrolled for whole-exome sequencing (WES) analysis. Compound heterozygous, X-linked recessive, and de novo mutations with minor allele frequency less than 0.01 from the patients (*n* = 93) was selected as candidate risk or disease causal genes for pathway analysis. Patients were stratified according to having known monogenic variants (13/93), variants that cause genomic instability (PIBD^GIS^, 7/93), and other variants (PIBD^WT^, 70/93). Colonic biopsies from non-related healthy control children (*n* = 14), PIBD^GIS^ (*n* = 6) and PIBD^WT^ (*n* = 22) were selected for genomic stability and inflammation study. Biopsy selection was based on material availability. (**B**, **C**) Age range and distribution (**B**) and IBD subtypes (**C**) of the PIBD cohort. (**D**) Representative immunofluorescence images of colon sections from a control subject, a PIBD^GIS^ subject (biopsy from MCM6 mutated patient), and a PIBD^WT^ subject. (**E**, **F**) Correlation analysis of γH2AX with indicated cytokines in PIBD^WT^ and PIBD^GIS^ subjects. The exact *P* values are indicated in the figures.

Although RADX variants themselves are rare, our findings prompted us to investigate whether genomic instability more broadly defines a pathogenic subgroup in PIBD. Thus, we recruited a PIBD cohort comprising 93 patients and 232 family members and performed whole-exome sequencing (WES) (Fig. EV7A; Table EV1).

**Figure 7:**
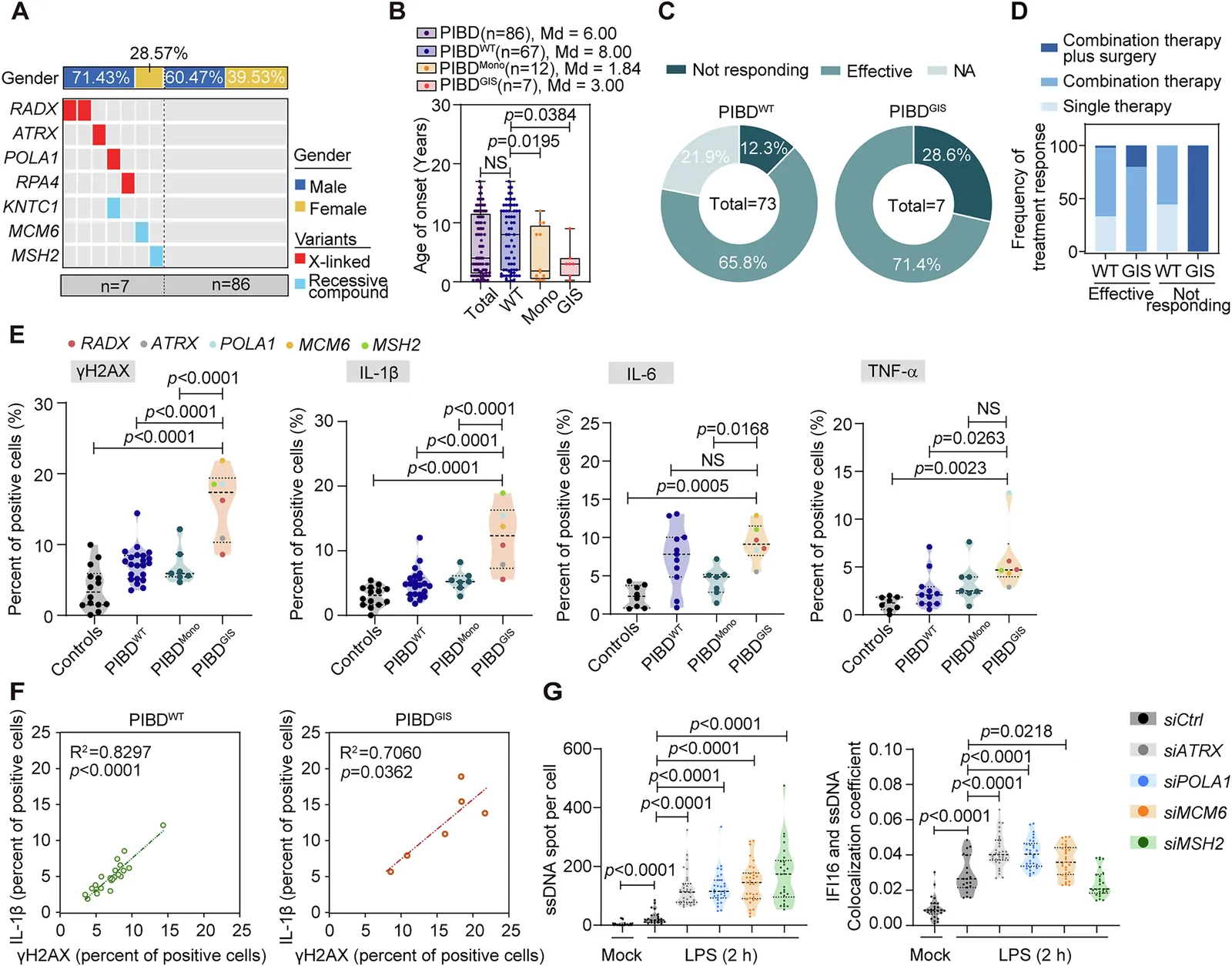
Genomic instability is positively correlated with colonic inflammation in patients with PIBD. (**A**) Whole-exome sequencing (WES) analysis of PIBD patients and their family members (*n* = 93 patients, 232 family members) identified rare mutations in 7 genes from 7 patients that participate in genomic stability maintenance. (**B**) Age of IBD onset of the cohort. Md: median. *n* = 86 for total PIBD group, *n* = 67 for PIBD^WT^ group, *n* = 12 for PIBD^Mono^ group, and *n* = 7 for PIBD^GIS^ group. Each data point represents one biologically independent individual. (**C**, **D**) Response to treatment of PIBD^GIS^ and PIBD^WT^ patients. PIBD^GIS^, *n* = 7. PIBD^WT^, *n* = 73. NA, not available. Not responding refers to fail to response to treatment. Effective refers to clinical remission. Combinational therapy refers to combined use of glucocorticoid with immunosuppressive drugs, mesalazine, and/or biologics. (**E**) Immunofluorescence analysis of inflammatory cytokines expression in colonic sections of healthy donors (Controls, *n* = 7–14), patients with known monogenetic genes mutations (PIBD^Mono^, *n* = 7), patients with identified mutations that affect genomic stability (PIBD^GIS^, *n* = 6) and the rest of PIBD patients (PIBD^WT^, *n* = 22 for γH2AX and IL-1β, *n* = 11 for IL-6 and TNF-α). Each data point represents one biologically independent individual. (**F**) Correlation analysis of γH2AX and IL-1β in the group PIBD^WT^ and PIBD^GIS^ patients. (**G**) THP-1-derived macrophages were transfected with *siCtrl*, *siATRX*, *siPOLA1*, *siMCM6* or *siMSH2*, pre-labelled with BrdU for 48 h and stimulated with LPS for 2 h. *n* = 26 for *siCtrl*-Mock group, *n* = 22 for *siCtrl*-LPS group, *n* = 30 for *siATRX*-LPS group, *n* = 30 for *siPOLA1*-LPS group, *n* = 33 for *siMCM6*-LPS group, and *n* = 26 for *siMSH2*-LPS group. Each data point represents one cell. Data were presented as mean ± SEM and analyzed using one-way ANOVA followed by Tukey–Kramer multiple-comparisons test (**E**, **G**, right) or two-tailed Mann–Whitney *U* test (**B**, **G**, left). In box plot, the centre line represents the median, the box boundaries represent the 25th and 75th percentiles, the whiskers extend from the minimum to the maximum values, and all individual data points are shown. The exact *P* values are indicated in the figures. Source data are available online for this figure.

To identify rare germline variants that may induce genomic instability, we compiled a curated list of genes predicated to be involved in genomic stability maintenance (Dataset EV1). Using this list, we identified putative pathogenic variants in 7 genes across 7 patients (7.5% of the cohort) and designated them as the PIBD^GIS^ subgroup (Fig. 7A). These patients are hypothesized to exhibit elevated DNA damage signaling, representing a “genomic instability signature” (GIS) subgroup. Patients with known monogenic causes of IBD (e.g., *XIAP*, *FOXP3*, *CYBB*, and *IL10RB*) were classified as the PIBD^Mono^ subgroup. The subgroup most commonly occurs in children <6 years of age and accounts for 10–20% of very-early-onset IBD (VEO-IBD) cases (Konys et al, 2025; Ouahed et al, 2020). These monogenic mutations disrupt immunologic cascades and lead to defects in cell signaling, mucosal barrier integrity, and inflammation regulation (Ouahed, 2022). The median age at PIBD onset in the entire cohort was 6 years; however, both PIBD^Mono^ and PIBD^GIS^ patients were diagnosed at significantly younger ages than the remaining patients (designated PIBD^WT^) (Figs. 7B and  EV7B).

CD and unclassified IBD were the most common diagnoses (Fig. EV7C). Clinically, PIBD^GIS^ patients were more likely to be therapy-refractory, require surgical intervention, and receive combination therapies compared to PIBD^WT^ patients, suggesting that genomic instability may be associated with refractory IBD (Fig. 7C,D).

We next assessed the DNA damage and inflammatory signaling in available colonic biopsies. Levels of γH2AX, IL-1β were significantly increased in PIBD^GIS^ subjects compared with PIBD^WT^, PIBD^Mono^, or non-IBD healthy controls (Figs. 7E and  EV7D). A positive correlation was observed between γH2AX signaling and IL-1β levels, independent of the patients’ genetic backgrounds (Fig. 7F). In contrast, no significant association was detected between γH2AX signaling and the expression of IL-6, TNF-α (Fig. EV7E,F). Furthermore, BrdU staining in THP-1-derived macrophages with knockdown of the identified PIBD^GIS^ genes revealed increased ssDNA foci formation and IFI16-ssDNA binding, reinforcing the role of ssDNA accumulation and IFI16 signaling in driving inflammation related to genomic instability (Fig. 7G).

Taken together, these data highlight genomic instability as a risk factor in the pathogenesis of IBD.

## Discussion

The pathogenesis of inflammatory bowel disease (IBD) involves a complex interplay of genetic susceptibility, environmental factors, gut microbiota, and immune dysregulation (Huang et al, 2019; Mitsialis et al, 2020; Ng et al, 2018; Plichta et al, 2019; Uniken Venema et al, 2017). The advent of next-generation sequencing has accelerated the discovery of novel genetic determinants, particularly in pediatric-onset IBD (PIBD) (Leung and Muise, 2018). These implicated genes are broadly categorized into those causing primary immunodeficiency and those disrupting the intestinal epithelial barrier (Leung and Muise, 2018). In this study, we identified a role for RADX in intestinal health by mitigating DNA damage and inhibiting IFI16-mediated DNA sensing. We demonstrated that *Radx* deficiency promotes ssDNA accumulation, hyperinflammation, and aggravated colitis. Moreover, these phenotypes were mitigated by treatment with RI-1, a RAD51 inhibitor (Fig. 6K). Collectively, our data identify that RADX-IFI16 axis as an important contributor to colitis development.

Given the role of RADX in constraining excessive RAD51 activity to preserve genomic stability, we used RI-1 to inhibit RAD51 activity in vitro and in vivo. Intriguingly, RI-1 treatment not only suppressed inflammatory signaling in RADX deficiency macrophages, but also in control cells. Furthermore, it ameliorated colitis in both *Radx*^−/−^ mice and WT mice. We propose two possible hypotheses. First, DNA damage and the inflammatory condition form a vicious cycle, which RI-1 may help to break. Second, RAD51 itself may play an uncovered role in regulating inflammatory pathway, a possibility warranting future investigation.

Two RADX variants (I777T and G829S, respectively) identified in CD patients provide clinical support for our findings. Patient-derived PBMCs and colonic biopsies exhibited reduced RADX protein, elevated DNA damage, as well as hyperinflammatory signaling. However, a larger sample size is needed to strengthen the clinical relevance. Although RADX variants appear rare, our expanded cohort analysis suggests that genomic instability is a hallmark of a distinct subgroup (~ 7.5%) of PIBD patients. While DNA damage is known to activate NF-κB pathway (Dunphy et al, 2018), our data importantly demonstrate that inflammation itself can also induce DNA damage (Fig. 6A,B), thereby establishing a feed-forward loop that amplifies and perpetuates disease. These findings highlight the significance of genomic instability in IBD development and indicate the DNA damage response as a potential therapeutic target.

In summary, our study demonstrates that *Radx* deficiency leads to increased DNA damage, amplifies IFI16-dependent NF-κB signaling and inflammasome activation, thereby contributing to colitis development. The efficacy of RAD51 inhibition in alleviating this pathology underscores the therapeutic potential of targeting this axis. Although additional pathways may also modulate disease, our findings identify the RADX‑IFI16 axis as an important contributor in macrophages. Collectively, this work provides a new perspective on IBD pathogenesis and therapeutic exploration.

## Methods

**Table:** Reagents and tools table

Reagent/resource	Reference or source	Identifier or catalog number
**Experimental models**		
*Radx*^−/−^ mice and littermates	Cyagen Biosciences Inc.	N/A
THP-1 cells	ATCC	TIB-202
HEK 293 T cells	ATCC	CRL-11268
PBMCs	Guangzhou Women and Children’s Medical Center	N/A
Colonic biopsies	Guangzhou Women and Children’s Medical Center, Six Affiliated Hospital, Sun Yat-sen University	N/A
**Recombinant DNA**		
50-mer poly(dT)	This study	(5’-Dual Biotin) TTTTTTTTTTTTTTTTTTTTTTTTTTTTTTTTTTTTTTTTTTTTTTTTTT
**Antibodies**		
Phospho-Histone H2A.X (Ser139) (20E3) Rabbit mAb	Cell Signaling Technology	9718S, 1:1000 for immunoblot and 1:200 for IF staining
Phospho-NF-κB p65 (Ser536) Antibody	Cell Signaling Technology	3031S, 1:1000 for immunoblot
NF-κB p65 (C22B4) Rabbit mAb	Cell Signaling Technology	4764S, 1:1000 for immunoblot
IL-1β (3A6) Mouse mAb	Cell Signaling Technology	12242S, 1:1000 for immunoblot
NDP52 (D1E4A) Rabbit mAb	Cell Signaling Technology	60732S, 1:1000 for immunoblot
Phospho-IKKα/β (Ser176/180) Rabbit mAb	Cell Signaling Technology	2697S, 1:1000 for immunoblot
IKKα Antibody	Cell Signaling Technology	2682S, 1:1000 for immunoblot
IKKβ (L570) Antibody	Cell Signaling Technology	2678S, 1:1000 for immunoblot
Phospho-p38 MAPK (Thr180/Tyr182) Antibody	Cell Signaling Technology	9211S, 1:1000 for immunoblot
p38 MAPK Antibody	Cell Signaling Technology	9212S, 1:1000 for immunoblot
Phospho-SAPK/JNK (Thr183/Tyr185) Antibody	Cell Signaling Technology	9251S, 1:1000 for immunoblot
SAPK/JNK Antibody	Cell Signaling Technology	9252S, 1:1000 for immunoblot
cGAS (D1D3G) Rabbit mAb	Cell Signaling Technology	15102S, 1:1000 for immunoblot
ATM (11G12) Mouse mAb	Cell Signaling Technology	92356S, 1:1000 for immunoblot
IFI16 (D8B5T) Rabbit mAb	Cell Signaling Technology	14970S, 1:1000 for immunoblot and 1:200 for IF staining
STING (D2P2F) Rabbit mAb	Cell Signaling Technology	13647S, 1:1000 for immunoblot and 1:200 for IF staining
TRAF6	Cell Signaling Technology	8028S, 1:1000 for immunoblot
BrdU (Bu20a) Mouse mAb	Cell Signaling Technology	5292S, 1:200 for IF staining
CXorf57 antibody	Biorbyt	Orb258384, 1:1000 for immunoblot and 1:200 for IF staining
Anti-rabbit IgG, HRP-linked Antibody	Cell Signaling Technology	7074S, 1:2000 for immunoblot
Anti-mouse IgG, HRP-linked Antibody	Cell Signaling Technology	7076S, 1:2000 for immunoblot
Phospho-IRF-3 (Ser396) (D6O1M) Rabbit mAb	Cell Signaling Technology	29047S, 1:1000 for immunoblot
IRF-3 (D83B9) Rabbit mAb	Cell Signaling Technology	4302S, 1:1000 for immunoblot
DYKDDDDK Tag (9A3) Mouse mAb	Cell Signaling Technology	8146S, 1:1000 for immunoblot
HA-Tag (6E2) Mouse mAb	Cell Signaling Technology	2999S, 1:1000 for immunoblot
Myc-Tag (9B11) Mouse mAb	Cell Signaling Technology	2040S, 1:1000 for immunoblot
β-Actin (13E5) Rabbit mAb	Cell Signaling Technology	5125S, 1:5000 for immunoblot
Goat anti-Rabbit IgG (H + L) Cross-Adsorbed Secondary Antibody, Alexa Fluor 594	ThermoFisher	A-11012, 1:500 for IF staining
Goat anti-Mouse IgG (H + L) Highly Cross-Adsorbed Secondary Antibody, Alexa Fluor 488	ThermoFisher	A-11029, 1:500 for IF staining
IL-6 Antibody	Santa Cruz Biotechnology	sc-28343, 1:200 for IF staining
TNF Alpha Antibody	Proteintech	60291-1, 1:200 for IF staining
MX1 Rabbit Polyclonal Antibody	Proteintech	13750-1-AP, 1:100 for IF staining
BST2 Rabbit Polyclonal Antibody	Proteintech	13560-1-AP, 1:200 for IF staining
Lamin A/C Polyclonal antibody	Proteintech	10298-1-AP, 1:1000 for immunoblot
Alpha Tubulin Polyclonal antibody	Proteintech	11224-1-AP, 1:1000 for immunoblot
Anti-CD68 antibody [C68/684]	Abcam	ab201340, 1:200 for IF staining
Anti-MUC2 antibody [EPR23479-47]	Abcam	ab272692, 1:200 for IF staining
Rat monoclonal anti-BrdU Antibody	Abcam	ab6326, 1:400
Mouse monoclonal anti-BrdU Antibody	BD Biosciences	347580, 1:400
CD11c	Abcam	ab275738, 1:200 for IF staining
EpCAM	Abcam	ab71916, 1:200 for IF staining
PDGRFα	Abcam	ab307855, 1:200 for IF staining
F4/80	Cell Signaling Technology	71299S, 1:200 for IF staining
AIM2	Cell Signaling Technology	12948S, 1:1000 for immunoblot
NLRP3	Cell Signaling Technology	15101S, 1:1000 for immunoblot
ASC	Santa Cruz Biotechnology	sc-271054, 1:1000 for immunoblot and 1:200 for IF staining
ATRX	Abcam	ab97508, 1:1000 for immunoblot
POLA1	Abcam	ab31777, 1:1000 for immunoblot
MCM6	Abcam	ab201683, 1:1000 for immunoblot
MSH2	Abcam	ab212188, 1:1000 for immunoblot
**Oligonucleotides and other sequence-based reagents**		
*siRADX*-Sense	This study	GCUUGAACUCUCUCGUAUATT
*siRADX*-Anti-sense	This study	UAUACGAGAGAGUUCAAGCTT
*si-CGAS*-Sense	This study	GGCUAUCCUUCUCUCACAUTT
*si-CGAS*-Anti-sense	This study	AUGUGAGAGAAGGAUAGCCTT
*si-IFI16*-Sense	This study	GCUUUGCUCACAAACUAAATT
*si-IFI16*-Anti-sense	This study	UUUAGUUUGUGAGCAAAGCTT
*si-ATM*-Sense	This study	GCUAUUUACGGAGCUGAUUTT
*si-ATM*-Anti-sense	This study	AAUCAGCUCCGUAAAUAGCTT
*si-STING*-Sense	This study	GCAUUACAACAACCUGCUATT
*si-STING*-Anti-sense	This study	UAGCAGGUUGUUGUAAUGCTT
*si-TRAF6*-Sense	This study	GGUGAAAUGUCCAAAUGAADTDT
*si-TRAF6*-Anti-sense	This study	UUCAUUUGGACAUUUCACCDTDT
*si-ATRX*-Sense	This study	CCAAAGAAGACUAGUUCAA
*si-ATRX*-Anti-sense	This study	UUGAACUAGUCUUCUUUGG
*si-POLA1*-Sense	This study	GCCAGAUCCAAGCUUAGAA
*si-POLA1*-Anti-sense	This study	UUCUAAGCUUGGAUCUGGC
*si-MCM6*-Sense	This study	GAUUCUUACUGGAUACAAA
*si-MCM6*-Anti-sense	This study	UUUGUAUCCAGUAAGAAUC
*si-MSH2*-Sense	This study	CUUGUGUUGAAGUUCAAGA
*si-MSH2*-Anti-sense	This study	UCUUGAACUUCAACACAAG
*si-BRCA2*-1-Sense	This study	CACCCAGUACAACAUUCAA
*si-BRCA2*-1-Anti-sense	This study	UUGAAUGUUGUACUGGGUG
*si-BRCA2*-2-Sense	This study	GUUCCCAAUAGUAGACAUA
*si-BRCA2*-2-Anti-sense	This study	UAUGUCUACUAUUGGGAAC
*hTNF*-Forward	This study	CCAGACCAAGGTCAACCTCC
*hTNF*-Reverse	This study	CAGACTCGGCAAAGTCGAGA
*hIL1B*-Forward	This study	ATGATGGCTTATTACAGTGGCAA
*hIL1B*-Reverse	This study	GTCGGAGATTCGTAGCTGGA
*hBRCA2*-Forward	This study	CACCCACCCTTAGTTCTACTGT
*hBRCA2*-Reverse	This study	CCAATGTGGTCTTTGCAGCTAT
*hRADX*-Forward	This study	AGGGCTAGATTGGCCGAAC
*hRADX*-Reverse	This study	CAGCACAGTTACAGGCACC
*hMUC2*-Forward	This study	GGAGATCACCAATGACTGCGA
*hMUC2*-Reverse	This study	GAATCGTTGTGGTCACCCTTG
*hOCLN*-Forward	This study	GACTTCAGGCAGCCTCGTTAC
*hOCLN*-Reverse	This study	GCCAGTTGTGTAGTCTGTCTCA
*hCLDN3*-Forward	This study	AACACCATTATCCGGGACTTCT
*hCLDN3*-Reverse	This study	GCGGAGTAGACGACCTTGG
*hRPL13A*-Forward	This study	GCCATCGTGGCTAAACAGGTA
*hRPL13A*-Reverse	This study	GTTGGTGTTCATCCGCTTGC
*hTERT*-Forward	This study	TCACGGAGACCACGTTTCAAA
*hTERT*- Reverse	This study	TTCAAGTGCTGTCTGATTCCAAT
*mTnf-*Forward	This study	CCTGTAGCCCACGTCGTAG
*mTnf*-Reverse	This study	GGGAGTAGACAAGGTACAACCC
*mIl1*-Forward	This study	GAAATGCCACCTTTTGACAGTG
*mIl1*-Reverse	This study	TGGATGCTCTCATCAGGACAG
*mIl6*-Forward	This study	CTGCAAGAGACTTCCATCCAG
*mIl6*-Reverse	This study	AGTGGTATAGACAGGTCTGTTGG
*mGapdh*-Forward	This study	TGGCCTTCCGTGTTCCTAC
*mGapdh*-Reverse	This study	GAGTTGCTGTTGAAGTCGCA
**Chemicals, enzymes and other reagents**		
Vectashield Antifade Mounting Medium with DAPI	Vector	H-1200
Lipopolysaccharides	Sigma	L2880
Etoposide	Sigma	E1383
Cycloheximide (CHX)	Sigma	C1988
Z-leu-leu-leu-al (MG132)	Sigma	C2211
Chloroquine phosphate (CQ)	Sigma	PHR1258
3-Methyladenine (3MA)	Sigma	M9281
Bafilomycin A1 (Baf A1)	Selleck	S1413
TRIzol™	ThermoFisher	15596018
Dextran Sulfate Sodium (DSS)	MP Biomedicals	160110
RI-1	MedChemExpress	HY-15317
3x FLAG Peptide	APExBIO	A6001
Lipofectamine RNAiMAX	ThermoFisher	13778150
Lipofectamine 3000 Transfection	ThermoFisher	L3000015
Dynabeads™ MyOne™ Streptavidin T1	ThermoFisher	65601
BrdU (5-Bromo-2´-Deoxyuridine)	ThermoFisher	B23151
RPMI 1640 Medium	ThermoFisher	61870036
Dulbecco’s Modified Eagle Medium	ThermoFisher	11995065
Fetal Bovine Serum	ThermoFisher	16140071
Protein A Agarose	ThermoFisher	20333
Protein G Agarose	ThermoFisher	20398
ANTI-FLAG® M2 Affinity Gel	Sigma	A2220
2,4,6-Trinitro-benzene sulfonic acid (TNBS)	Sigma	P2297
5-Iodo-20-deoxyuridine (IdU)	Sigma	I7125
5-Chloro-20-deoxyuridine (CldU)	Sigma	C6891
Propidium Iodide	ThermoFisher	P3566
FITC-Dextran (mol wt 4000)	Sigma	46944
Recombinant Human TNF-α	PeproTech	PeproTech
**Critical commercial assays**		
IL-1 beta Human Uncoated ELISA Kit	ThermoFisher	88-7261-88
IL-6 Human Uncoated ELISA Kit	ThermoFisher	88-7066-88
TNF alpha Human Uncoated ELISA Kit	ThermoFisher	88-7346-88
IL-1 beta Mouse Uncoated ELISA Kit	ThermoFisher	88-7013-88
IL-6 Mouse Uncoated ELISA Kit	ThermoFisher	88-7064-88
TNF alpha Mouse Uncoated ELISA Kit	ThermoFisher	88-7324-88
Mouse NGAL (Neutrophil Gelatinase Associated Lipocalin) ELISA Kit	Finetest	EM20000
HiScript III RT SuperMix for qPCR	Vazyme	R323-01
ChamQ Universal SYBR qPCR Master Mix	Vazyme	Q711-02
Comet Assay Kit	Abcam	ab238544
Proximity Ligation Assay reagent	Sigma	DUO92008
Duolink In Situ PLA Probe Anti-Mouse MINUS	Sigma	DUO92004
Duolink In Situ PLA Probe Anti-Rabbit PLUS	Sigma	DUO92002
**Software**		
ImageJ	NIH	N/A
Image Lab	BIO-RAD	N/A
Prism 6	GraphPad	N/A
Casp_1.2.3b1	CaspLab	N/A
LAS X 3.0.11	Leica Microsystems	N/A
BioRender	© BioRender 2022	N/A
PyMOL2.2	PyMOL by Schrödinger	N/A
**Other**		
TIRF + THUNDER Leica High-Resolution Microscope	Leica Microsystems	N/A
Applied Biosystems QuantStudio 6 Flex	ThermoFisher	N/A
Varioskan LUX	ThermoFisher	N/A
Pannoramic MIDI II Digital Scaner	3DHISTECH	N/A
ChemiDoc XRS+	Bio-Rad	N/A

### Study design

The aim of this study was to investigate the role of RADX in colitis. We generated *Radx* knockout mice and assessed the phenotype of *Radx*^−/−^ mice in both DSS-induced and TNBS-induced acute colitis models. BMT was performed to exclude the contribution of hematopoietic versus non-hematopoietic cells. Two variants, I777T and G829S were identified in two CD patients, respectively. Given that RADX maintains genomic stability by suppressing RAD51, we investigated whether the RAD51 inhibitor RI-1 could counteract the hyperinflammatory signaling caused by RADX deficiency in vivo. Furthermore, we recruited a PIBD cohort and performed WES to determine the proportion of genomic instability in this population.

### Ethical statement

The Medical Ethics Committee of Guangzhou Women and Children’s Medical Center (2021020B01) and Six Affiliated Hospital, Sun Yat-sen University (2022ZSLYEC-316) approved the study procedures for human subjects. Implementations followed the International Ethical Guidelines for Research Involving Human Subjects as stated in the Helsinki Declaration. The legal guardians of all participants signed the consent forms. The experiments also conformed to the principles set out in the Department of Health and Human Services Belmont Report.

### Cohort recruitment

A cohort of children (under 18 years of age) who attended the Gastroenterology Department were recruited. All patients underwent ileocolonoscopy examination for unexplained abdominal pain, bloating, persistent diarrhea, blood in the stool, anemia, hypoproteinemia or failure to thrive. Bacterial, viral and parasitic infections, as well as tuberculosis and malignancies were excluded for all patients. IBD, including UC, CD, and undifferentiated IBD were diagnosed based on a combination of history, physical and laboratory examination, esophagogastroduodenoscopy (EGD), ileocolonoscopy and small bowel imaging according to Porto Criteria (Levine et al, 2014). Healthy control subjects were those with no colonoscopy and histopathologic features of inflammation.

### Mice

We developed a *Radx* deficient mouse line (*Radx*^−/−^) on C57BL/6 background by CRISPR-Cas9 mediated genomic editing. The Institutional Animal Care and Use Committee of Guangzhou Forevergen Biosciences Medical Laboratory Animal Center approved the animal studies (IACUC-AEWC-F20112023). *Radx* knockout mouse was designed by Cyagen Biosciences Inc. (Shanghai, China) and bred by Forevergen Biosciences Centre (Guangzhou, China).

All animal procedures were conducted in accordance with the ARRIVE guidelines. For all experiments, 6 to 8-week-old male mice were used. Animals were maintained under specific pathogen-free (SPF) conditions at 22 ± 2 °C with a 12-h light/dark cycle, and had free access to food and water. Mice were housed at a maximum of five per cage. Wild-type littermates were used as controls. The animal facility was routinely monitored for pathogens, and all health reports were negative during the experimental period.

For DSS-induced acute colitis model, mice were administered 3% DSS (36–50 kDa; MP Biomedicals) in their drinking water ad libitum for 7 consecutive days, followed by 2 days of normal drinking water. Control mice were administered with normal drinking water throughout the duration of the experiment. Weight loss, rectal bleeding and diarrhea were monitored daily at the same time. The DSS water was replaced on days 3 and 5. Mice were sacrificed on day 9. Fecal samples were collected and immediately placed on the dry ice for subsequent LCN2 detection. After photographing the whole colon, rings of distal and proximal colon were fixed in 4% paraformaldehyde for subsequent H&E and immunofluorescence staining. The remaining colon tissues were collected in RNAlater (Invitrogen, AM7021) for the RNA extraction and real-time PCR analysis.

TNBS/ethanol-induced acute colitis model was generated as described previously (Wirtz et al, 2017). Briefly, on day 1, 6–8 weeks old male mice were weighed and anesthetized with isoflurane-mixed gas. Mice were shaved with an electric razor for a 1.5 × 1.5-cm area of the skin on the back between the shoulders, and 150 μL of 1% (wt./vol) TNBS pre-sensitization solution was applied to the shaved skin area with a pipette. On day 8, mice were weighed, anesthetized, and positioned on isothermal warming pads. In total, 100 μl of 2.5% TNBS solution (wt./vol) was gently infused into the colon through the anus with a 3.5 F catheter. After removal of the catheter from the colon, the mice were positioned head down for 5 min.

In the DSS-induced chronic colitis, mice received 2% DSS (36–50 kDa; MP Biomedicals) in drinking water ad libitum for 7 consecutive days (day 0–7), followed by 2 weeks of normal drinking water (days 8–21). DSS and water administration cycle was repeated 3 times (Wirtz et al, 2017). Control mice were given normal drinking water throughout the experiment. Body weight, rectal bleeding, and diarrhea were monitored daily at the same time. DSS-containing water was replaced on days 3 and 5 of each DSS cycle.

Both the research team and the veterinary staff monitored animals every day. Health was monitored by weight, food and water intake, and general assessment of animal activity. We scored inflammation-associated histological changes in the colon according to previous description (Wirtz et al, 2017).

**Disease activity index (DAI) criteria** **(Wirtz et al,** 2017**)**

**Table:** 

Score	Weight loss (%)	Stool consistency	Blood in stool
0	0	Normal	Negative hemocult
1	1–5	Soft but still formed	Negative hemocult
2	6–10	Soft	Positive hemocult
3	11–18	Very soft; wet	Blood traces in stool visible
4	>18	Watery diarrhea	Gross rectal bleeding

**Scoring system for DSS-induced histological changes in the colon** **(Wirtz et al,** 2017**)**

**Table:** 

Score	Tissue damage	Lamina propria inflammatory cell infiltration
0	None	Infrequent
1	Isolated focal epithelial damage	Increased, some neutrophils
2	Mucosal erosions and ulcerations	Submucosal presence of inflammatory cell clusters
3	Extensive damage deep into the bowel wall	Transmural cell infiltrations

**Scoring system for TNBS-induced histological changes in the colon** **(Wirtz et al,** 2017**)**

**Table:** 

Score	Histologic changes in TNBS colitis
0	No evidence of inflammation
1	Low level of inflammation, with scattered infiltrating mononuclear cells (1–2 foci)
2	Moderate inflammation, with multiple foci
3	High level of inflammation, with increased vascular density and marked wall thickening
4	Maximal severity of inflammation, with transmural leukocyte infiltration and loss of goblet cells

### Bone marrow transplantation

*Radx*^*−/−*^ mice and WT littermates (males, 8 weeks old) were used as donors of bone marrow cells. Bone marrow was isolated from the femurs of hind legs. Following red blood cell lysis, the cell suspension was strained through a 70 μm cell strainer. The cell pellets were washed three times with sterile PBS, counted, and stored on ice for subsequent injection. Recipient *Radx*^*−/−*^ mice and WT littermates (males, 8 weeks old) were lethally irradiated with a total dose of 8.0 Gy (1.0 Gy/min) and transplanted with 1 × 10^7^ bone marrow cells via ophthalmic vein injection. Mice were housed in specific-pathogen free facilities and provided with drinking water supplemented with gentamicin (1 mg/mL) for 7 days. Thereafter, they received sterilized water and standard chow ad libitum for 6 weeks to reconstitute the immune system before being subjected to the colitis model.

### Cell culture and transfection

HEK 293T (human embryonic kidney 293T) and THP-1 cells were purchased from ATCC. HEK 293T and THP-1 cells were maintained in DMEM (Hyclone) or RPMI-1640 medium (Gibco) supplemented with 10% fetal bovine serum (Gibco) and 1% L-glutamine (Gibco) at 37 °C in 5% CO_2_. THP-1 cells were differentiated into macrophages by PMA (100 nM) (InvivoGen) treatment for 18 h. Plasmids were transfected using Lipofectamine 3000 reagent (L3000015, Invitrogen). siRNAs were purchased from Tsingke Biological Technology (Shanghai, China) and were transfected using Lipofectamine RNAiMAX reagent (A13778150, Invitrogen).

### Single strand DNA label by BrdU and immunofluorescence microscopy

THP-1-derived macrophages were plated in Scientific Glass Bottom Cell Culture Dish (801001, NEST) and transfected with siCtrl or *RADX*-specific siRNA. Cells were labeled with 1 mM BrdU (23151, Invitrogen) for 48 h before being washed with PBS for three times and returned to BrdU-free medium. After LPS stimulation for 2 h, cells were fixed with 4% paraformaldehyde for 15 min at 4 °C and then permeabilized in methyl alcohol for 10 min at 4 °C. After washing with PBS, cells were blocked in 5% fetal goat serum for 1 h, and incubated with primary antibodies (1:200) overnight at 4 °C. Cells were washed with PBS and incubated with fluorescently labeled secondary antibodies (1:500). DAPI (5 μg/mL) was used to stain nuclear DNA. Immunofluorescent images were acquired with a Leica TCS SP8 Inverted Fluorescence Microscope (Leica Microsystems).

### Comet assay

THP-1 cells were transfected with indicated siRNA and treatment. The comet assay was performed according to the manufacturer’s instructions (ab238544, Abcam). DNA damage was measured in terms of tail moments with CaspLab.

### In vitro DNA pull-down assay

HEK 293T cells were transfected with plasmids encoding Flag-IFI16 or Flag-RADX for 36 h. Cell lysates were prepared and subjected to immunoprecipitation using anti-Flag magnetic beads with rotation at 4 °C. The beads were washed three times with lysis buffer and then incubated with Flag peptides (5 mg/mL) to elute the bound proteins. After centrifuging at 12,000×*g* for 1 min at 4 °C, the supernatant containing the enriched proteins was collected.

Biotin-DNA pull-down assay was performed as previously described (Dungrawala et al, 2017). Briefly, streptavidin-coupled Dynabeads T1 (LifeTechnologies) were washed twice in TE buffer and incubated with biotinylated DNA substrates at room temperature for 30 min. The beads were washed twice again in TE buffer, followed by two washes in binding buffer (80 mM Tris, pH 7.5, 100 mM KCl, 5 mM MgCl_2_, 2 mM DTT and 100 mg/mL BSA in RNase/DNase free water). One microliter of beads with 4 picomoles of bound DNA was resuspended in binding buffer. The indicated amount of enriched protein was added to the bead suspension and incubated with rotation at room temperature for 30 min. The supernatant was removed, and the beads were boiled in 2× sample buffer for 5 min. Captured proteins were analyzed by immunoblotting. The ssDNA oligo utilized for pull-down assays was a 50-mer poly(dT) (poly-dT_50_).

### In vitro RADX-RAD51 binding assay

HEK293T cells were transfected with Flag-RAD51, Myc-RADX WT, I777T or G829S, respectively. Lysates were enriched with anti-Flag M2 magnetic beads (M8823, Sigma) or anti-c-myc agarose affinity gel (A7470, Millipore) incubation and Flag- (A6001, APExBIO) or Myc-peptide (A6003, APExBIO) processing (5 mg/mL). RADX-RAD51 binding assay was processed as reported previously (Adolph et al, 2021).

### DNA fiber assay

*RADX* KO HEK 293 T cells were transfected with Myc-empty vector (EV), or Myc-RADX-WT, or Myc-RADX-I777T or Myc-RADX-G829S plasmids for 24 h, followed by incubations of prewarmed 50 μM IdU (I7125, Sigma) and 250 μM CldU (C6891, Sigma) for 20 min, sequentially. Cells were trypsinized and resuspended in ice-cold PBS. DNA fiber assay was performed as reported previously (Tian et al, 2021). Images were acquired using ×63 oil immersion objective of Leica DMi8 Inverted Fluorescence Microscopes and analyzed by ImageJ.

### Cytosolic nuclear DNA (nucDNA) assay

Cells were washed with PBS and resuspended with cytosolic extraction buffer (150 mM NaCl, 50 mM HEPES pH 7.4, 25 μg/mL digitonin) and rotated for 10 min at 4 °C. Cell lysates were centrifuged at 800×* g* for 3 min to obtain supernatant excluding nuclear component. Supernatant was then centrifuged for second time at 10,000× *g* for 5 min to remove cell debris. Supernatant was collected for cytosolic DNA extraction by using QIAamp DNA Micro Kit (56304, QIAGEN). Quantitative PCR was performed by using nuclear DNA primers (Tert).

### Mass spectrometry analysis

Endogenous RADX in THP-1-derived macrophages was enriched by co-immunoprecipitation. Samples were ready for mass spectrum analysis by Applied Protein Technology Co., Ltd.

### Statistics

Mice were randomly allocated to experimental groups using block randomization (based on age and weight). Randomization was performed by a researcher not involved in the experiments using a random number generator. To minimize potential observer bias and avoid overestimation of treatment effects, all laboratory members who performed phenotype assessments were blinded to the treatment allocation throughout the experiment and until data acquisition was completed.

No formal power calculation was conducted. Sample sizes were determined based on previous experience and standard practice in the field, ensuring sufficient replicates to detect biologically meaningful differences. Sample sizes for individual experiments are indicated in the corresponding figure legends. For animal experiments, each experimental group contained at least three mice. Sample sizes were determined based on pilot studies and published work using the same models. For in vitro experiments, at least three independent biological replicates were performed.

We used Prism 8.0 (GraphPad Software) for statistical analyses. Shapiro–Wilk normality test was first performed for data distribution. For parametric data, we used one-way or two-way ANOVA followed by Tukey–Kramer multiple-comparisons test. For non-parametric data, we used Mann–Whitney *U* test for two group analysis. All statistical tests were two-tailed. Representative data from at least three biological repeats with consistent findings was shown, and data was expressed as mean ± SEM except otherwise indicated.

## Supplementary information

Table EV1

Peer Review File

Dataset EV1

Source data Fig. 1

Source data Fig. 2

Source data Fig. 3

Source data Fig. 4

Source data Fig. 5

Source data Fig. 6

Source data Fig. 7

Figure EV1 Source Data

Figure EV2 Source Data

Figure EV3 Source Data

Figure EV4 Source Data

Figure EV5 Source Data

Figure EV6 Source Data

Expanded View Figures

## Data Availability

This study includes no data deposited in external repositories. The source data of this paper are collected in the following database record: biostudies:S-SCDT-10_1038-S44321-026-00494-6.
