# Supplementary material for: RADX protects against intestinal inflammation by restraining IFI16-mediated innate immunity
Source: EMBO Mol Med. 2026 Jul 24;18(9):3570–96. doi: 10.1038/s44321-026-00494-6 (PMC13562710; doi:10.1038/s44321-026-00494-6)
Supplement: Supplementary file 1 [file 44321_2026_494_MOESM17_ESM.pdf]

Expanded View Figures

Figure EV1. *Radx*$^{-/-}$ mice show an exacerbated colitis phenotype in DSS-induced chronic colitis model.

(**A**) CRISPR/Cas9 strategy was used to generate the *Radx*$^{-/-}$ mouse line on C57BL/6 background. (**B**) Gel electrophoresis showing representative genotyping data for WT and *Radx*$^{-/-}$ mice. (**C**) Schematic of DSS-induced chronic colitis model. (**D**, **E**) Disease activity index (DAI) (**D**) and colon lengths (**E**) of WT or *Radx*$^{-/-}$ mice in DSS-induced acute colitis model. $n = 4$ in WT-Ctrl group, $n = 6$ in WT-DSS group, $n = 3$ in *Radx*$^{-/-}$-Ctrl group, and $n = 6$ in *Radx*$^{-/-}$-DSS group. Each data point represents one biologically independent individual. (**F–I**) Representative images of hematoxylin-eosin (H&E) staining (**F**), histological scores (**G**), immunofluorescence analysis (**H**), and quantification of immunofluorescence staining (**I**) of distal colon segments from WT or *Radx*$^{-/-}$ mice in DSS-induced chronic colitis model. Each data point represents one biologically independent individual. Data were presented as mean ± SEM and analyzed using one-way (**E**, **G**, **I**) or two-way (**D**) ANOVA followed by Tukey–Kramer multiple-comparisons test. In box plot, the centre line represents the median, the box boundaries represent the 25th and 75th percentiles, the whiskers extend from the minimum to the maximum values, and all individual data points are shown. The exact *P* values are indicated in the figures. Source data are available online for this figure.

© The Author(s)

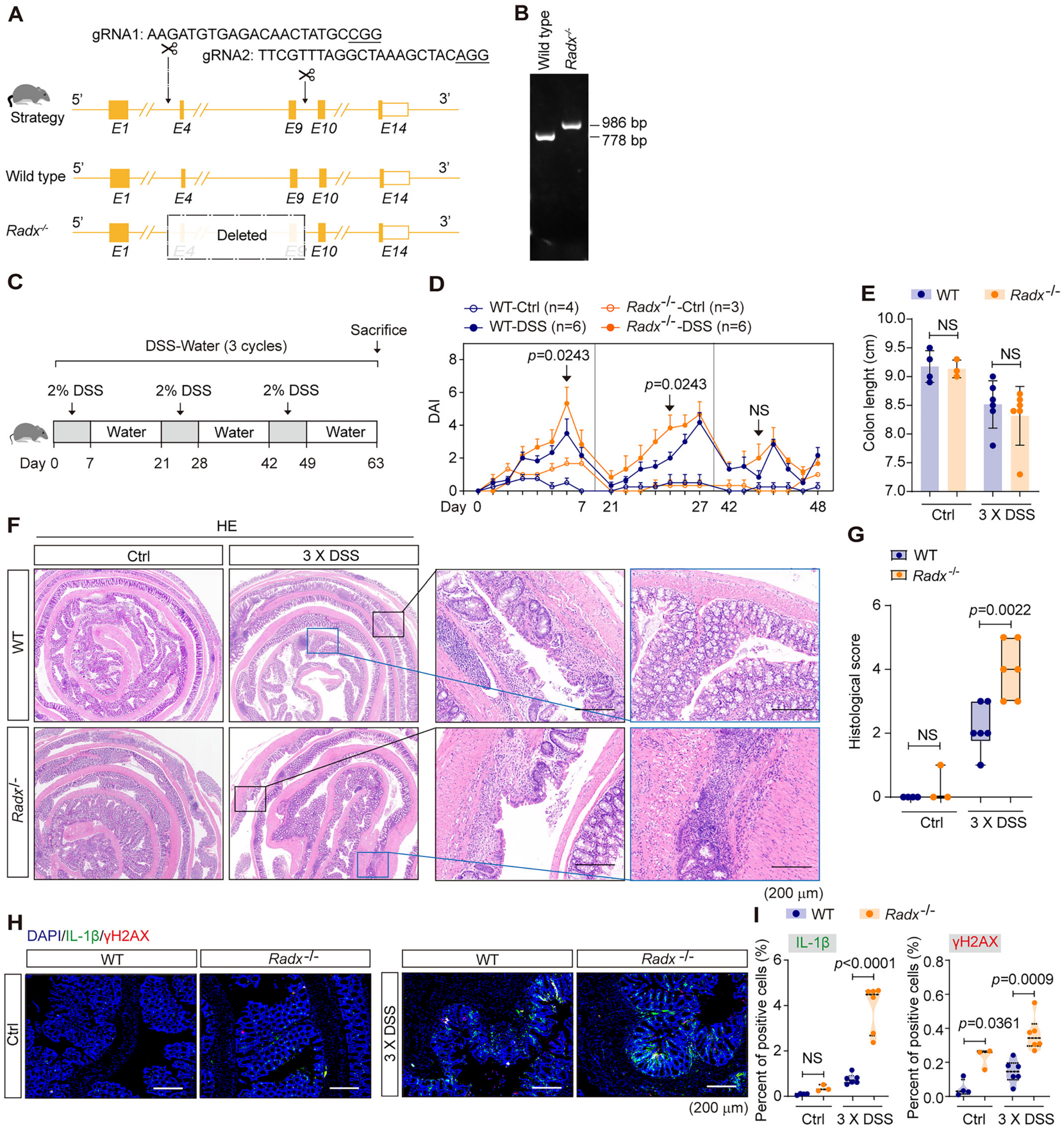
A
gRNA1: AAGATGTGAGACAACTATGCCGG
gRNA2: TTCGTTTAGGCTAAAGCTACAGG
Strategy
Wild type
Radx-/-
Deleted
E1 E4 E9 E10 E14
B
Wild type
Radx-/-
986 bp
778 bp
C
Sacrifice
DSS-Water (3 cycles)
2% DSS
Water
Day 0 7 21 28 42 49 63
D
WT-Ctrl (n=4)
WT-DSS (n=6)
Radx-/--Ctrl (n=3)
Radx-/--DSS (n=6)
p=0.0243
NS
DAI
E
WT
Radx-/-
Colon lenght (cm)
Ctrl
3 X DSS
F
HE
G
Histological score
p=0.0022
(200 μm)
H
DAPI/IL-1β/γH2AX
I
IL-1β
γH2AX
Percent of positive cells (%)
p<0.0001
p=0.0361
p=0.0009

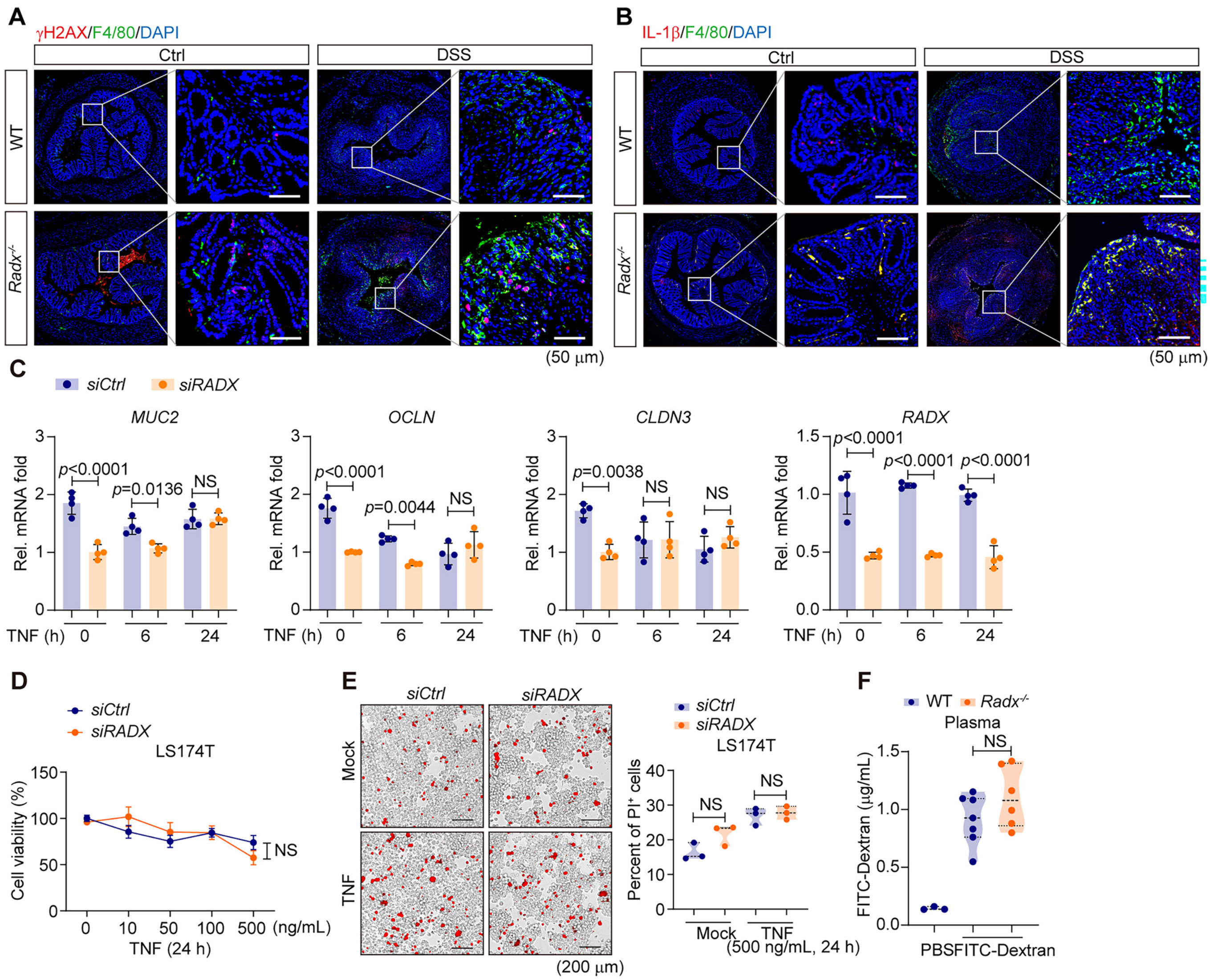

Figure EV2. RADX function in intestinal epithelial cells.

(**A**, **B**) Representative immunofluorescence images of distal colon from WT or *Radx*$^{-/-}$ mice in DSS-induced colitis model. Quantification results were shown in Fig. 2C,D, respectively. (**C**) LS174T cells transfected with *siCtrl* or *siRADX* were treated with TNF (100 ng/mL) for indicated time course. Real-time PCR was conducted to analyze the mRNA expression. Data are from $n = 3$ independent biological replicates. (**D**) LS174T cells transfected with *siCtrl* or *siRADX* were treated with indicated dosage of TNF for 24 h. Cell viability was assessed by CCK-8 assay. Data are from $n = 3$ independent biological replicates. (**E**) LS174T cells transfected with *siCtrl* or *siRADX* were treated with TNF (500 ng/mL) for 24 h. Cell death was assessed by propidium iodide (PI) staining and fluorescence microscopy. Data are from $n = 3$ independent biological replicates. (**F**) 8-week-old mice were administered with FITC-dextran (600 mg/kg) by oral gavage. After 4 h blood samples were collected and plasma FITC fluorescence was measured using a fluorescence microplate reader. Each data point represents one biologically independent individual. Data were presented as mean ± SEM and analyzed using one-way ANOVA followed by Tukey-Kramer multiple-comparisons test. The exact *P* values are indicated in the figures. Source data are available online for this figure.

 © The Author(s)

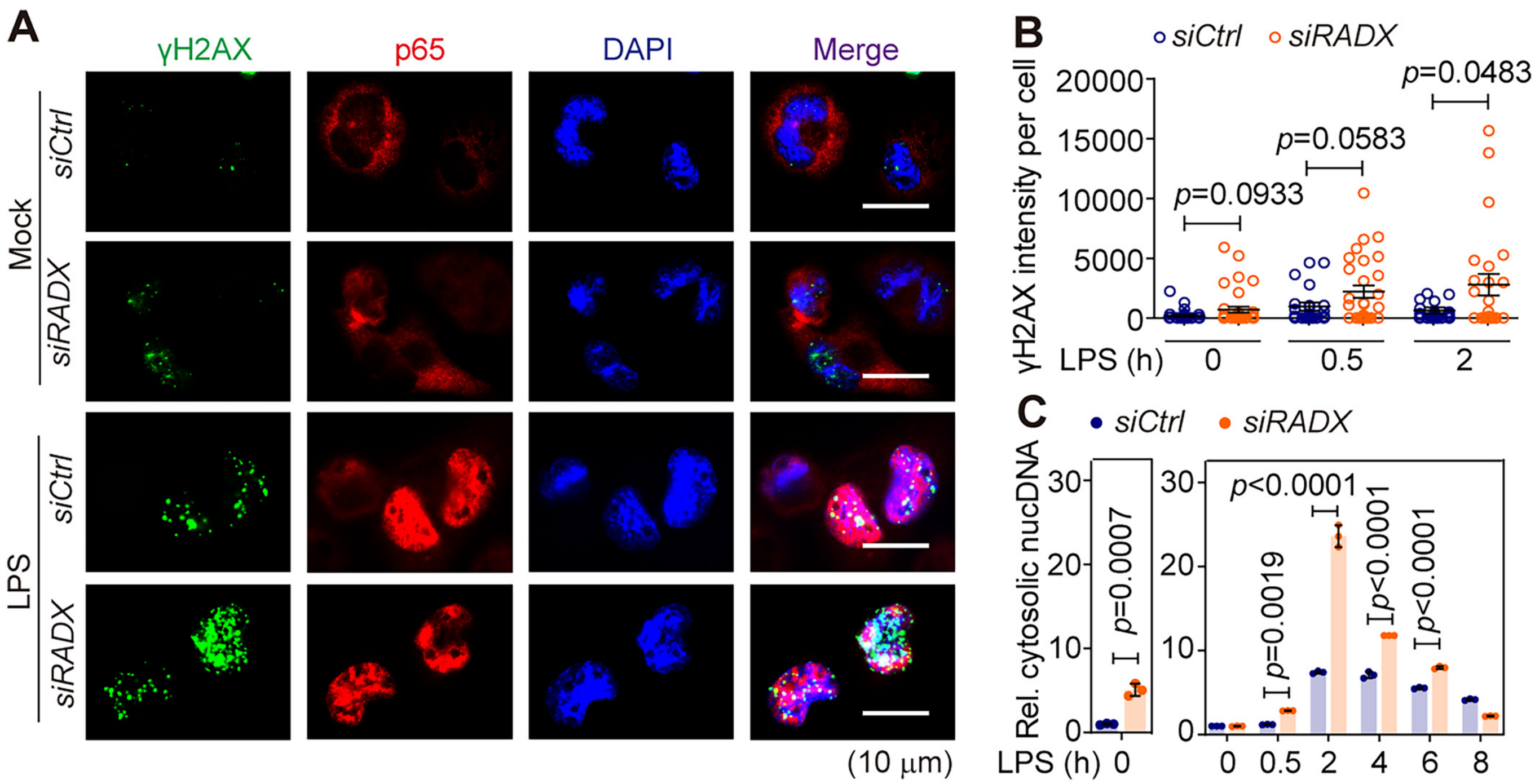

Figure EV3. RADX deficiency induces DNA damage in macrophages.

(**A**, **B**) Representative images (**A**) and quantification result (**B**) of confocal fluorescence analysis of THP-1-derived macrophages transfected with *siCtrl* or *RADX*-specific siRNA and stimulated with LPS for 2 h. $n = 31$ for *siCtrl*-Mock group, $n = 36$ for *siCtrl*-LPS 0.5 h group, $n = 30$ for *siCtrl*-LPS 2 h group, $n = 29$ for *siRADX*-Mock group, $n = 36$ for *siRADX*-LPS 0.5 h group, and $n = 24$ for *siRADX*-LPS 2 h group. Each data point represents a measurement from independent cell. (**C**) THP-1-derived macrophages transfected with *siCtrl* or *RADX*-specific siRNA were stimulated with LPS for indicated time. Cytoplasmic DNA were extracted for quantitative PCR analysis using nuclear DNA primers (Tert). Data are from $n = 3$ independent biological replicates. Data were presented as mean ± SEM and analyzed using one-way ANOVA (**C**) followed by Tukey–Kramer multiple-comparisons test, or two-tailed Mann–Whitney *U* test (**B**). The exact *P* values are indicated in the figures. Source data are available online for this figure.

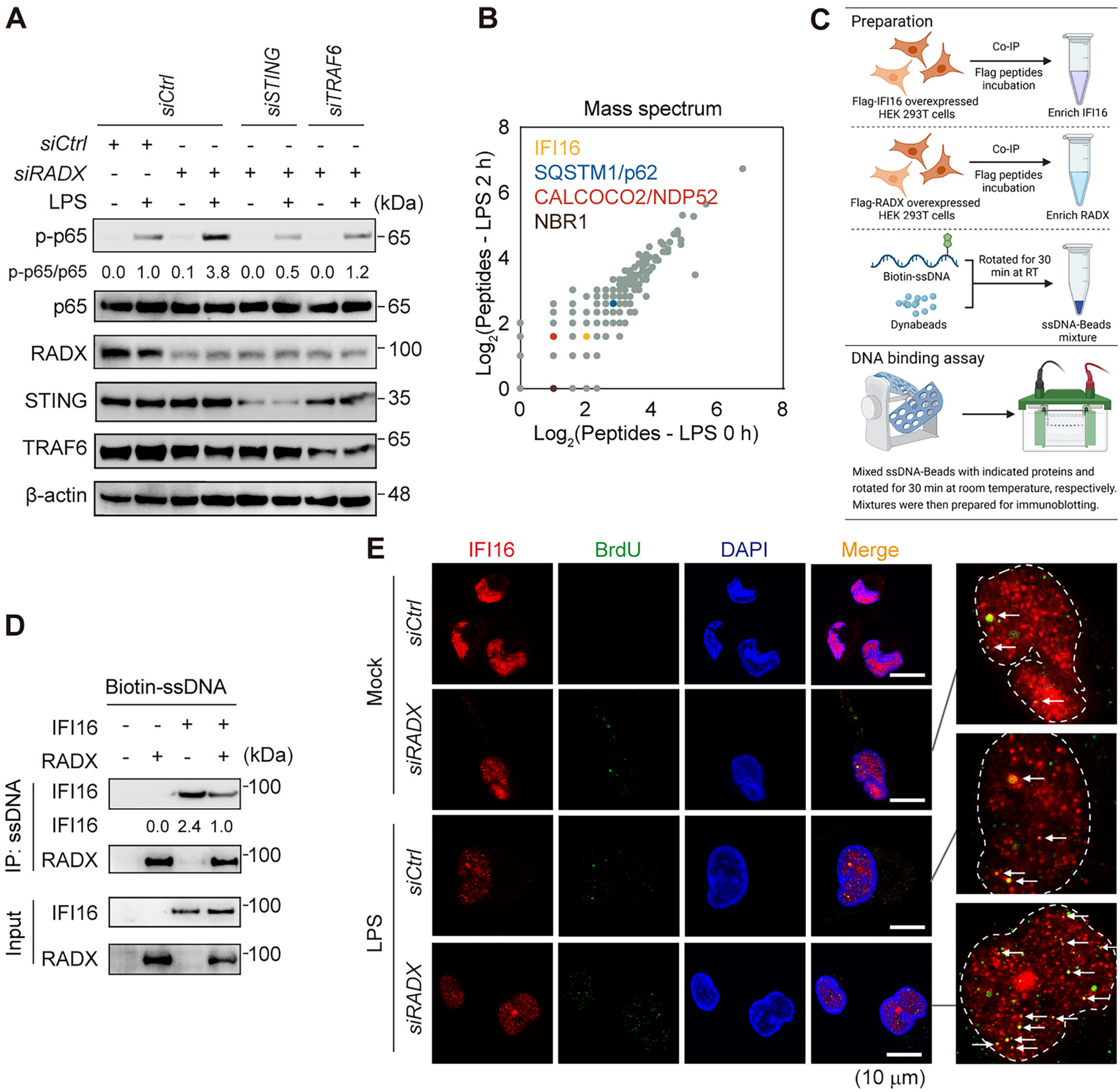

Figure EV4. RADX regulates NF-κB pathway activation.

(**A**) *siCtrl*- or *siRADX*-transfected THP-1-derived macrophages were co-transfected with *Ctrl*, *STING*, or *TRAF6*-specific siRNA and treated with LPS for 0.5 h. (**B**) Endogenous RADX in THP-1-derived macrophages was enriched by co-immunoprecipitation for mass spectrum analysis. (**C**) Workflow for in vitro ssDNA binding assay. (**D**) In vitro ssDNA binding assay of IFI16 in the presence (10 µg enriched protein for each) or absence of RADX. (**E**) Representative confocal imaging of macrophages transfected with *siCtrl* or *siRADX* and pre-labelled with BrdU for 48 h. LPS was applied for stimulation for 2 h. The dashed lines represented the position of nuclear membranes. Immunoblotting assays were repeated at least three times with independent biological replicates. Source data are available online for this figure.

© The Author(s)

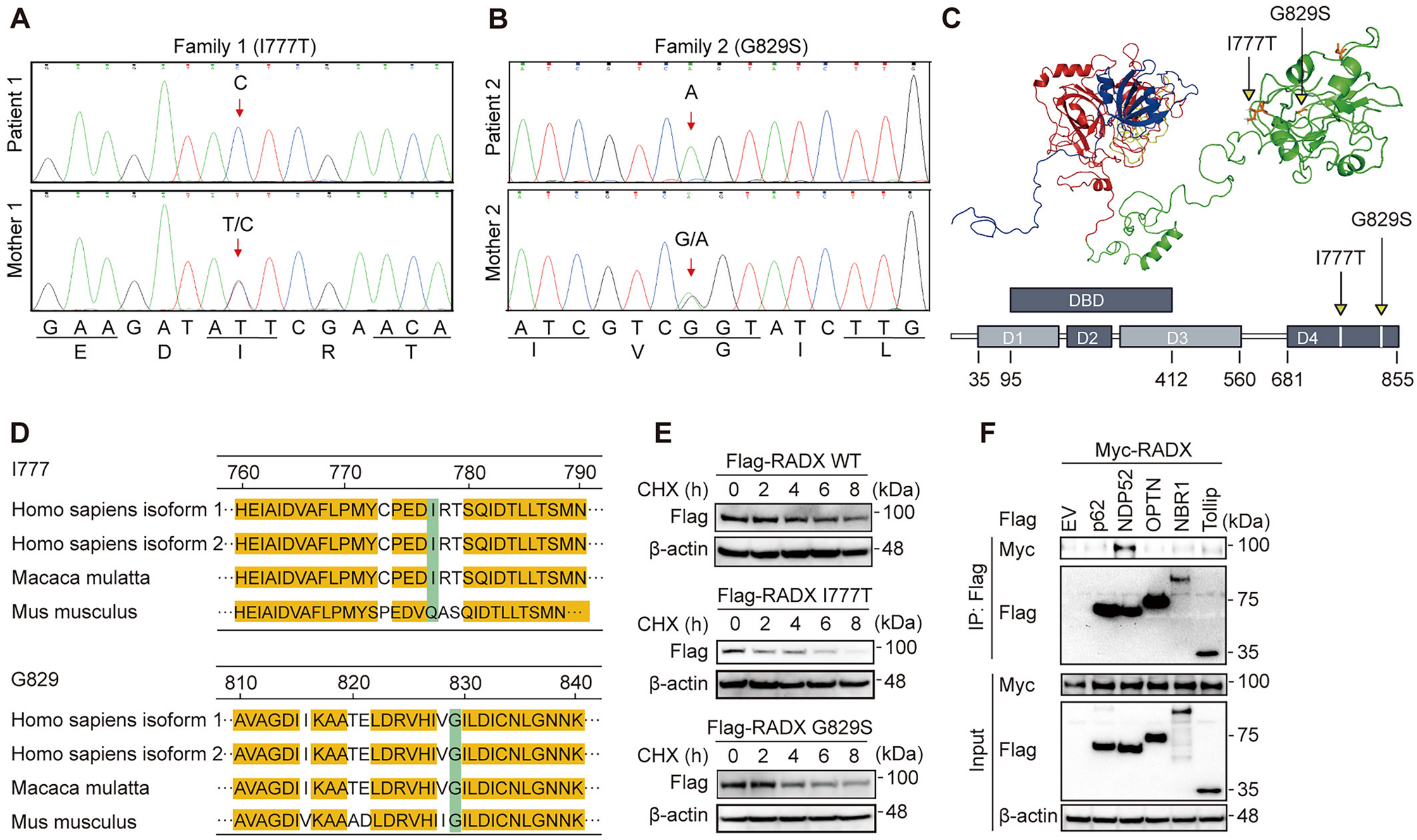

Figure EV5. Characteristics of RADX I777T and G829S variants.

(**A**, **B**) Sanger sequencing of patient 1 (I777T), patient 2 (G829S) and their mothers. M mutant allele, W wild-type allele. (**C**) Prediction of RADX protein structure by PyMoL. (**D**) Homology analysis of RADX I777T and G829S. (**E**) Immunoblotting of RADX abundance from cycloheximide (CHX)-treated HEK 293T cells transfected with Flag-RADX WT, I777T or G829S mutants. CHX working concentration: 100 µg/mL. $n = 3$ biological replicates. (**F**) HEK 293T cells were transfected with Myc-RADX, together with Flag-EV, p62, NDP52, OPTN, NBR1 or Tollip. Cell lysates were subjected to co-immunoprecipitation and immunoblotting. Immunoblotting assays were repeated at least three times with independent biological replicates. Source data are available online for this figure.

© The Author(s)

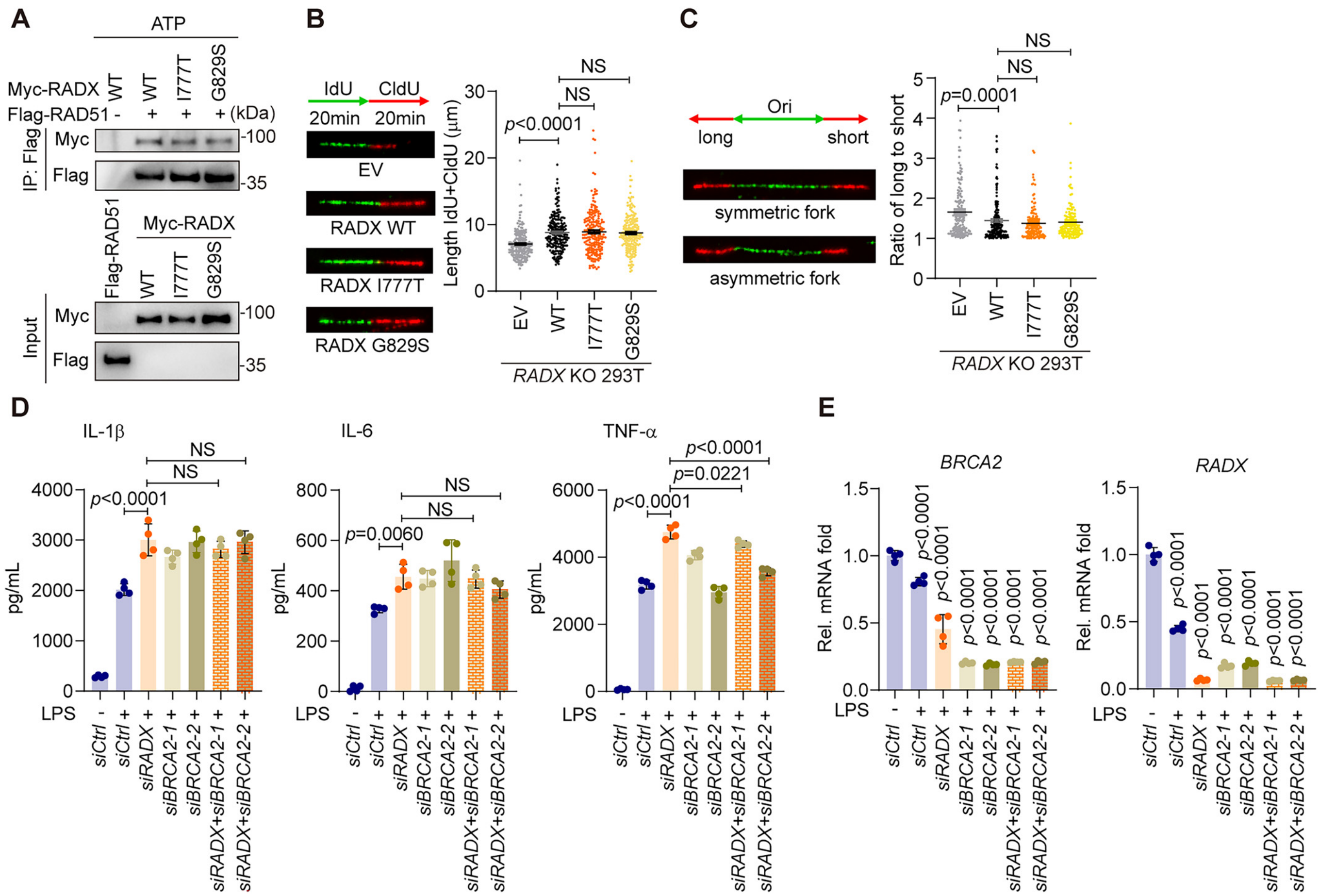

Figure EV6. RADX mutations show no effects on RAD51-ATP binding or replication fork progression.

(**A**) Purified Myc-RADX WT, I777T, G829S and ATP pre-bound Flag-RAD51 were incubated as indicated for 1 h. Mixtures were subjected to co-immunoprecipitation. (**B**, **C**) *RADX* KO HEK 293T cells transfected with EV, RADX WT, I777T or G829S were labeled with CldU and IdU as indicated and analyzed by DNA combing. Each data point represents a measurement from independent individual. $n = 201$ for each in (**B**); $n = 159$ for EV group, $n = 172$ for WT group, $n = 134$ for I777T group, $n = 129$ for G829S group in (**C**). (**D**) THP-1-derived macrophages transfected with *Ctrl*-, *RADX*- or *BRCA2*- specific siRNA were treated with LPS for 6 h. Supernatant were collected for ELISA. Data are from $n = 4$ independent biological replicates. (**E**) Knockdown efficiency of THP-1-derived macrophages transfected with *Ctrl*-, *RADX*- or *BRCA2*- specific siRNA with real-time PCR. Data are from $n = 4$ independent biological replicates. Data were presented as mean ± SEM and analyzed using one-way ANOVA followed by Tukey–Kramer multiple-comparisons test (**D**, **E**), or two-tailed Mann–Whitney *U* test (**B**, **C**). The exact *P* values are indicated in the figures. Source data are available online for this figure.

© The Author(s)

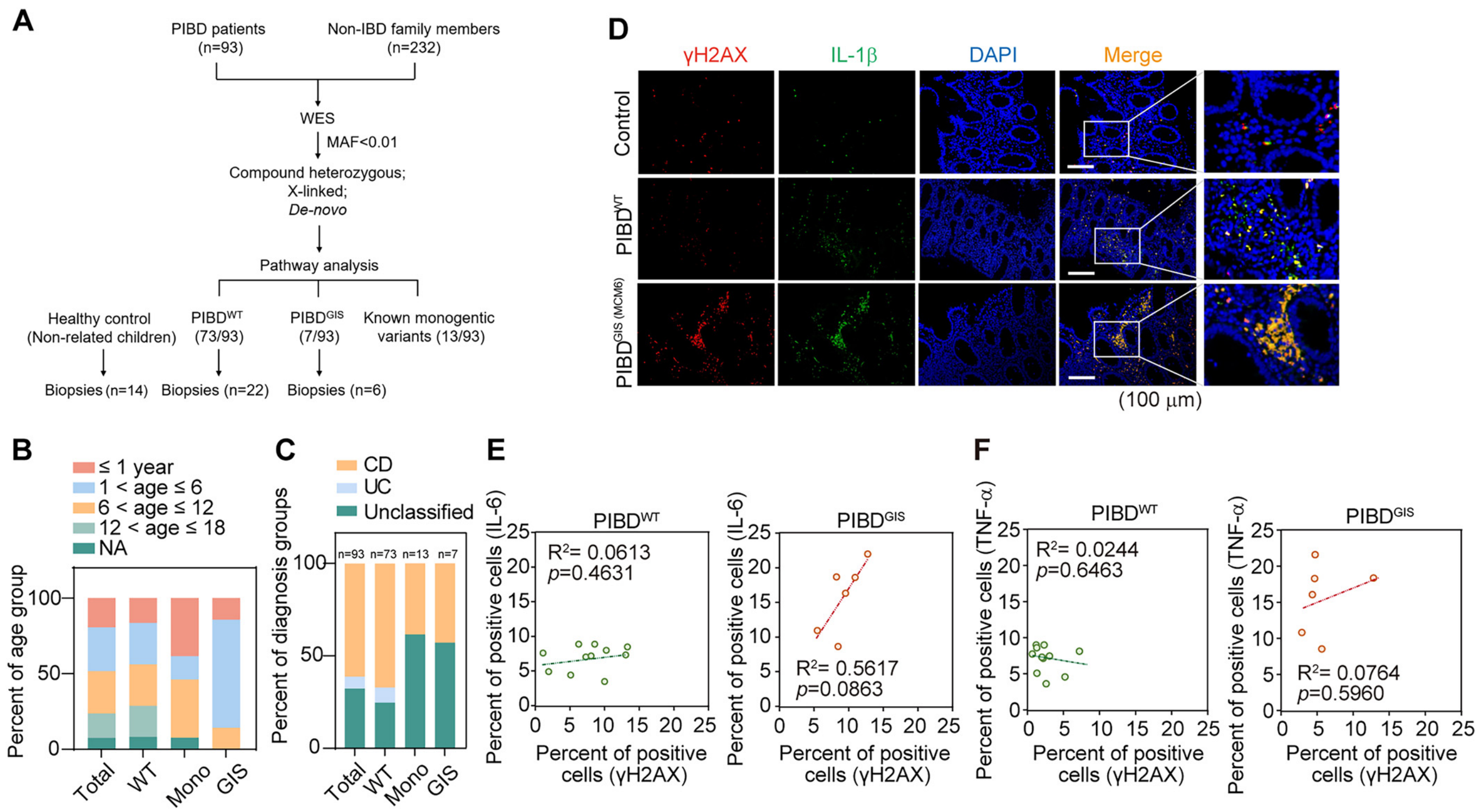

Figure EV7. Characteristics of PIBD cohort, genetic instability and inflammation.

(**A**) Schematic for patient enrollment. Non-IBD family members and affected PIBD patients were enrolled for whole-exome sequencing (WES) analysis. Compound heterozygous, X-linked recessive, and de novo mutations with minor allele frequency less than 0.01 from the patients ($n = 93$) was selected as candidate risk or disease causal genes for pathway analysis. Patients were stratified according to having known monogenic variants (13/93), variants that cause genomic instability ($PIBD^{GIS}$, 7/93), and other variants ($PIBD^{WT}$, 70/93). Colonic biopsies from non-related healthy control children ($n = 14$), $PIBD^{GIS}$ ($n = 6$) and $PIBD^{WT}$ ($n = 22$) were selected for genomic stability and inflammation study. Biopsy selection was based on material availability. (**B**, **C**) Age range and distribution (**B**) and IBD subtypes (**C**) of the PIBD cohort. (**D**) Representative immunofluorescence images of colon sections from a control subject, a $PIBD^{GIS}$ subject (biopsy from MCM6 mutated patient), and a $PIBD^{WT}$ subject. (**E**, **F**) Correlation analysis of γH2AX with indicated cytokines in $PIBD^{WT}$ and $PIBD^{GIS}$ subjects. The exact *P* values are indicated in the figures.

© The Author(s)
