# Supplementary material for: RADX protects against intestinal inflammation by restraining IFI16-mediated innate immunity
Source: EMBO Mol Med. 2026 Jul 24;18(9):3570–96. doi: 10.1038/s44321-026-00494-6 (PMC13562710; doi:10.1038/s44321-026-00494-6)
Supplement: Supplementary file 2 [file 44321_2026_494_MOESM2_ESM.pdf]

EMBO Molecular Medicine

RADX protects against intestinal inflammation by restraining IFI16-mediated innate immunity

Yuxia Zhang, Huifang Xian, Wanming Huang, Zhanghua Chen, Lili Li, Liya Xiong, Jianheng Zhu, Haolan Jin, Xiaotian Li, Lizhi Song, Peiyu Chen, Lu Ren, Wei Wang, Rongli Fang, Xixi Chen, Wen Lei, Dengfeng Zhang, Weiliang Liao, Yuxin Huang, Andrew Lew, Jun Cui, Sitang Gong, Jun Huang, Jaime S Rosa Duque, Min Zhi, Lanlan Geng, and Wenhao Zhou

Corresponding authors: Yuxia Zhang (yuxia.zhang@zhanglaboratory.com) , Wenhao Zhou (zhouwenhao@fudan.edu.cn), Lanlan Geng (2012690086@gzhmu.edu.cn), Min Zhi (zhimin@sysu.edu.cn)

Review Timeline:

Submission Date:	18th Mar 26
Editorial Decision:	15th Apr 26
Revision Received:	12th Jun 26
Editorial Decision:	7th Jul 26
Revision Received:	9th Jul 26
Accepted:	10th Jul 26

Editor: Zeljko Durdevic

Transaction Report:

(Note: With the exception of the correction of typographical or spelling errors that could be a source of ambiguity, letters and reports are not edited. Depending on transfer agreements, referee reports obtained elsewhere may or may not be included in this compilation. Referee reports are anonymous unless the Referee chooses to sign their reports.)

15th Apr 2026

Dear Prof. Zhang,

Thank you for the submission of your manuscript to EMBO Molecular Medicine. We have now received feedback from the two reviewers who agreed to evaluate your manuscript. Both referees recognize interest of the study but also raise serious concerns that should be addressed in a major revision. If you would like to discuss further the points raised by the referees, I am available to do so via email or video. Let me know if you are interested in this option.

Further consideration of a revision that addresses reviewers' concerns in full will entail a second round of review. EMBO Molecular Medicine encourages a single round of revision only and therefore, acceptance or rejection of the manuscript will depend on the completeness of your responses included in the next, final version of the manuscript. For this reason, and to save you from any frustrations in the end, I would strongly advise against returning an incomplete revision.

We would welcome the submission of a revised version within three months for further consideration. Please let us know if you require longer to complete the revision.

I look forward to receiving your revised manuscript.

Yours sincerely,

Zeljko Durdevic

Zeljko Durdevic
Senior Editor
EMBO Molecular Medicine

*****
When preparing your revised manuscript, please refer to our guidelines: https://link.springer.com/journal/44321/submission-guidelines#cms-Revised-submissions. We perform an initial quality control of all revised manuscripts before re-review; failure to include requested items will delay the evaluation of your revision.

We require:

1) A .docx formatted version of the manuscript text (including legends for main figures, EV figures and tables). Please make sure that the changes are highlighted to be clearly visible.

2) Individual production quality figure files as .eps, .tif, .jpg (one file per figure). For guidance, download the 'Figure Guide PDF': https://media.springernature.com/original/springer-cms/rest/v1/content/27825798/data/v1.

3) A .docx formatted letter INCLUDING the reviewers' reports and your detailed point-by-point responses to their comments. As part of the EMBO Press transparent editorial process, the point-by-point response is part of the Review Process File (RPF), which will be published alongside your paper.

4) A complete author checklist, which you can download from our author guidelines. Please insert information in the checklist that is also reflected in the manuscript. The completed author checklist will also be part of the RPF.

5) Please note that all corresponding authors are required to supply an ORCID ID for their name upon submission of a revised manuscript.

6) It is mandatory to include a 'Data Availability' section after the Materials and Methods. Before submitting your revision, primary datasets produced in this study need to be deposited in an appropriate public database, and the accession numbers and database listed under 'Data Availability'. Please remember to provide a reviewer password if the datasets are not yet public.

In case you have no data that requires deposition in a public database, please state so in this section. Note that the Data Availability Section is restricted to new primary data that are part of this study.

7) For data quantification: please specify the name of the statistical test used to generate error bars and P values, the number (n) of independent experiments (specify technical or biological replicates) underlying each data point and the test used to calculate p-values in each figure legend. The figure legends should contain a basic description of n, P and the test applied. Graphs must include a description of the bars and the error bars (s.d., s.e.m.).

8) At EMBO Press we ask authors to provide source data for the main manuscript figures. You will receive a separate email with instructions for providing source data with your revised manuscript, including how to upload and organize the files.

9) Our journal encourages inclusion of *data citations in the reference list* to directly cite datasets that were re-used and obtained from public databases. Data citations in the article text are distinct from normal bibliographical citations and should directly link to the database records from which the data can be accessed. In the main text, data citations are formatted as follows: "Data ref: Smith et al, 2001" or "Data ref: NCBI Sequence Read Archive PRJNA342805, 2017". In the Reference list, data citations must be labeled with "[DATASET]". A data reference must provide the database name, accession number/identifiers and a resolvable link to the landing page from which the data can be accessed at the end of the reference.

10) We replaced Supplementary Information with Expanded View (EV) Figures and Tables that are collapsible/expandable online. A maximum of 5 EV Figures can be typeset. EV Figures should be cited as 'Figure EV1, Figure EV2" etc... in the text and their respective legends should be included in the main text after the legends of regular figures.

- For the figures that you do NOT wish to display as Expanded View figures, they should be bundled together with their legends in a single PDF file called *Appendix*, which should start with a short Table of Content. Appendix figures should be referred to in the main text as: "Appendix Figure S1, Appendix Figure S2" etc.

- Additional Tables/Datasets should be labeled and referred to as Table EV1, Dataset EV1, etc. Legends have to be provided in a separate tab in case of .xls files. Alternatively, the legend can be supplied as a separate text file (README) and zipped together with the Table/Dataset file.

11) The paper explained: EMBO Molecular Medicine articles are accompanied by a summary of the articles to emphasize the major findings in the paper and their medical implications for the non-specialist reader. Please provide a draft summary of your article highlighting

- the medical issue you are addressing,

- the results obtained and

- their clinical impact.

This may be edited to ensure that readers understand the significance and context of the research. Please refer to any of our published articles for an example.

12) Author contributions: You will be asked to provide CRediT (Contributor Role Taxonomy) terms in the submission system. These replace a narrative author contribution section in the manuscript.

13) A Conflict of Interest statement should be provided in the main text.

14) Every published paper includes a 'Synopsis' to further enhance discoverability. Synopses are displayed on the journal webpage and are freely accessible to all readers. They include a short stand first (maximum of 300 characters, including space) as well as 2-5 one-sentences bullet points that summarizes the paper. Please write the bullet points to summarize the key NEW findings. They should be designed to be complementary to the abstract - i.e. not repeat the same text. We encourage inclusion of key acronyms and quantitative information (maximum of 30 words / bullet point). Please use the passive voice. Please attach these in a separate file or send them by email, we will incorporate them accordingly.

Please also suggest a striking image or visual abstract to illustrate your article as a PNG file 550 px wide x 300-600 px high.

15) Include a Reagents and Tools Table as part of the Methods section, which can be downloaded from our author guidelines.

EMBO Molecular Medicine has a "scooping protection" policy, whereby similar findings that are published by others during review or revision are not a criterion for rejection. Should you decide to submit a revised version, I do ask that you get in touch after three months if you have not completed it, to update us on the status.

***** Reviewer's comments *****

Referee #1 (Comments on Novelty/Model System for Author):

I rated the manuscript highly for technical quality because it uses a well-structured and multi-layered experimental design that combines complementary in vivo, ex vivo, and mechanistic approaches. The authors employ two acute murine colitis models, DSS and TNBS, together with bone marrow transplantation, macrophage-based functional assays, biochemical binding experiments, confocal analyses, patient-derived PBMCs and intestinal biopsy material, and a broader PIBD cohort analysis. This provides a coherent body of evidence supporting the central conclusion that RADX restrains intestinal inflammation by limiting DNA damage/ssDNA-driven IFI16 signaling. In particular, the data linking RADX deficiency to increased DNA damage, enhanced IFI16-ssDNA association, stronger NF-κB activation, increased IFI16-ASC interaction, and elevated IL-1β production are persuasive and mechanistically integrated. I also considered the novelty to be high, because the study connects a replication fork protection factor, RADX, to intestinal inflammatory disease and proposes a conceptually interesting RADX-IFI16 axis that links genome instability to innate immune activation. This is not simply an incremental extension of known IBD biology, but rather a mechanistic bridge between DNA damage control and inflammatory signaling, which adds substantial conceptual value. I rated the medical impact as medium rather than high. The work is clearly relevant to IBD and PIBD pathogenesis, and the inclusion of patient-associated RADX variants together with the RI-1 intervention experiments gives the study translational interest. However, the direct clinical implications remain somewhat preliminary, because the human genetic evidence is based on only two RADX-variant cases, the proposed "genomic instability" subgroup remains more suggestive than fully validated, and the pharmacologic data, while intriguing, do not yet establish a highly specific therapeutic avenue. I considered the model system to be adequate and appropriate for the questions asked. The DSS and TNBS models are suitable for testing whether Radx deficiency increases susceptibility to intestinal inflammation, and the bone marrow transplantation experiments are a particular strength because they support a dominant role for hematopoietic cells. The macrophage experiments are also well aligned with the proposed IFI16/NF-κB/inflammasome mechanism. That said, the model system is not comprehensive: both colitis models are acute and do not fully capture chronic relapsing IBD, and epithelial contributions are not deeply explored. The study could be further strengthened by validation in chronic colitis settings, cell type-specific deletion models, or human intestinal organoid/co-culture systems. I do not see any major ethical concerns beyond the usual requirements for appropriate animal welfare measures in colitis models and clear ethical approval, informed consent, and privacy protection for the human samples.

Referee #1 (Remarks for Author):

Recommendation: Major revision
This manuscript investigates the role of RADX in intestinal inflammation and proposes that RADX restrains IFI16-dependent inflammatory signaling in colitis. The topic is interesting, and the study integrates mouse models, macrophage-based experiments, and patient-associated observations. The overall direction of the work is clear. At the same time, several conclusions would benefit from more precise framing, and some aspects of interpretation and reporting would be helpful to refine before the manuscript can be considered further.

Major points
1. The data support that RADX deficiency is associated with increased DNA damage, enhanced ssDNA/IFI16-related signaling, and stronger NF-κB/inflammasome activation. However, in the Abstract, Introduction, and Discussion, the current wording sometimes gives the impression that the RADX-IFI16 axis is sufficient to account for the in vivo colitis phenotype. Based on the current data, it may be more accurate to present this as the principal mechanism supported in this study, while leaving room for the possibility that additional pathways may also contribute.

2. The manuscript concludes that RADX deficiency exacerbates colitis in a hematopoietic cell-dependent manner and further suggests that hematopoietic cells, rather than epithelial cells, are the primary driver of the phenotype. The bone marrow transplantation and macrophage-related data do support an important contribution from the hematopoietic compartment. At the same time, the epithelial data are relatively limited. It may therefore be helpful to state that the hematopoietic compartment appears to account for much of the phenotype in the current models, while noting that additional cellular contributions are not yet fully excluded.

3.The identification of two RADX variants in two CD patients is interesting and potentially relevant, especially given the reduced RADX protein and increased γH2AX/inflammatory readouts in patient material. However, with only two cases, it would be helpful for the manuscript to frame these findings as supportive and hypothesis-generating, rather than definitive. A more explicit acknowledgement of sample-size limitations in the Discussion would strengthen this section.

4. The RI-1 data are useful in showing that this pathway is functionally relevant. However, the manuscript also shows that RI-1 reduces inflammatory signaling in control cells and improves colitis in WT mice. In that context, it may be clearer to describe RI-1 as alleviating inflammatory and DNA-damage-related phenotypes in this system, rather than specifically restoring RADX-dependent function. A corresponding adjustment in the Abstract, Results, and Discussion would improve consistency.

Minor points
1. Since the study mainly uses DSS- and TNBS-induced acute colitis models, it would be helpful to note more directly that the relevance to chronic IBD remains to be established.
2. At present, this term reads more as a descriptive category proposed within this cohort analysis. A brief clarification of how the term is being used in this manuscript would make the interpretation clearer.
3. Please ensure that sample size, number of biological replicates, statistical tests, and the meaning of error bars are reported consistently for the key in vivo, patient-derived, and mechanistic experiments.
4. There are several grammatical issues and awkward constructions in the Results and Discussion sections, and some sentences could be revised for clarity and precision.

Overall, I recommend major revision. The study is of interest, and the main issues relate primarily to framing, interpretation, and consistency of reporting.

Referee #2 (Remarks for Author):

This study identifies RADX as an important regulator of intestinal homeostasis, protecting against inflammation by limiting the detection of single stranded DNA (ssDNA) by the innate immune sensor IFI16. The authors show that loss of RADX, particularly in hematopoietic cells, results in ssDNA accumulation, leading to excessive activation of the NF κB pathway and inflammasome signaling. Using mouse models of colitis, biochemical approaches, and data from a pediatric inflammatory bowel disease (PIBD) cohort, they further demonstrate that rare RADX variants reduce protein stability and enhance pro inflammatory responses. Notably, pharmacological inhibition of RAD51 with RI 1 reduces DNA damage and improves disease severity in colitis models, pointing to a potential therapeutic strategy for a subset of IBD patients with underlying genomic instability.
While the findings are of interest, several important issues require clarification.
Major points
1. Many experimental conditions are not described with sufficient clarity, which limits the reader's ability to fully understand the results and assess the authors' interpretations.
2. The number of biological replicates is not indicated for most experiments in the figure legends, making it difficult to assess the robustness and reproducibility of the data.
3. Do Radx-/- mice express a truncated RADX protein? are there any basal physiological alterations in these mice, such as changes in body weight, growth rate, or general development?
4. In Figure 1H, the sample number does not appear to correspond to that shown in Figure 1F. This discrepancy could affect the statistical analysis. Could the authors clarify this point?
5. The authors should clearly describe the mouse treatment protocol, including the timing and duration of DSS administration and the procedures used to collect the final experimental readouts.
6. It is unclear why the authors did not directly conclude that macrophages play a major role in RADX mediated colitis, rather than broadly attributing the effect to hematopoietic cells. This distinction is particularly important given that dendritic cells do not appear to exhibit markers of genomic instability or secrete IL 1β in this study.
7. The bone marrow transplantation procedure described in Figure 2 is not clearly explained, either in the main text or in the Materials and Methods section. Specifically, it is unclear whether the recipient mice underwent bone marrow ablation prior to transplantation. If not, the authors should explain how the donor (KO) bone marrow was able to effectively reconstitute or outcompete the endogenous bone marrow of the WT recipients.
8. In Fig. S3C, the authors use the Tert gene to assess the presence of nuclear DNA in the cytosol. It is unclear why Tert was chosen as a representative marker. How can the authors be certain that Tert specifically, or preferentially, would be released into the cytosol following DNA damage? Additional justification or validation of this approach would be helpful.
9. In Figure 4A, the number of biological replicates is not indicated. Additionally, if IFI16 is proposed as a key regulator of this pathway, one would expect a more pronounced reduction in p p65 levels in siIFI16 treated cells, similar to the effect observed with siSTING in Figure S4A. The authors should clarify this discrepancy and discuss the relative contribution of IFI16 to NF κB activation.
10. In the in vitro DNA binding assay (Figure 4D), the working concentrations of RADX and IFI16 are not indicated. In addition, no concentration dependent DNA binding analysis is presented. Including these details and experiments would be important to assess the specificity and strength of the reported interactions.
11. In Figure 4E, it is unclear whether the ssDNA foci are localized in the nucleus or in the cytoplasm. The authors should clarify the subcellular localization of these ssDNA signals.
12. The quantification of colocalization between ASC and IFI16 is missing.
13. Authors should provide evidence showing STING localization in the nucleus.

Minor points-
1. The labels for some histological and immunofluorescence markers, particularly those shown in blue, green, and red on a black background, are difficult to read, which affects figure clarity.

2. In Figure 3H (IL 1β secretion), is the difference between control and LPS stimulated WT BMDMs not statistically significant?
3. In Figure 5F, are the differences observed for IL 1β following 12 hour LPS treatment and for TNF α following 24 hour LPS treatment not statistically significant?

Point-by-point Response to Referees' Comments

<u>Referee #1 (Comments on Novelty/Model System for Author):</u>

I rated the manuscript highly for technical quality because it uses a well-structured and multi-layered experimental design that combines complementary in vivo, ex vivo, and mechanistic approaches. The authors employ two acute murine colitis models, DSS and TNBS, together with bone marrow transplantation, macrophage-based functional assays, biochemical binding experiments, confocal analyses, patient-derived PBMCs and intestinal biopsy material, and a broader PIBD cohort analysis. This provides a coherent body of evidence supporting the central conclusion that RADX restrains intestinal inflammation by limiting DNA damage/ssDNA-driven IFI16 signaling. In particular, the data linking RADX deficiency to increased DNA damage, enhanced IFI16-ssDNA association, stronger NF-κB activation, increased IFI16-ASC interaction, and elevated IL-1β production are persuasive and mechanistically integrated. I also considered the novelty to be high, because the study connects a replication fork protection factor, RADX, to intestinal inflammatory disease and proposes a conceptually interesting RADX-IFI16 axis that links genome instability to innate immune activation. This is not simply an incremental extension of known IBD biology, but rather a mechanistic bridge between DNA damage control and inflammatory signaling, which adds substantial conceptual value. I rated the medical impact as medium rather than high. The work is clearly relevant to IBD and PIBD pathogenesis, and the inclusion of patient-associated RADX variants together with the RI-1 intervention experiments gives the study translational interest. However, the direct clinical implications remain somewhat preliminary, because the human genetic evidence is based on only two RADX-variant cases, the proposed "genomic instability" subgroup remains more suggestive than fully validated, and the pharmacologic data, while intriguing, do not yet establish a highly specific therapeutic avenue. I considered the model system to be adequate and appropriate for the questions asked. The DSS and TNBS models are suitable for testing whether Radx deficiency increases susceptibility to intestinal inflammation, and the bone marrow transplantation experiments are

a particular strength because they support a dominant role for hematopoietic cells. The macrophage experiments are also well aligned with the proposed IFI16/NF-κB/inflammasome mechanism. That said, the model system is not comprehensive: both colitis models are acute and do not fully capture chronic relapsing IBD, and epithelial contributions are not deeply explored. The study could be further strengthened by validation in chronic colitis settings, cell type-specific deletion models, or human intestinal organoid/co-culture systems. I do not see any major ethical concerns beyond the usual requirements for appropriate animal welfare measures in colitis models and clear ethical approval, informed consent, and privacy protection for the human samples.

Recommendation: Major revision

This manuscript investigates the role of RADX in intestinal inflammation and proposes that RADX restrains IFI16-dependent inflammatory signaling in colitis. The topic is interesting, and the study integrates mouse models, macrophage-based experiments, and patient-associated observations. The overall direction of the work is clear. At the same time, several conclusions would benefit from more precise framing, and some aspects of interpretation and reporting would be helpful to refine before the manuscript can be considered further.

Major points

Comment 1. The data support that RADX deficiency is associated with increased DNA damage, enhanced ssDNA/IFI16-related signaling, and stronger NF-κB/inflammasome activation. However, in the Abstract, Introduction, and Discussion, the current wording sometimes gives the impression that the RADX-IFI16 axis is sufficient to account for the in vivo colitis phenotype. Based on the current data, it may be more accurate to present this as the principal mechanism supported in this study, while leaving room for the possibility that additional pathways may also contribute.

Response: Thank you for your thoughtful suggestion. We fully agree that multiple factors including genetics, immunology, epithelial barrier function, and environment triggers contribute to the pathophysiology of IBD. Genome-wide associated studies have identified

more than 200 genetic loci associated with IBD(Liu *et al*, 2015). These genes encode proteins involved in innate and adaptive immunity, as well as mucosal barrier integrity. In genetically predisposed individuals, dysregulated intestinal immunity can lead to chronic relapsing immune activation and pathological manifestations of IBD(Na *et al*, 2019).

In this study, we observed increased DNA damage, enhanced ssDNA/IFI16-mediated signaling, and stronger NF-κB/inflammasome activation in RADX deficient macrophages. These findings support a role for this axis as a principal mechanism contributing to the aggravated colitis phenotype in *Radx*$^{-/-}$ mice. However, we acknowledge that other pathways may also be involved, and our study does not exclude their potential contributions. We have revised the manuscript to present the RADX-IFI16 axis as a key mechanism rather than the sole cause, in line with your recommendation.

Current wording in Abstract

"*Loss of RADX promotes ssDNA accumulation, which unleashes IFI16-dependent activation of NF-κB pathway and inflammasome assembly, thereby promoting colitis development.*" and "*Notably, the RAD51 inhibitor RI-1 alleviated the colitis phenotypes in both wild type and Radx-deficiency mice*"

Current wording in Introduction

"*In this study, we found that RADX protects against intestinal inflammation by maintaining genomic stability, limiting ssDNA accumulation, and competing with IFI16 for DNA binding, thereby attenuating NF-κB and inflammasome activation in macrophages.*" and "*Although the pathogenesis of IBD involves multiple genetic, immunological, and environmental factors, this study identifies the RADX-IFI16 axis as a contributor to intestinal inflammation*".

Current wording in Discussion

"*In summary, our study demonstrates that Radx deficiency leads to increased DNA damage, amplifies IFI16-dependent NF-κB signaling and inflammasome activation, thereby contributing to colitis development.*" and "*Although additional pathways may also modulate*

disease, our findings identify the RADX-IFI16 axis as an important contributor in macrophages".

Comment 2. The manuscript concludes that RADX deficiency exacerbates colitis in a hematopoietic cell-dependent manner and further suggests that hematopoietic cells, rather than epithelial cells, are the primary driver of the phenotype. The bone marrow transplantation and macrophage-related data do support an important contribution from the hematopoietic compartment. At the same time, the epithelial data are relatively limited. It may therefore be helpful to state that the hematopoietic compartment appears to account for much of the phenotype in the current models, while noting that additional cellular contributions are not yet fully excluded.

Response: Thank you for the constructive comment. We agree that our data support a major contribution from the hematopoietic compartment, particularly macrophages, to the exacerbated colitis phenotype in *Radx*$^{-/-}$ mice, as evidenced by bone marrow transplantation, RADX expression profiling in different cell types, and macrophage functional assays.

However, we also observed the basal decrease in MUC2 expression in *Radx*$^{-/-}$ mice (**Fig. 6H**), prompting us to examine whether RADX deficiency might regulate MUC2 expression or goblet cell death. We found that RADX deficiency decreases the transcriptional level of MUC2 at baseline, but not upon TNF stimulation (**Fig. EV2C**). Moreover, RADX does not regulates goblet cells cell death (**Fig. EV2D-E**). We acknowledge that our current epithelial data are limited. Definitive assessment of epithelial-specific contributions will require further studies using conditional knock mice (e.g., Villin-Cre) or intestinal organoids.

In light of your suggestion, we have revised the manuscript to state that hematopoietic compartment, especially macrophages, appears to account for dominant phenotype in our models, while noting that additional cellular contributions (e.g., from epithelial cells) are not fully excluded. We appreciate your guidance in helping us present our conclusions more precisely.

Current wording in Result

"*Taken together, these results suggest that Radx deficiency in macrophages accounts for much of the exacerbated colitis phenotype. However, contributions from additional cell types, including epithelial cells, are not yet fully excluded and require further investigation*".

Comment 3. The identification of two RADX variants in two CD patients is interesting and potentially relevant, especially given the reduced RADX protein and increased γH2AX/inflammatory readouts in patient material. However, with only two cases, it would be helpful for the manuscript to frame these findings as supportive and hypothesis-generating, rather than definitive. A more explicit acknowledgement of sample-size limitations in the Discussion would strengthen this section.

Response: Thank you for the suggestion. In this study, we observed decreased RADX protein abundance, increased γH2AX signaling, and hyperactivation of inflammatory pathway in PBMCs and colonic biopsies from RADX variant patients, which are consistent with experimental models (**Fig. 5**). We agree that the identification of two RADX variants in two CD patients is potentially relevant, but the sample size is limited, these findings should be interpreted as supportive and hypothesis-generating rather than definitive.

In line with your suggestion, we have revised the Discussion section to explicitly acknowledge the small number of cases. We now state that these patient data provide preliminary evidence linking RADX deficiency to increased DNA damage and inflammatory readouts in human IBD, but larger cohort studies are needed to establish the frequency and significance of RADX variants in CD.

Current wording in Discussion

"*Two RADX variants (I777T and G829S, respectively) identified in CD patients provide clinical support for our findings. Patient-derived PBMCs and colonic biopsies exhibited reduced RADX protein, elevated DNA damage, as well as hyperinflammatory signaling. However, a larger sample size is needed to strengthen the clinical relevance".*

Comment 4. The RI-1 data are useful in showing that this pathway is functionally relevant. However, the manuscript also shows that RI-1 reduces inflammatory signaling in control cells

and improves colitis in WT mice. In that context, it may be clearer to describe RI-1 as alleviating inflammatory and DNA-damage-related phenotypes in this system, rather than specifically restoring RADX-dependent function. A corresponding adjustment in the Abstract, Results, and Discussion would improve consistency.

Response: Thank you for reminding us of this point. We have revised the manuscript to describe RI-1 as alleviating inflammatory and DNA-damage-related phenotypes. Corresponding adjustments have been made in the Abstract, Results, and Discussion to ensure consistency.

Current wording in Abstract

"*Notably, the RAD51 inhibitor RI-1 alleviated the colitis phenotypes in both wild-type and Radx-deficiency mice*".

Current wording in Introduction

"*Pharmacological inhibition of RAD51 with RI-1 alleviated DNA damage, inflammation, and colitis phenotypes*".

Current wording in Results

"*We hypothesized that pharmacologically inhibiting RAD51 could ameliorates DNA damage and inflammatory signaling*".

Current wording in Discussion

"*In summary, our study demonstrates that Radx deficiency leads to increased DNA damage, amplifies IFI16-dependent NF-κB signaling and inflammasome activation, thereby contributing to colitis development. The efficacy of RAD51 inhibition in alleviating this pathology underscores the therapeutic potential of targeting this axis*".

Minor points

Comment 5. Since the study mainly uses DSS- and TNBS-induced acute colitis models, it would be helpful to note more directly that the relevance to chronic IBD remains to be

established.

Response: Thank you for the comment. We agree that the Dextran sulfate sodium (DSS) and trinitrobenzene sulfonic acid (TNBS) models used in this study are acute colitis models-mimicking UC-like and CD-like acute phenotype, respectively-and do not fully recapitulate the chronic, relapsing nature of human IBD(Hartwig *et al*, 2021).

To preliminarily explore potential chronic effects, we examined the chronic colitis phenotype in *Radx*$^{-/-}$ mice using repeated DSS administration in revision(Wirtz *et al*, 2017) (**Response Fig. 1)**. Although colon length did not differ between WT and *Radx*$^{-/-}$ mice after 3 cycles of DSS/water administration, we observed increased DAI, histological score, and IL1β and γH2AX signals in the colon of *Radx*$^{-/-}$ mice However, we acknowledge that even repeated chemical administration does not fully capture the chronic phenotype observed in humans(Hartwig *et al.*, 2021). Genetically manipulated mouse models (e.g., *Il10*$^{-/-}$ mice and *Muc1/2*$^{-/-}$ mice) better reflect long-lasting chronic inflammation in IBD(Valatas *et al*, 2013).

We have revised the Results to state more directly that the relevance of our findings to chronic IBD remains to be established. We now explicitly note that further studies using genetic models are needed to determine whether RADX-IFI16 axis contributes to long-lasting chronic inflammation in IBD.

Current wording in Results

"*We further examined the chronic colitis phenotype in Radx-/- mice using repeated DSS administration (Wirtz et al, 2017) (Fig. EV1C). Although colon length did not differ between WT and Radx-/- mice after 3 cycles of DSS/water administration, we observed increased DAI, histological scores, and IL-1β and γH2AX signals in distal colon of Radx-/- mice (Fig. EV1, D-I), which is consistent with acute colitis phenotype. However, whether RADX contributes to chronic, relapsing colitis is needed further confirmation using genetically manipulated mouse model (e.g., Il10*$^{-/-}$ *mice and Muc1/2*$^{-/-}$ *mice)*".

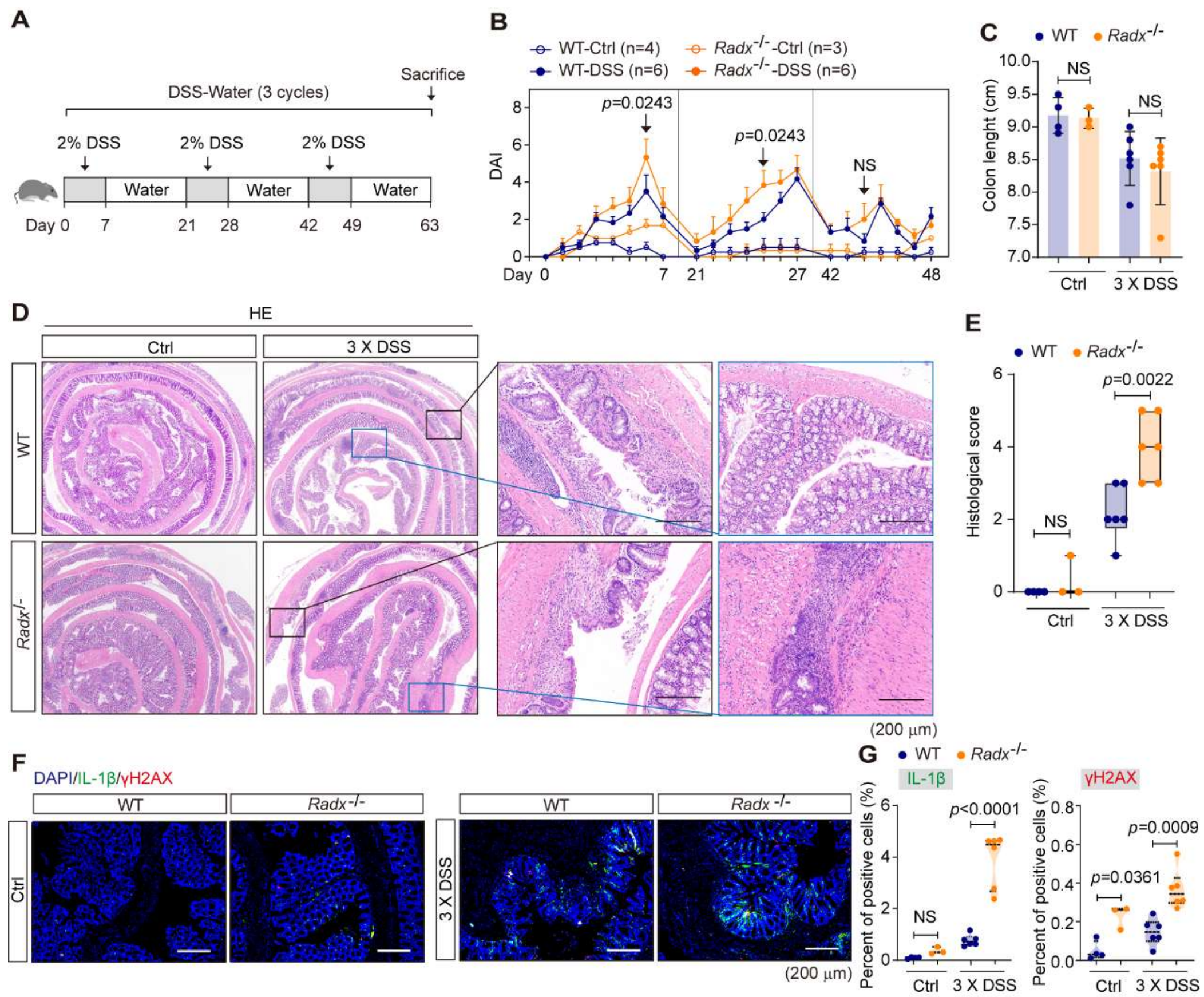

Response Fig. 1| *Radx* knockout aggravates DSS-induced chronic colitis. (**A**) Schematic of DSS-induced chronic colitis model. (**B-C**) Disease activity index (DAI) (B) and colon lengths (C) of WT or *Radx*$^{-/-}$ mice in DSS-induced acute colitis model. n=4 in WT-Ctrl group, n=6 in WT-DSS group, n=3 in *Radx*$^{-/-}$-Ctrl group, and n=6 in *Radx*$^{-/-}$-DSS group. Each data point represents a measurement from independent individual. (**D-G**) Representative images of hematoxylin-eosin (H&E) staining (D), histological scores (E), immunofluorescence analysis (F), and quantification of immunofluorescence staining (G) of distal colon segments from WT or *Radx*$^{-/-}$ mice in DSS-induced chronic colitis model. Each data point represents a measurement from independent individual. Data were presented as mean±SEM and analyzed using one-way (Figure EV1E, 1G, and 1I) or two-way (Figure EV1D) ANOVA followed by Tukey-Kramer multiple-comparisons test. Source data are available online for this figure.

Comment 6. At present, this term reads more as a descriptive category proposed within this cohort analysis. A brief clarification of how the term is being used in this manuscript would

make the interpretation clearer.

Response: Thank you for this important suggestion. We agree that the term "$PIBD^{GIS}$" required clearer definition within the manuscript. In our revised text, we now explicitly state that "$PIBD^{GIS}$" denotes the subgroup of PIBD patients who carry rare germline variants in genes predicted to be involved in genomic stability maintenance (Table EV2). These patients are hypothesized to exhibit elevated DNA damage signaling, representing a "genomic instability signature" (GIS) subgroup.

Specifically, we performed whole-exome sequencing (WES) on 93 PIBD patients and 232 family members. From a curated gene list related to genomic stability, we identified putative pathogenic variants in 7 genes across 7 patients (7.5% of the cohort), whom we designated as the $PIBD^{GIS}$ subgroup (Fig. 7A).

To distinguish these patients from those with known monogenetic cause of IBD (e.g., *XIAP*, *FOXP3*, *CYBB*, and *IL10RB*), we classified the latter as $PIBD^{Mono}$ subgroup. The $PIBD^{Mono}$ subgroup most commonly occurs in children < 6 years of age and accounts for 10%-20% of very-early-onset IBD (VEO-IBD) case(Konys *et al*, 2025; Ouahed *et al*, 2020). These monogenic mutations disrupt immunologic cascades and lead to defects in cell signaling, mucosal barrier integrity, and inflammation regulation(Ouahed, 2022). The median age at PIBD onset in the entire cohort was 6 years; however, both $PIBD^{Mono}$ and $PIBD^{GIS}$ patients were diagnosed at significantly younger ages than the remaining patients (designated $PIBD^{WT}$) (Fig. 7B and Fig. EV7B).

CD and unclassified IBD were the most common diagnosis (Fig. EV7C). Clinically, $PIBD^{GIS}$ patients were more likely to be therapy-refractory, require surgical intervention, and receive combination therapies compared to $PIBD^{WT}$ patients, suggesting that genomic instability may be associated with refractory IBD (Fig. 7, C and D).

We have incorporated these clarifications into the revised Results section to ensure that the term's usage is clear to readers.

Comment 7. Please ensure that sample size, number of biological replicates, statistical tests,

and the meaning of error bars are reported consistently for the key in vivo, patient-derived, and mechanistic experiments.

Response: Thank you for reminding us of this important point. We have revised the manuscript to ensure that sample sizes, numbers of biological replicates, statistical tests, and the meaning of error bars are reported consistently across all experiments, including in vivo studies, patient-derived sample analyses, and mechanistic assays. The changes are highlighted in main text.

Comment 8. There are several grammatical issues and awkward constructions in the Results and Discussion sections, and some sentences could be revised for clarity and precision.

Response: We have carefully revised the manuscript to improve clarity and precision.

Overall, I recommend major revision. The study is of interest, and the main issues relate primarily to framing, interpretation, and consistency of reporting.

Response: Thank you for your assessment. We have addressed all major concerns regarding framing, interpretation, and consistency of reporting as detailed in our responses to Comments 1-8. We hope the revised manuscript is now suitable for publication.

Referee #2 (Remarks for Author):

This study identifies RADX as an important regulator of intestinal homeostasis, protecting against inflammation by limiting the detection of single stranded DNA (ssDNA) by the innate immune sensor IFI16. The authors show that loss of RADX, particularly in hematopoietic cells, results in ssDNA accumulation, leading to excessive activation of the NF κB pathway and inflammasome signaling. Using mouse models of colitis, biochemical approaches, and data from a pediatric inflammatory bowel disease (PIBD) cohort, they further demonstrate that rare RADX variants reduce protein stability and enhance pro inflammatory responses. Notably, pharmacological inhibition of RAD51 with RI 1 reduces DNA damage and improves disease severity in colitis models, pointing to a potential therapeutic strategy for a subset of IBD patients with underlying genomic instability.

While the findings are of interest, several important issues require clarification.

Major points

Comment 1. Many experimental conditions are not described with sufficient clarity, which limits the reader's ability to fully understand the results and assess the authors' interpretations.

Response: Thank you for the reminding us of this important point. We have carefully reviewed the manuscript and supplemented the detail experimental conditions in the Materials and Methods section, as well as in the corresponding figure legends. These include, but are not limited to, incubation times, treatment concentrations, cell culture conditions, and animal procedures. We believe these conditions now provide readers with sufficient clarity to understand the results and assess our interpretations.

Comment 2. The number of biological replicates is not indicated for most experiments in the figure legends, making it difficult to assess the robustness and reproducibility of the data.

Response: We apologize for the missing information. We have reviewed the manuscript carefully and have now supplemented the number of biological replicates in the corresponding figure legends. We believe this revision improves the robustness and

reproducibility of our data.

Comment 3. Do Radx-/- mice express a truncated RADX protein? are there any basal physiological alterations in these mice, such as changes in body weight, growth rate, or general development?

Response: The RADX antibody used in this study was raised against a recombinant fragment corresponding to amino acids 358-580 of human RADX (nucleotides 1072-1740 of the mRNA). Although we did not detect full-length RADX protein in colonic tissue from *Radx*$^{-/-}$ mice, we cannot exclude the possibility of a truncated RADX protein, as the epitope recognized by this antibody may be absent, as the high conservation between human and mouse RADX (**Response Fig. 2A-B**).

The mouse *Radx* gene (GenBank accession number: NM_175326.6; Ensembl: ENSMUSG00000042498) is located on the X chromosome and contains 14 exons. Guide RNAs (gRNAs) were designed to delete exon 4 to exon 9. The gRNA targeting *Radx* gene, and Cas9 mRNA were co-injected into fertilized mouse eggs to generate targeted knockout offspring (**Response Fig. 2C**).

Mapping to the mRNA sequence, nucleotides 980-1734 (a 755-nt region) are removed from the *Radx* mRNA. This deletion causes a frameshift and introduces a premature termination codon at nucleotides 985-987 (**Response Fig. 2C**). Nonsense-mediated mRNA decay (NMD) is a quality control mechanism that targets mRNAs with truncated open reading frames arising from premature termination codon(Karousis *et al*, 2016). When a termination codon is positioned distant from the poly(A) tail, translation termination is aberrant and promotes ERF3-UFP1 interaction, leading to UPF1 phosphorylation and RNA degradation(Karousis *et al.*, 2016). Theoretically, the truncated *Radx* mRNA in *Radx*$^{-/-}$ would be subjected to NMD.

To test whether our knockout allele produces a stable truncated transcript, we performed real-time PCR on tissue samples from WT and *Radx*$^{-/-}$ mice using two primer pairs. Primer pair 1 amplifies nucleotides 769-850 (present in both transcripts), and primer pair 2 amplifies nucleotides 1316-1417 (within the deleted region). Both two primers showed significant

reduced levels of *Radx* mRNA in *Radx*$^{-/-}$ mice, indicating that the truncated *Radx* transcript is unstable and likely degraded by NMD (**Response Fig. 2D**).

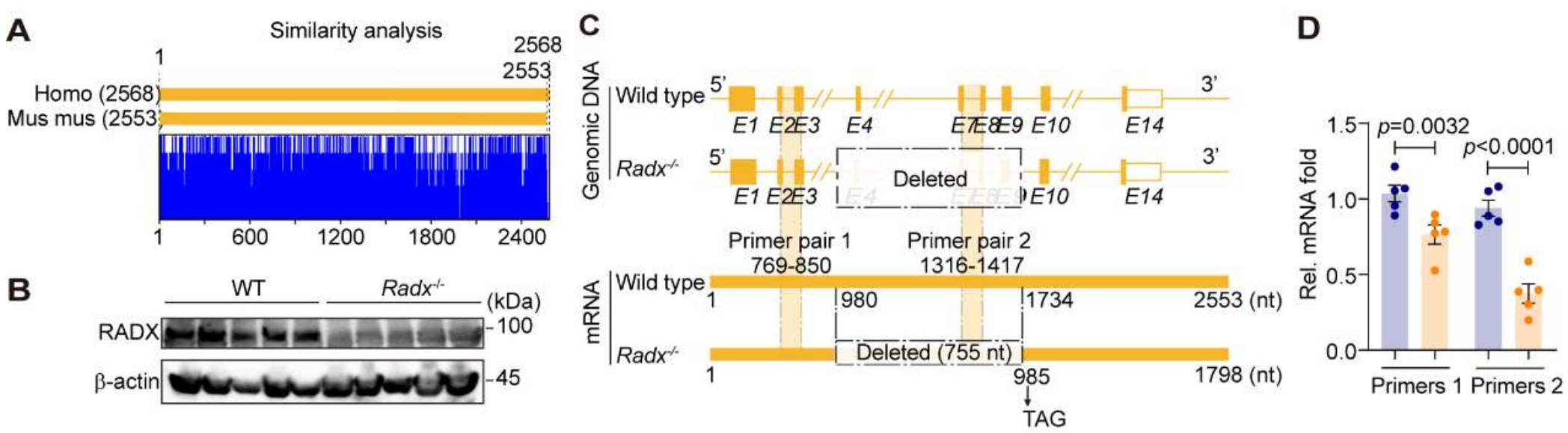

Response Fig. 2| The truncated *Radx* transcript is unstable and likely degraded by NMD. (**A**) Similarity analysis of RADX coding sequence from *Homo sapiens* (human) and *Mus musculus* (house mouse). (**B**) Immunoblotting analysis of RADX protein levels in colonic tissues from WT and *Radx*$^{-/-}$ mice. n=5 for each group. (**C**) Schematic of *Radx* knockout mouse strategy. (**D**) Real-time PCR analysis of tail biopsies from WT and *Radx*$^{-/-}$ mice with primer pair 1 and primer pair 2, respectively. n=5. Data were presented as mean± SEM and analyzed using one-way ANOVA followed by Tukey-Kramer multiple-comparisons test.

To assess whether *Radx*$^{-/-}$ mice display basal physiological alterations, we monitored body weight change, colon length and colon histology in WT and *Radx*$^{-/-}$ male mice. *Radx*$^{-/-}$ mice showed significant lower body weight change and shorter colon length compared with WT littermates (sacrifice at 45 weeks of age) (**Response Fig. 3A-B**). In addition, we observed significantly increased histological scores, immune cell infiltration (CD45^{+}), IL-1β signal, and fibrosis in distal colon of *Radx*$^{-/-}$ mice, but not the proximal colon (**Response Fig. 3C-J**).

Figure for reviewers removed

Comment 4. In Figure 1H, the sample number does not appear to correspond to that shown in Figure 1F. This discrepancy could affect the statistical analysis. Could the authors clarify this point?

Response: Thank you for raising this important point. In our initial analysis, the disease activity index (DAI), colon length, and histological score (Fig. 1A, 1B, 1F) were assessed for all mice in the DSS-induced colitis model ($n_{WT\text{-}Ctrl}$=10, $n_{WT\text{-}DSS}$=9, $n_{Radx\text{-/-}\text{-}Ctrl}$=8, $n_{Radx\text{-/-}\text{-}DSS}$=8). However, for real-time PCR (Fig. 1C), fecal LCN2 (Fig. 1D), and immunofluorescence staining (Fig. 1H), we randomly selected 4-6 individuals per group. We acknowledge that this discrepancy could affect the interpretation.

To address your concern, we have now performed the missing assays on all remaining samples and included the complete dataset in the revised manuscript. The updated figures show consistent results, and the statistical analyses now correspond fully to the sample sizes shown in Figure 1F. We have also clarified this in the figure legends.

We apologize for any confusion and thank you for your careful review.

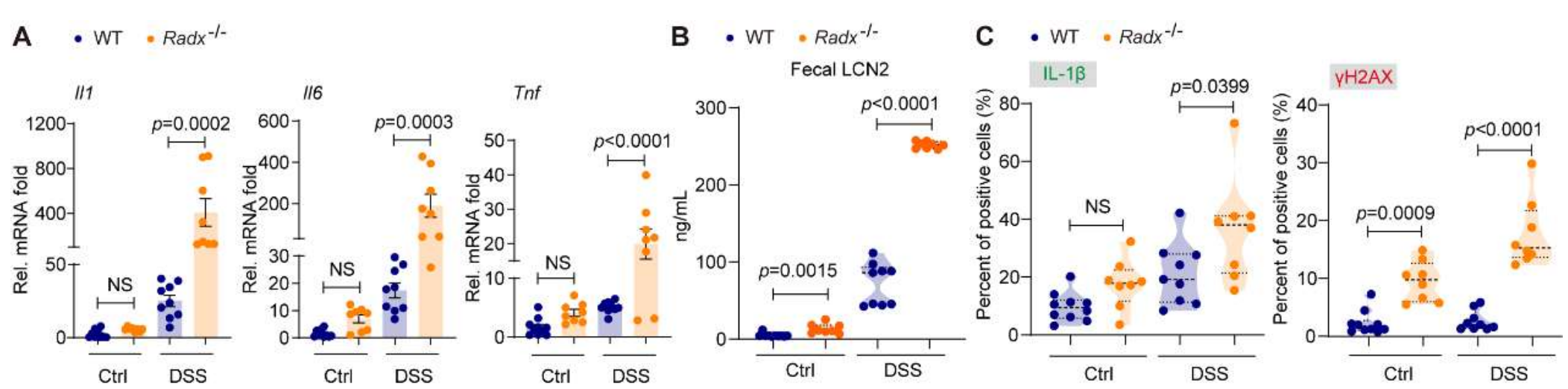

Response Fig. 4| *Radx* deficiency aggravates DSS-induced acute colitis. (**A**) Real-time PCR of colonic homogenates of WT or *Radx*$^{-/-}$ mice from DSS-induced acute colitis model. n=10 in WT-Ctrl group, n=9 in WT-DSS group, n=8 in *Radx*$^{-/-}$-Ctrl group, and n=8 in *Radx*$^{-/-}$-DSS group of two independent experiments. (**B**) Fecal LCN2 detection of WT or *Radx*$^{-/-}$ mice in DSS-induced acute colitis model. (**C**) Quantification of immunofluorescence staining of distal colon segments from WT or *Radx*$^{-/-}$ mice in DSS-induced acute colitis model. Data were presented as mean±SEM and analyzed using one-way ANOVA followed by Tukey-Kramer multiple-comparisons test.

Comment 5. The authors should clearly describe the mouse treatment protocol, including the timing and duration of DSS administration and the procedures used to collect the final experimental readouts.

Response: We apologize for the omission. We have carefully reviewed the manuscript and supplemented the detailed protocols of all experiments in this study. The DSS-induced acute colitis model as originally described as follows:

For DSS-induced acute colitis model, mice were administered 3% DSS (36-50 kDa; MP Biomedicals) in their drinking water ad libitum for 7 consecutive days, followed by 2 days of normal drinking water. Control mice were administered with normal drinking water throughout the duration of the experiment. Weight loss, rectal bleeding and diarrhea were monitored daily at the same time. The DSS water was replaced on days 3 and 5.

Specifically, we have added the following information:

Mice were sacrificed on day 9. Fecal samples were collected and immediately placed on the dry ice for subsequent LCN2 detection. After photographing the whole colon, rings of distal and proximal colon were fixed in 4% paraformaldehyde for subsequent H&E and immunofluorescence staining. The remaining colon tissues were collected in RNAlater (Invitrogen, AM7021) for the RNA extraction and real-time PCR analysis.

▪ ***Disease activity index (DAI) criteria(Wirtz et al., 2017)***

Score	*Weight loss (%)*	*Stool consistency*	*Blood in stool*
0	*0*	*Normal*	*Negative hemocult*
1	*1-5*	*Soft but still formed*	*Negative hemocult*
2	*6-10*	*Soft*	*Positive hemocult*
3	*11-18*	*Very soft; wet*	*Blood traces in stool visible*
4	*>18*	*Watery diarrhea*	*Gross rectal bleeding*

▪ ***Scoring system for DSS-induced histological changes in the colon(Wirtz et al., 2017)***

Score	*Tissue damage*	*Lamina propria inflammatory cell infiltration*
0	*None*	*Infrequent*
1	*Isolated focal epithelial damage*	*Increased, some neutrophils*
2	*Mucosal erosions and ulcerations*	*Submucosal presence of inflammatory cell clusters*
3	*Extensive damage deep into the bowel wall*	*Transmural cell infiltrations*

- ***Scoring system for TNBS-induced histological changes in the colon*** *(Wirtz et al., 2017)*

Score	*Histologic changes in TNBS colitis*
0	*No evidence of inflammation*
1	*Low level of inflammation, with scattered infiltrating mononuclear cells (1–2 foci)*
2	*Moderate inflammation, with multiple foci*
3	*High level of inflammation, with increased vascular density and marked wall thickening*
4	*Maximal severity of inflammation, with transmural leukocyte infiltration and loss of goblet cells*

Comment 6. It is unclear why the authors did not directly conclude that macrophages play a major role in RADX mediated colitis, rather than broadly attributing the effect to hematopoietic cells. This distinction is particularly important given that dendritic cells do not appear to exhibit markers of genomic instability or secrete IL 1β in this study.

Response: Thank you for raising this important point. Our data support a major contribution from the hematopoietic compartment, particularly macrophages, to the exacerbated colitis phenotype in *Radx*$^{-/-}$ mice, as evidenced by bone marrow transplantation, RADX expression profiling in different cell types, and macrophage functional assays. Although we observed low

RADX expression level in dendritic cells, we cannot exclude the contributions from other cell types at this stage. Nevertheless, we agree that clearly attributing the effect to macrophages is important for the interpretation of our study. Therefore, we have revised the statement that macrophages play a major role in RADX-mediated colitis, while noting that additional cellular contributions are not entirely excluded.

Comment 7. The bone marrow transplantation procedure described in Figure 2 is not clearly explained, either in the main text or in the Materials and Methods section. Specifically, it is unclear whether the recipient mice underwent bone marrow ablation prior to transplantation. If not, the authors should explain how the donor (KO) bone marrow was able to effectively reconstitute or outcompete the endogenous bone marrow of the WT recipients.

Response: Response: We apologize for the omission. We have now supplemented the detailed bone marrow transplantation protocol in the Materials and Methods section. Specifically:

Radx$^{-/-}$ mice and WT littermates (males, 8 weeks old) were used as donors of bone marrow cells. Bone marrow was isolated from the femurs of hind legs. Following red blood cell lysis, the cell suspension was strained through a 70 μm cell strainer. The cell pellets were washed three times with sterile PBS, counted, and stored on ice for subsequent injection. Recipient Radx$^{-/-}$ mice and WT littermates (males, 8 weeks old) were lethally irradiated with a total dose of 8.0 Gy (1.0Gy/min) and transplanted with 1×107 bone marrow cells via ophthalmic vein injection. Mice were housed in specific-pathogen free facilities and provided with drinking water supplemented with gentamicin (1 mg/mL) for 7 days. Thereafter, they received sterilized water and standard chow ad libitum for 6 weeks to reconstitute the immune system before being subjected to the colitis model.

Thus, the endogenous bone marrow of recipient mice was ablated by lethal irradiation, and the donor-derived cells reconstituted the hematopoietic system. We thank you for pointing out this lack of clarity and have now made the protocol explicit in the revised manuscript.

Comment 8. In Fig. S3C, the authors use the Tert gene to assess the presence of nuclear DNA

in the cytosol. It is unclear why Tert was chosen as a representative marker. How can the authors be certain that Tert specifically, or preferentially, would be released into the cytosol following DNA damage? Additional justification or validation of this approach would be helpful.

Response: The primary goal of our experiment in Fig. EV3C was to quantify the overall level of genomic DNA (gDNA) that had leaked into cytoplasm. We chose the *TERT* gene merely as a representative marker for nuclear DNA, not because we assumed it to be specifically or preferentially released following DNA damage. As DNA damage-induced gDNA leakage is a genome-wide and stochastic process, the abundance of any single-copy nuclear gene should reflect the total nuclear DNA content in the cytosolic.

TERT was selected for its practical advantages: it is a stable, single-copy nuclear gene(Kuhns *et al*, 2019) (Chromosome 5: 1253147-1295085) without known processed pseudogenes in the mitochondrial genome, which minimizes the risk of co-amplifying mitochondrial DNA (mtDNA). Our aim was not to link the TERT locus specifically to DNA damage response, but to use it as a general proxy to measure cytosolic nuclear DNA levels.

This approach of using representative nuclear gene (e.g., *TERT*) as a real-time PCR marker for cytosolic nuclear DNA is widely established in the field:

1. West, A.P., et al. (2015). Nature 520, 553-557. 10.1038/nature14156.
   In the METHOD section, "*Mitochondrial DNA copy number analysis was performed as described using primers specific to nuclear Tert and the D-loop region of mtDNA*" (West *et al*, 2015). Furthermore, the protocol notes that "*No nuclear Tert DNA was detected in the cytosolic fractions, indicating nuclear lysis did not occur*" confirming the specificity of *TERT* as a nuclear marker.
2. Enzan, N., et al. (2023). Circ Res 132, 1110-1126.
   10.1161/CIRCRESAHA.122.322227.
   In the RESULTS section, "*Dloop, a component of mtDNA, but not Tert and B2m, components of nuclear DNA, was increased in cytosolic fraction from noninfarcted*

region of mouse hearts after myocardial infarction compared with control hearts" (Enzan *et al*, 2023).

3. Lin, M., et al. (2023). Nat Cardiovasc Res 2, 778-792. 10.1038/s44161-023-00314-x. In the Results and Methods sections "*In contrast, cytosolic nuclear DNA accumulation was not observed in Mcm8$^{-/-}$ mice or patients with KD*" and "*Quantitative PCR was performed using nuclear DNA primers (Tert) and mtDNA primers (DLOOP)*" (Lin *et al*, 2023).

These studies demonstrate that *TERT* is routinely and reliably used as a nuclear DNA marker to detect cytosolic leakage of genomic DNA. We hope this clarifies our rationale.

Comment 9. In Figure 4A, the number of biological replicates is not indicated. Additionally, if IFI16 is proposed as a key regulator of this pathway, one would expect a more pronounced reduction in p p65 levels in siIFI16 treated cells, similar to the effect observed with siSTING in Figure S4A. The authors should clarify this discrepancy and discuss the relative contribution of IFI16 to NF κB activation.

Response: Thank you for remaindering these points.

First, we apologize for omitting the number of biological replicates for Figure 4A. All immunoblotting assays were repeated at least three times with independent biological replicates, and we have added this information to the figure legend.

Second, regarding the differential effects of IFI16 and STING knockdown on p-p65 levels: we agree that IFI16 knockdown in RADX-deficiency macrophages reduced p-p65 signaling (**Fig. 4A**, *siCtrl*=1.0, *siIFI16&siRADX*=0.9), whereas STING knockdown produced a more pronounced reduction (**Fig. EV4A**, *siCtrl*=1.0, *siSTING&siRADX*=0.5). We interpret this difference as follows:

- IFI16 functions as an upstream DNA sensor that recognizes ssDNA derived from RADX deficiency-induced replication stress. However, upon LPS stimulation, the majority of p-p65 signaling is driven the canonical TLR4 pathway. RADX deficiency exacerbates p-p65 signaling by accumulating ssDNA and enhancing IFI16-ssDNA binding. Therefore,

knockdown IFI16 in RADX-deficiency macrophages reduced the abnormal (exacerbated) component of p-p65 signaling (**Fig. 4A**, *siRADX*=1.6, *siIFI16&siRADX*=0.9) but cannot affect the canonical TLR4-mediated NF-κB activation.

- STING, in contrast, act as a central signaling hub downstream of multiple DNA sensors including cGAS, IFI16 and DDX41(Sun *et al*, 2013; Unterholzner *et al*, 2010; Zhang *et al*, 2011). Consequently, STING knockdown blocks a broader convergence point for NF-κB activation, leading to a more complete inhibition, possibly through additional or unknown mechanisms in canonical TLR4-mediated NF-κB activation.

We have now included this discussion in the revised manuscript to clarify the relative contribution of IFI16 to NF-κB activation in our model.

Comment 10. In the in vitro DNA binding assay (Figure 4D), the working concentrations of RADX and IFI16 are not indicated. In addition, no concentration dependent DNA binding analysis is presented. Including these details and experiments would be important to assess the specificity and strength of the reported interactions.

Response: Thank you for the reminder. In the in vitro DNA binding assay, the incubating concentrations of RADX and IFI16 are 10 micrograms, respectively. We have added this information to revised manuscript.

Furthermore, we have supplemented a concentration dependent DNA binding analysis (**Response Fig.4**). In this assay, we increased the amount of RADX (1 μg, 10 μg, 50 μg) while keeping IFI16 and ssDNA constant. The result demonstrates a concentration-dependent binding interaction, supporting the specificity and strength of RADX in DNA sensing by IFI16. We have also added a brief description of these results in the revised manuscript.

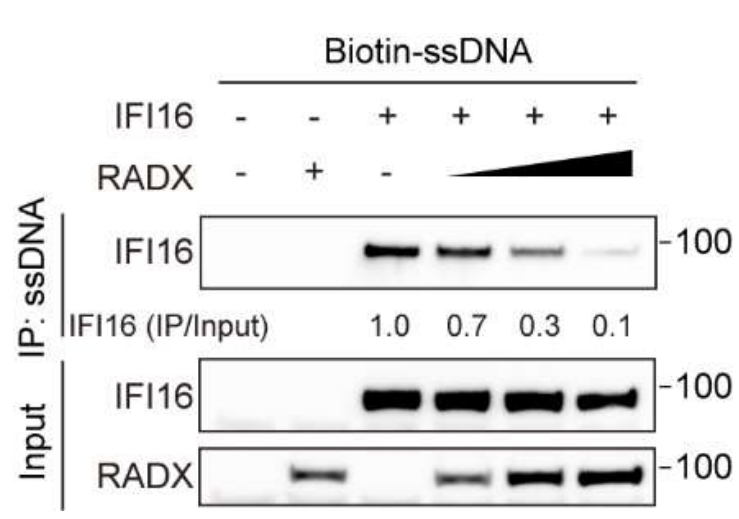

Response Fig. 5| RADX disrupts IFI16-ssDNA binding concentration dependently. In vitro ssDNA binding assay of IFI16 with increasing dose of RADX protein (1 µg, 10 µg, 50 µg). Immunoblotting assays were repeated at least three times with independent biological replicates.

Comment 11. In Figure 4E, it is unclear whether the ssDNA foci are localized in the nucleus or in the cytoplasm. The authors should clarify the subcellular localization of these ssDNA signals.

Response: In the original Figure 4E, we did not show the DAPI channel in order to present the clearer merge image IFI16 and BrdU signal. To address your concern, we have now included the DAPI images together with IFI16 and BrdU signals in Expanded View Figure. As shown in Response Figure 5, IFI16 is predominantly localized in the nucleus, and the ssDNA foci (labeled by BrdU under non-denaturing conditions) as well. We have updated the figure accordingly and revised the manuscript text to clarify the subcellular localization of these ssDNA foci.

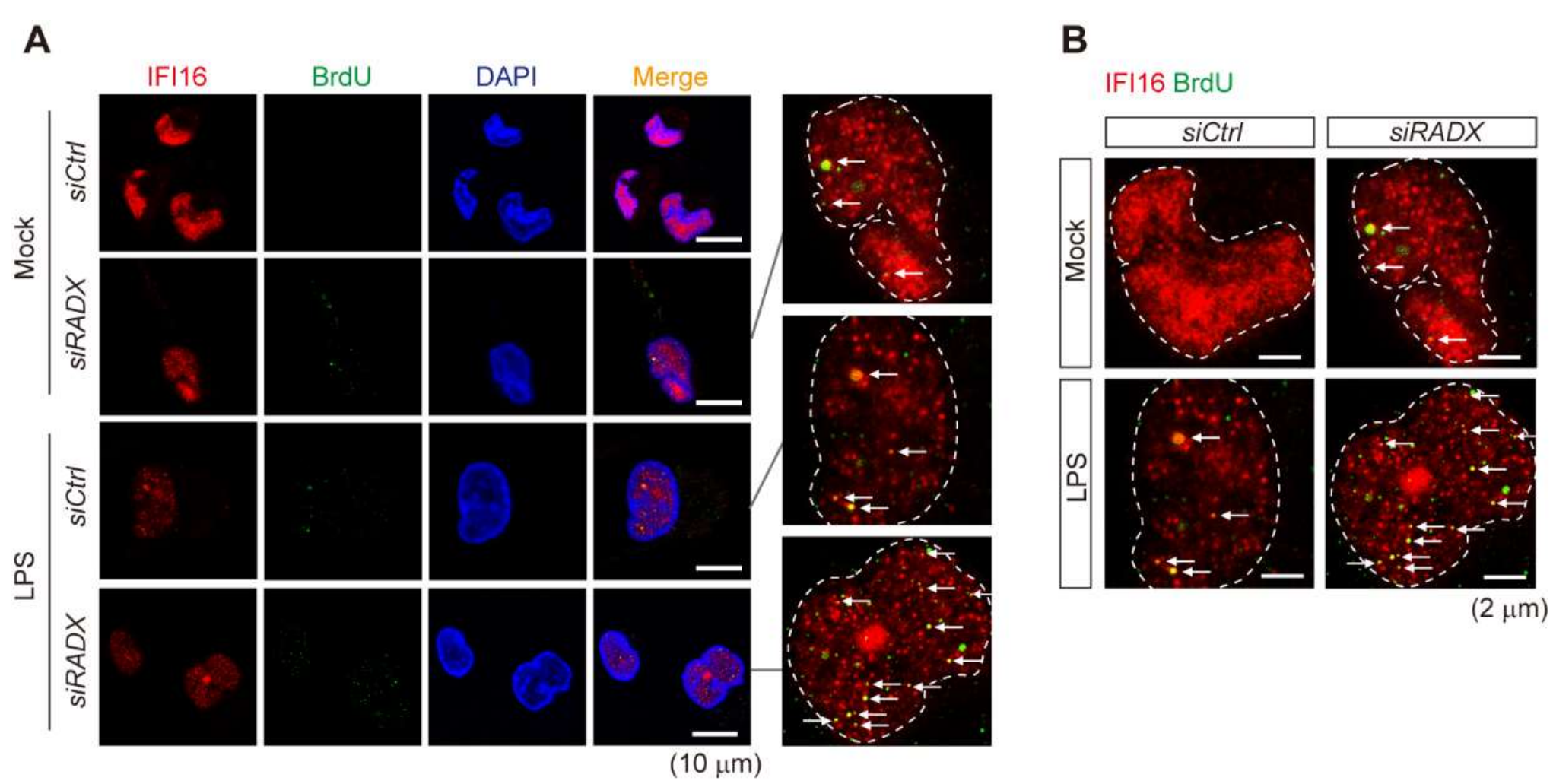

Response Fig. 5| Subcellular localization of ssDNA foci. (**A**) All channels of representative confocal image of macrophages transfected with *siCtrl* or *siRADX* and pre-labelled with BrdU for 48 h. LPS was applied for stimulation for 2 h. The dashed lines represented the position of nuclear membranes. (**B**) IFI16 and BrdU signals were displayed in main text.

Comment 12. The quantification of colocalization between ASC and IFI16 is missing.

Response: Thank you for the reminder. We have added the quantification of colocalization between ASC and IFI16 (**Response Fig. 6**). Compared with control macrophages, RADX-deficiency macrophages exhibited increased ASC foci under basal condition and LPS treatment. These results suggest that RADX deficiency enhances ASC-IFI16 interaction. The corresponding quantification has been included in the figure, and the statistical analysis is described in the figure legend.

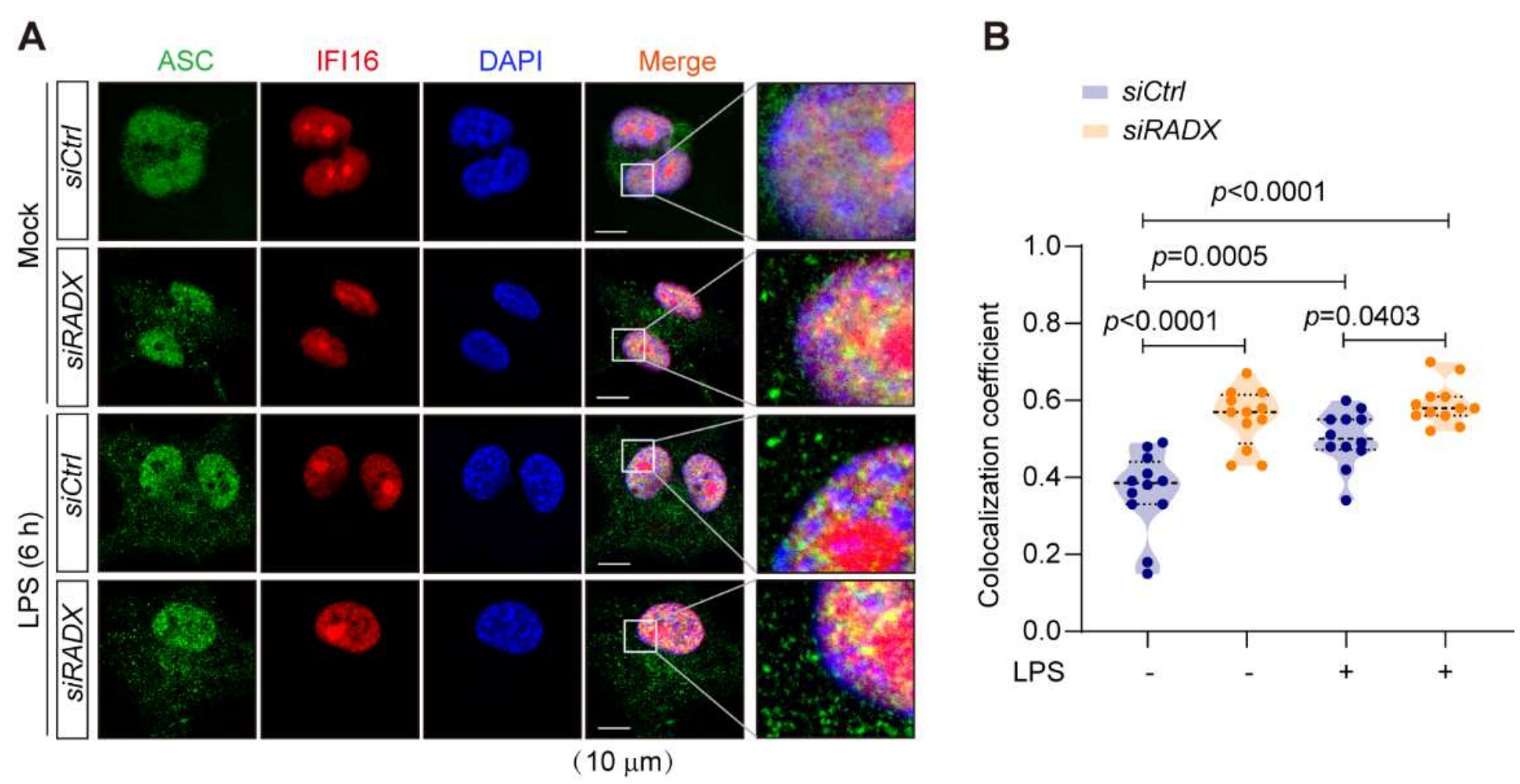

Response Fig. 6| Quantification of colocalization between ASC and IFI16. (**A** and **B**) Representative images (A) and statistical analysis (B) of immunofluorescence of *siCtrl* or *siRADX* transfected macrophages treated with LPS for 6h with confocal microscope. Each data point represents a measurement from independent cell. n=12 for each group. Data were presented as mean±SEM and analyzed using one-way ANOVA followed by Tukey-Kramer multiple-comparisons test.

Comment 13. Authors should provide evidence showing STING localization in the nucleus.

Response: By immunoblotting cytosolic (Cyto) and nuclear (Nuc) fractions, we observed increased STING abundance upon LPS treatment in nucleus of control macrophages, whereas RADX deficiency significantly increased nuclear-localized STING (**Response Fig. 7A**). Following DNA stimulation, STING is recruited to and interacts with IFI16, triggering

downstream signaling(Unterholzner *et al.*, 2010). To further confirm the STING-IFI16 interaction, we performed a Proximity Ligation Assay (PLA) and found that RADX deficiency significantly increased STING-IFI16 interaction in nucleus (**Response Fig. 7B**).

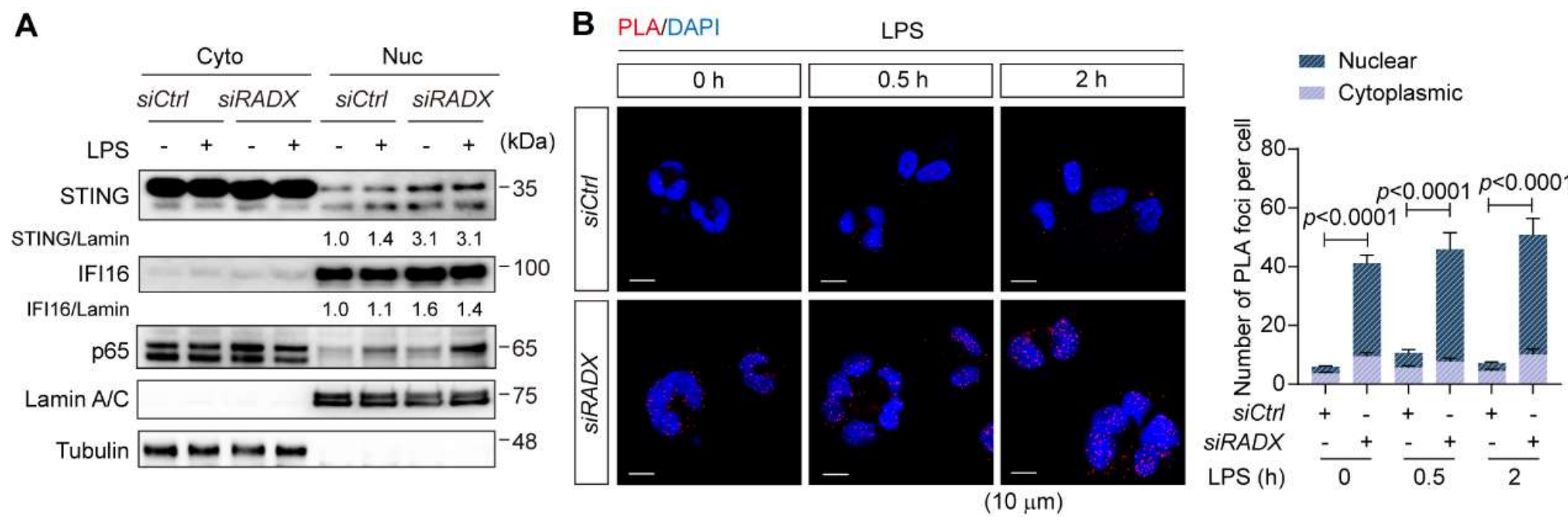

Response Fig. 7| RADX deficiency increases STING-IFI16 interaction predominantly in the nucleus. (**A**) Immunoblotting analysis of cytosolic (Cyto) and nuclear (Nuc) fractions from *siCtrl* or *siRADX* transfected macrophages treated with LPS for 30 min. (**B**) Proximity Ligation Assay (PLA) of *siCtrl* or *siRADX* transfected macrophages treated with LPS for indicated time. Red foci indicate IFI16-STING interaction. Each data point represents a measurement from independent cell. n=21-37. Immunoblotting assays were repeated at least three times with independent biological replicates. Data were presented as mean±SEM and analyzed using one-way ANOVA followed by Tukey-Kramer multiple-comparisons test.

Minor points

Comment 14. The labels for some histological and immunofluorescence markers, particularly those shown in blue, green, and red on a black background, are difficult to read, which affects figure clarity.

Response: Thank you for your reminder. We have removed the black background and confirmed that all labels are now clearly legible.

Comment 15. In Figure 3H (IL 1β secretion), is the difference between control and LPS stimulated WT BMDMs not statistically significant?

Response: We have checked all the significance analyses and confirmed that the p value for

the IL-1β comparison between control and LPS-treated WT BMDMs is 0.0007, indicating a highly significant difference. We apologize if the original figure annotation caused any confusion and have corrected it accordingly.

Comment 16. In Figure 5F, are the differences observed for IL 1β following 12 hour LPS treatment and for TNF α following 24 hour LPS treatment not statistically significant?

Response: We have checked all the significance analyses and confirmed that the p values for IL 1β following 12-hour LPS treatment and for TNF-α following 24-hour LPS treatment are both less than 0.0001, indicating a highly significant difference. We apologize if the original figure annotation caused any confusion and have corrected it accordingly.

References

Enzan N, Matsushima S, Ikeda S, Okabe K, Ishikita A, Yamamoto T, Sada M, Miyake R, Tsutsui Y, Nishimura R *et al* (2023) ZBP1 Protects Against mtDNA-Induced Myocardial Inflammation in Failing Hearts. *Circ Res* 132: 1110-1126

Hartwig O, Shetab Boushehri MA, Shalaby KS, Loretz B, Lamprecht A, Lehr CM (2021) Drug delivery to the inflamed intestinal mucosa - targeting technologies and human cell culture models for better therapies of IBD. *Adv Drug Deliv Rev* 175: 113828

Karousis ED, Nasif S, Muhlemann O (2016) Nonsense-mediated mRNA decay: novel mechanistic insights and biological impact. *Wiley Interdiscip Rev RNA* 7: 661-682

Konys C, Usman Y, Runde J (2025) From Genes to Gut: Understanding Diagnosis and Treatment in Very Early Onset Inflammatory Bowel Disease. *Pediatr Ann* 54: e280-e285

Kuhns DB, Hsu AP, Sun D, Lau K, Fink D, Griffith P, Huang DW, Priel DAL, Mendez L, Kreuzburg S *et al* (2019) NCF1 (p47(phox))-deficient chronic granulomatous disease: comprehensive genetic and flow cytometric analysis. *Blood Adv* 3: 136-147

Lin M, Xian H, Chen Z, Wang S, Liu M, Liang W, Tang Q, Liu Y, Huang W, Che D *et al* (2023) MCM8-mediated mitophagy protects vascular health in response to nitric oxide signaling in a mouse model of Kawasaki disease. *Nat Cardiovasc Res* 2: 778-792

Liu JZ, van Sommeren S, Huang H, Ng SC, Alberts R, Takahashi A, Ripke S, Lee JC, Jostins L, Shah T *et al* (2015) Association analyses identify 38 susceptibility loci for inflammatory bowel disease and highlight shared genetic risk across populations. *Nat Genet* 47: 979-986

Na YR, Stakenborg M, Seok SH, Matteoli G (2019) Macrophages in intestinal inflammation and resolution: a potential therapeutic target in IBD. *Nat Rev Gastroenterol Hepatol* 16: 531-543

Ouahed J, Spencer E, Kotlarz D, Shouval DS, Kowalik M, Peng K, Field M, Grushkin-Lerner L, Pai SY, Bousvaros A *et al* (2020) Very Early Onset Inflammatory Bowel Disease: A Clinical Approach With a Focus on the Role of Genetics and Underlying Immune Deficiencies. *Inflamm Bowel Dis* 26: 820-842

Ouahed JD (2022) Understanding inborn errors of immunity: A lens into the pathophysiology of monogenic inflammatory bowel disease. *Front Immunol* 13: 1026511

Sun L, Wu J, Du F, Chen X, Chen ZJ (2013) Cyclic GMP-AMP synthase is a cytosolic DNA sensor that activates the type I interferon pathway. *Science* 339: 786-791

Unterholzner L, Keating SE, Baran M, Horan KA, Jensen SB, Sharma S, Sirois CM, Jin T, Latz E, Xiao TS *et al* (2010) IFI16 is an innate immune sensor for intracellular DNA. *Nat Immunol* 11: 997-1004

Valatas V, Vakas M, Kolios G (2013) The value of experimental models of colitis in predicting efficacy of biological therapies for inflammatory bowel diseases. *Am J Physiol*

Gastrointest Liver Physiol 305: G763-785

West AP, Khoury-Hanold W, Staron M, Tal MC, Pineda CM, Lang SM, Bestwick M, Duguay BA, Raimundo N, MacDuff DA *et al* (2015) Mitochondrial DNA stress primes the antiviral innate immune response. *Nature* 520: 553-557

Wirtz S, Popp V, Kindermann M, Gerlach K, Weigmann B, Fichtner-Feigl S, Neurath MF (2017) Chemically induced mouse models of acute and chronic intestinal inflammation. *Nat Protoc* 12: 1295-1309

Zhang Z, Yuan B, Bao M, Lu N, Kim T, Liu YJ (2011) The helicase DDX41 senses intracellular DNA mediated by the adaptor STING in dendritic cells. *Nat Immunol* 12: 959-965

7th Jul 2026

Dear Prof. Zhang,

Thank you for submitting your revised manuscript to EMBO Molecular Medicine. I am pleased to inform you that we will be able to accept your manuscript pending the following final amendments:

1) Authors: Please provide an institutional email address for co-corresponding author Lanlan Geng.
2) Figures: We note that some graphs are reused e.g. Figure 4E and Figure EV4E. Please cite in the respective figure legend every reused panel.
3) Please consider revising the title to better emphasize the main findings, e.g. "RADX protects against inflammatory bowel disease by suppressing IFI16-mediated innate immunity".
4) Abstract: I have gone through your text and revised it (see below). Please review it and amend as you see fit:

Genomic instability is increased in patients with inflammatory bowel disease (IBD), yet whether it contributes directly to disease pathogenesis remains unclear. Here, we identify RADX, a structural antagonist to RAD51 and a key regulator of replication fork stability, as a critical suppressor of intestinal inflammation by limiting innate immune sensing of replication-associated DNA damage. RADX deficiency exacerbates experimental colitis, with macrophages serving as the principal mediators of this phenotype. Mechanistically, RADX competes with the DNA sensor IFI16 for binding to single-stranded DNA (ssDNA). Loss of RADX promotes ssDNA accumulation, triggering IFI16-dependent activation of NF-κB signaling and inflammasome assembly, thereby driving intestinal inflammation. Consistent with these findings, two RADX variants identified in patients with IBD associate with reduced RADX protein expression, increased DNA damage signaling, and elevated IL-1β levels. Pharmacological inhibition of RAD51 with RI-1 alleviated colitis in both wild-type and Radx-deficient mice. Together, these findings establish a mechanistic link between genome instability and intestinal inflammation, identify a RADX-IFI16 checkpoint that restrains pathogenic innate immune activation, and nominate modulation of replication stress as a therapeutic strategy for IBD.

5) In the main manuscript file, please do the following:
- Please address all comments suggested by our data editors listed below:
o Figure legends:
1. Please note that the exact p values are not provided in the legends of figures 1A, C, D, H; 2E, I; 3B, D, H; 4B, G, H, K, L; 5E, F, J; 6B, D, E, 7E, G; EV1 I, EV2 C, EV3 C, EV6 B, D, E.
2. Please note that the box plots need to be defined in terms of minima, maxima, centre, bounds of box and whiskers, and percentile in the legends of figures 1F, J, L; 7E, EV1 G.
- Remove the One Sentence Summary from the title page.
- The manuscript sections should be in the following order: Title page - Abstract - Introduction - Results - Discussion - Methods - Data Availability - Acknowledgments - Disclosure Statement & Competing Interests - References - Figure Legends - (Main Tables with legends if applicable) - Expanded View Figure Legends.
- In Methods, provide the antibody dilutions that were used for each antibody.
- In Methods, information about sample size, randomization and blinding should be part of the Statistics paragraph.
- In Methods, provide the statement that in addition to the WMA Declaration of Helsinki the experiments involving human subjects also conformed to the principles set out in the Department of Health and Human Services Belmont Report.
- Please add the expanded view figure legends to the manuscript text.
- Indicate in legends number and nature of replicates and exact p= values, not a range, along with the statistical test used. To keep the figures "clear" some authors found providing an Appendix table Sx with all exact p-values preferable. You are welcome to do this if you want to.
- Correct the reference citation in the reference list. Where there are more than 10 authors on a paper, 10 will be listed, followed by "et al.". Please check "Author Guidelines" for more information. https://www.embopress.org/page/journal/17574684/authorguide#referencesformat
- In data availability statement please remove provided text and add the sentence "This study includes no data deposited in external repositories."
6) Funding: Remove the "funding" heading from the Acknowledgments and make sure that information about all sources of funding are complete in both our submission system and in the manuscript. Currently, Guangzhou National Laboratory and State Key Laboratory of Respiratory Disease grant GZNL2024B01002 (Y.Z.), Major Project of Guangzhou National Laboratory grant GZNL2023A01009 (Y.Z.), Guangzhou Key Research and Development Program grant 202206010002 (Y.Z.), Guangzhou Science and Technology Planning Project grant 2220078 (Y.Z.), Guangzhou Women and Children's Medical Center are missing in our submission system. Please correct.
7) Tables: Please rename Table EV2 to Dataset EV1 and update its callouts in the main text.
8) Synopsis:
- Synopsis image: Please resize the image to exactly 550 px-wide x 300 pixels high and use larger font for the text whewre appropriate. Upload it as a high resolution .jpeg/.png file.
- Please check your synopsis text and image before submission with your revised manuscript. Please be aware that in the proof stage minor corrections only are allowed (e.g., typos).

9) As part of the EMBO Publications transparent editorial process (see our Editorial at http://embomolmed.embopress.org/content/2/9/329), EMBO Molecular Medicine will publish online a Review Process File (RPF) to accompany accepted manuscripts. This file will be published in conjunction with your paper and will include the anonymous referee reports, your point-by-point response and all pertinent correspondence relating to the manuscript. Let us know if you want to remove or not any figures from it prior to publication. Please note that the Authors checklist will be published at the end of the RPF.
10) Please provide a point-by-point letter INCLUDING my comments as well as the reviewer's reports and your detailed responses (as Word file).

I look forward to reading a new revised version of your manuscript as soon as possible.

Yours sincerely,

Zeljko Durdevic

Zeljko Durdevic
Senior Editor
EMBO Molecular Medicine

*** Instructions to submit your revised manuscript ***

*** PLEASE NOTE *** As part of the EMBO Publications transparent editorial process initiative (see our Editorial at https://www.embopress.org/doi/pdf/10.1002/emmm.201000094), EMBO Molecular Medicine will publish online a Review Process File to accompany accepted manuscripts.

In the event of acceptance, this file will be published in conjunction with your paper and will include the anonymous referee reports, your point-by-point response and all pertinent correspondence relating to the manuscript. If you do NOT want this file to be published, please inform the editorial office at contact@embomolmed.org.

*****

When preparing your revised manuscript, please refer to our guidelines: https://link.springer.com/journal/44321/submission-guidelines#cms-Revised-submissions. We perform an initial quality control of all revised manuscripts before re-review; failure to include requested items will delay the evaluation of your revision.

We require:

1) A .docx formatted version of the manuscript text (including legends for main figures, EV figures and tables). Please make sure that the changes are highlighted to be clearly visible.

2) Individual production quality figure files as .eps, .tif, .jpg (one file per figure). For guidance, download the 'Figure Guide PDF': https://media.springernature.com/original/springer-cms/rest/v1/content/27825798/data/v1.

3) A .docx formatted letter INCLUDING the reviewers' reports and your detailed point-by-point responses to their comments. As part of the EMBO Press transparent editorial process, the point-by-point response is part of the Review Process File (RPF), which will be published alongside your paper.

4) A complete author checklist, which you can download from our author guidelines. Please insert information in the checklist that is also reflected in the manuscript. The completed author checklist will also be part of the RPF.

5) Please note that all corresponding authors are required to supply an ORCID ID for their name upon submission of a revised manuscript.

6) It is mandatory to include a 'Data Availability' section after the Materials and Methods. Before submitting your revision, primary

datasets produced in this study need to be deposited in an appropriate public database, and the accession numbers and database listed under 'Data Availability'. Please remember to provide a reviewer password if the datasets are not yet public.

In case you have no data that requires deposition in a public database, please state so in this section. Note that the Data Availability Section is restricted to new primary data that are part of this study.

7) For data quantification: please specify the name of the statistical test used to generate error bars and P values, the number (n) of independent experiments (specify technical or biological replicates) underlying each data point and the test used to calculate p-values in each figure legend. The figure legends should contain a basic description of n, P and the test applied. Graphs must include a description of the bars and the error bars (s.d., s.e.m.).

8) At EMBO Press we ask authors to provide source data for the main manuscript figures. You will receive a separate email with instructions for providing source data with your revised manuscript, including how to upload and organize the files.

9) Our journal encourages inclusion of *data citations in the reference list* to directly cite datasets that were re-used and obtained from public databases. Data citations in the article text are distinct from normal bibliographical citations and should directly link to the database records from which the data can be accessed. In the main text, data citations are formatted as follows: "Data ref: Smith et al, 2001" or "Data ref: NCBI Sequence Read Archive PRJNA342805, 2017". In the Reference list, data citations must be labeled with "[DATASET]". A data reference must provide the database name, accession number/identifiers and a resolvable link to the landing page from which the data can be accessed at the end of the reference.

10) We replaced Supplementary Information with Expanded View (EV) Figures and Tables that are collapsible/expandable online. A maximum of 5 EV Figures can be typeset. EV Figures should be cited as 'Figure EV1, Figure EV2" etc... in the text and their respective legends should be included in the main text after the legends of regular figures.

- For the figures that you do NOT wish to display as Expanded View figures, they should be bundled together with their legends in a single PDF file called *Appendix*, which should start with a short Table of Content. Appendix figures should be referred to in the main text as: "Appendix Figure S1, Appendix Figure S2" etc.

- Additional Tables/Datasets should be labeled and referred to as Table EV1, Dataset EV1, etc. Legends have to be provided in a separate tab in case of .xls files. Alternatively, the legend can be supplied as a separate text file (README) and zipped together with the Table/Dataset file.

11) The paper explained: EMBO Molecular Medicine articles are accompanied by a summary of the articles to emphasize the major findings in the paper and their medical implications for the non-specialist reader. Please provide a draft summary of your article highlighting

- the medical issue you are addressing,

- the results obtained and

- their clinical impact.

This may be edited to ensure that readers understand the significance and context of the research. Please refer to any of our published articles for an example.

12) Author contributions: You will be asked to provide CRediT (Contributor Role Taxonomy) terms in the submission system. These replace a narrative author contribution section in the manuscript.

13) A Conflict of Interest statement should be provided in the main text.

14) Every published paper includes a 'Synopsis' to further enhance discoverability. Synopses are displayed on the journal webpage and are freely accessible to all readers. They include a short stand first (maximum of 300 characters, including space) as well as 2-5 one-sentences bullet points that summarizes the paper. Please write the bullet points to summarize the key NEW findings. They should be designed to be complementary to the abstract - i.e. not repeat the same text. We encourage inclusion of key acronyms and quantitative information (maximum of 30 words / bullet point). Please use the passive voice. Please attach these in a separate file or send them by email, we will incorporate them accordingly.

Please also suggest a striking image or visual abstract to illustrate your article as a PNG file 550 px wide x 300 px high.

15) Include a Reagents and Tools Table as part of the Methods section, which can be downloaded from our author guidelines.

EMBO Molecular Medicine has a "scooping protection" policy, whereby similar findings that are published by others during

review or revision are not a criterion for rejection. Should you decide to submit a revised version, I do ask that you get in touch after three months if you have not completed it, to update us on the status.

*Additional important information regarding Figures

Each figure should be given in a separate file and should have the following resolution:
Graphs 800-1,200 DPI
Photos 400-800 DPI
Colour (only CMYK) 300-400 DPI"

Figures are not edited by the production team. All lettering should be the same size and style; figure panels should be indicated by capital letters (A, B, C etc). Gridlines are not allowed except for log plots. Figures should be numbered in the order of their appearance in the text with Arabic numerals. Each Figure must have a separate legend and a caption is needed for each panel.

*Additional important information regarding figures and illustrations can be found at
https://media.springernature.com/original/springer-cms/rest/v1/content/27825798/data/v1

***** Reviewer's comments *****

Referee #1 (Remarks for Author):

The revised manuscript has been significantly improved. The authors have clarified the biological role of RADX in intestinal inflammation and have strengthened the mechanistic link between genomic instability and macrophage-mediated inflammatory responses.

The major strengths of the study include:

Clear mechanistic novelty.
The manuscript identifies RADX as an important regulator of intestinal immune homeostasis and proposes that RADX limits IFI16-mediated sensing of ssDNA, thereby suppressing NF-κB activation and inflammasome assembly.
Strong experimental support across models.
The use of Radx-deficient mice, DSS- and TNBS-induced colitis models, chronic DSS exposure, and bone marrow transplantation provides convincing in vivo evidence that RADX deficiency aggravates intestinal inflammation, with macrophages playing a dominant role.
Clinical relevance.
The identification of RADX variants in pediatric IBD patients and the broader analysis of genomic instability-related variants in the PIBD cohort add translational significance to the study.
Therapeutic implication.
The finding that RI-1 treatment alleviates DNA damage, inflammatory signaling, and colitis severity in both wild-type and Radx-deficient mice provides an interesting therapeutic perspective.

The authors also appropriately acknowledge remaining limitations, including the rarity of RADX variants, the need for larger patient cohorts, and the possibility that other cell types or pathways may contribute to the observed phenotype.
Overall, the revised manuscript is well organized, mechanistically meaningful, and sufficiently supported by experimental evidence.

Referee #2 (Remarks for Author):

The authors have fully taken into account the reviewer's suggestions. The manuscript is now suitable for publication in EMBO Molecular Medicine.

***** Reviewer's comments *****

Referee #1 (Remarks for Author):

The revised manuscript has been significantly improved. The authors have clarified the biological role of RADX in intestinal inflammation and have strengthened the mechanistic link between genomic instability and macrophage-mediated inflammatory responses.

The major strengths of the study include:

Clear mechanistic novelty.

The manuscript identifies RADX as an important regulator of intestinal immune homeostasis and proposes that RADX limits IFI16-mediated sensing of ssDNA, thereby suppressing NF-κB activation and inflammasome assembly.

Strong experimental support across models.

The use of Radx-deficient mice, DSS- and TNBS-induced colitis models, chronic DSS exposure, and bone marrow transplantation provides convincing in vivo evidence that RADX deficiency aggravates intestinal inflammation, with macrophages playing a dominant role.

Clinical relevance.

The identification of RADX variants in pediatric IBD patients and the broader analysis of genomic instability-related variants in the PIBD cohort add translational significance to the study.

Therapeutic implication.

The finding that RI-1 treatment alleviates DNA damage, inflammatory signaling, and colitis severity in both wild-type and Radx-deficient mice provides an interesting therapeutic perspective.

The authors also appropriately acknowledge remaining limitations, including the rarity of RADX variants, the need for larger patient cohorts, and the possibility that other cell types or pathways may contribute to the observed phenotype.

Overall, the revised manuscript is well organized, mechanistically meaningful, and sufficiently supported by experimental evidence.

Response: We sincerely thank the reviewer for the careful evaluation of our revised manuscript and for the positive comments. We appreciate the reviewer's recognition of the novelty, mechanistic insights, and experimental support of our study, as well as the thoughtful comments throughout the review process, which have substantially improved the manuscript. We also appreciate the recognition of the limitations discussed in the manuscript. We thank the reviewer for the constructive comments throughout the review process, which have greatly improved the quality and clarity of our work.

Referee #2 (Remarks for Author):

The authors have fully taken into account the reviewer's suggestions. The manuscript is now suitable for publication in EMBO Molecular Medicine.

Response: We sincerely thank the reviewer for the positive evaluation of our revised manuscript. We are grateful that the reviewer considers our revisions satisfactory and finds the manuscript suitable for publication in EMBO Molecular Medicine. We appreciate the reviewer's constructive comments throughout the review process, which have helped us improve the manuscript.

10th Jul 2026

Dear Prof. Zhang,

We are pleased to inform you that your manuscript is accepted for publication and is now being sent to our publisher to be included in the next available issue of EMBO Molecular Medicine.

Your manuscript will be processed for publication by EMBO Press. It will be copy edited and you will receive page proofs prior to publication. Please note that you will be contacted by Springer Nature Author Services to complete licensing and payment information.

You may qualify for financial assistance for your publication charges - either via a Springer Nature fully open access agreement or an EMBO initiative. Check your eligibility: https://link.springer.com/journal/44321/how-to-publish-with-us

Should you be planning a Press Release on your article, please notify the production team via this form in order to coordinate publication and release dates: https://forms.cloud.microsoft/e/eH4A8JVP6g

If you have any questions, please do not hesitate to contact the Editorial Office. Thank you for your contribution to EMBO Molecular Medicine.

Yours sincerely,
Zeljko Durdevic

Zeljko Durdevic
Senior Editor
EMBO Molecular Medicine

>>> Please note that it is EMBO Molecular Medicine policy for the transcript of the editorial process (containing referee reports and your response letter) to be published as an online supplement to each paper. If you do NOT want this, you will need to inform the Editorial Office via email immediately. More information is available here: https://link.springer.com/partners/embo-press/editorial-policies#Peer%20review

EMBO Press Author Checklist

Corresponding Author Name: Yuxia Zhang	
Journal Submitted to: EMBO Molecular Medicine	
Manuscript Number: EMM-2026-23673	

USEFUL LINKS FOR COMPLETING THIS FORM
The EMBO Journal - Author Guidelines
EMBO Reports - Author Guidelines
Molecular Systems Biology - Author Guidelines
EMBO Molecular Medicine - Author Guidelines

Reporting Checklist for Life Science Articles (updated January
This checklist is adapted from Materials Design Analysis Reporting (MDAR) Checklist for Authors. MDAR establishes a minimum set of requirements in transparent reporting in the life sciences (see Statement of Task: 10.31222/osf.io/9sm4x). Please follow the journal's guidelines in preparing your
Please note that a copy of this checklist will be published alongside your article.

Abridged guidelines for figures
1. Data
The data shown in figures should satisfy the following conditions:
- the data were obtained and processed according to the field's best practice and are presented to reflect the results of the experiments in an accurate and unbiased manner.
- ideally, figure panels should include only measurements that are directly comparable to each other and obtained with the same assay.
- plots include clearly labeled error bars for independent experiments and sample sizes. Unless justified, error bars should not be shown for technical
- if n<5, the individual data points from each experiment should be plotted. Any statistical test employed should be justified.
- Source Data should be included to report the data underlying figures according to the guidelines set out in the authorship guidelines on Data

2. Captions
Each figure caption should contain the following information, for each panel where they are relevant:
- a specification of the experimental system investigated (eg cell line, species name).
- the assay(s) and method(s) used to carry out the reported observations and measurements.
- an explicit mention of the biological and chemical entity(ies) that are being measured.
- an explicit mention of the biological and chemical entity(ies) that are altered/varied/perturbed in a controlled manner.
- the exact sample size (n) for each experimental group/condition, given as a number, not a range;
- a description of the sample collection allowing the reader to understand whether the samples represent technical or biological replicates (including how many animals, litters, cultures, etc.).
- a statement of how many times the experiment shown was independently replicated in the laboratory.
- definitions of statistical methods and measures:
  - common tests, such as t-test (please specify whether paired vs. unpaired), simple χ2 tests, Wilcoxon and Mann-Whitney tests, can be unambiguously identified by name only, but more complex techniques should be described in the methods section;
  - are tests one-sided or two-sided?
  - are there adjustments for multiple comparisons?
  - exact statistical test results, e.g., P values = x but not P values < x;
  - definition of 'center values' as median or average;
  - definition of error bars as s.d. or s.e.m.

Please complete ALL of the questions below.
Select "Not Applicable" only when the requested information is not relevant for your study.

Materials

Newly Created Materials	Information included in the manuscript?	In which section is the information available? (Reagents and Tools Table, Materials and Methods, Figures, Data Availability Section)
New materials and reagents need to be available; do any restrictions apply?	Yes	Reagents and Tools Table

Antibodies	Information included in the manuscript?	In which section is the information available? (Reagents and Tools Table, Materials and Methods, Figures, Data Availability Section)
For **antibodies** provide the following information:<br>- Commercial antibodies: RRID (if possible) or supplier name, catalogue number and or/clone number<br>- Non-commercial: RRID or citation	Yes	Reagents and Tools Table

DNA and RNA sequences	Information included in the manuscript?	In which section is the information available? (Reagents and Tools Table, Materials and Methods, Figures, Data Availability Section)
**Short novel DNA or RNA including primers, probes**: provide the sequences.	Yes	Reagents and Tools Table

Cell materials	Information included in the manuscript?	In which section is the information available? (Reagents and Tools Table, Materials and Methods, Figures, Data Availability Section)
**Cell lines:** Provide species information, strain. Provide accession number in repository **OR** supplier name, catalog number, clone number, and/**OR** RRID.	Yes	Reagents and Tools Table
**Primary cultures:** Provide species, strain, sex of origin, genetic modification status.	Not Applicable	
Report if the cell lines were recently **authenticated** (e.g., by STR profiling) and tested for mycoplasma contamination.	Not Applicable	

Experimental animals	Information included in the manuscript?	In which section is the information available? (Reagents and Tools Table, Materials and Methods, Figures, Data Availability Section)
**Laboratory animals or Model organisms:** Provide species, strain, sex, age, genetic modification status. Provide accession number in repository **OR** supplier name, catalog number, clone number, **OR** RRID.	Yes	Materials and Methods
**Animal observed in or captured from the field:** Provide species, sex, and age where possible.	Not Applicable	
Please detail **housing and husbandry conditions**.	Yes	Materials and Methods

Plants and microbes	Information included in the manuscript?	In which section is the information available? (Reagents and Tools Table, Materials and Methods, Figures, Data Availability Section)
**Plants:** provide species and strain, ecotype and cultivar where relevant, unique accession number if available, and source (including location for collected wild specimens).	Not Applicable	
**Microbes:** provide species and strain, unique accession number if available, and source.	Not Applicable	

Human research participants	Information included in the manuscript?	In which section is the information available? (Reagents and Tools Table, Materials and Methods, Figures, Data Availability Section)
If collected and within the bounds of privacy constraints report on age, sex and gender or ethnicity for all study participants.	Yes	Supplemental table 1. Clinical characteristics

Core facilities	Information included in the manuscript?	In which section is the information available? (Reagents and Tools Table, Materials and Methods, Figures, Data Availability Section)
If your work benefited from core facilities, was their service mentioned in the acknowledgments section?	Not Applicable	

Design

Study protocol	Information included in the manuscript?	In which section is the information available? (Reagents and Tools Table, Materials and Methods, Figures, Data Availability Section)
If study protocol has been **pre-registered, provide DOI in the manuscript**. For clinical trials, provide the trial registration number **OR** cite DOI.	Yes	Materials and Methods
Report the **clinical trial registration number** (at ClinicalTrials.gov or equivalent), where applicable.	Not Applicable	

Laboratory protocol	Information included in the manuscript?	In which section is the information available? (Reagents and Tools Table, Materials and Methods, Figures, Data Availability Section)
Provide DOI OR other citation details if **external detailed step-by-step protocols** are available.	Yes	Materials and Methods

Experimental study design and statistics	Information included in the manuscript?	In which section is the information available? (Reagents and Tools Table, Materials and Methods, Figures, Data Availability Section)
Include a statement about **sample size** estimate even if no statistical methods were used.	Yes	Materials and Methods
Were any steps taken to minimize the effects of subjective bias when allocating animals/samples to treatment (e.g. **randomization procedure**)? If yes, have they been described?	Yes	Materials and Methods
Include a statement about **blinding** even if no blinding was done.	Yes	Materials and Methods
Describe **inclusion/exclusion criteria** if samples or animals were excluded from the analysis. Were the criteria pre-established?<br><br>If sample or data points were omitted from analysis, report if this was due to attrition or intentional exclusion and provide justification.	Not Applicable	
For every figure, are **statistical tests** justified as appropriate? Do the data meet the assumptions of the tests (e.g., normal distribution)? Describe any methods used to assess it. Is there an estimate of variation within each group of data? Is the variance similar between the groups that are being statistically compared?	Yes	Figure legends and Materials and Methods

Sample definition and in-laboratory replication	Information included in the manuscript?	In which section is the information available? (Reagents and Tools Table, Materials and Methods, Figures, Data Availability Section)
In the figure legends: state number of times the experiment was **replicated** in laboratory.	Yes	Figure legends
In the figure legends: define whether data describe **technical or biological replicates**.	Yes	Figure legends

Ethics

Ethics	Information included in the manuscript?	In which section is the information available? (Reagents and Tools Table, Materials and Methods, Figures, Data Availability Section)
Studies involving **human participants**: State details of **authority granting ethics approval** (IRB or equivalent committee(s), provide reference number for approval.	Yes	Materials and Methods
Studies involving **human participants**: Include a statement confirming that **informed consent** was obtained from all subjects and that the experiments conformed to the principles set out in the WMA Declaration of Helsinki and the Department of Health and Human Services Belmont Report.	Yes	Materials and Methods
Studies involving **human participants: For** publication of **patient photos**, include a statement confirming that consent to publish was obtained.	Not Applicable	
Studies involving experimental **animals**: State details of **authority granting ethics approval** (IRB or equivalent committee(s), provide reference number for approval. Include a statement of compliance with ethical regulations.	Yes	Materials and Methods
Studies involving **specimen and field samples:** State if relevant **permits** obtained, provide details of authority approving study; if none were required, explain why.	Not Applicable	

Dual Use Research of Concern (DURC)	Information included in the manuscript?	In which section is the information available? (Reagents and Tools Table, Materials and Methods, Figures, Data Availability Section)
Could your study fall under dual use research restrictions? Please check biosecurity documents and list of **select agents and toxins** (CDC): https://www.selectagents.gov/sat/list.htm	Not Applicable	
If you used a select agent, is the security level of the lab appropriate and reported in the manuscript?	Not Applicable	
If a study is subject to dual use research of concern regulations, is the name of the **authority granting approval and reference number** for the regulatory approval provided in the manuscript?	Not Applicable	

Reporting

The MDAR framework recommends adoption of discipline-specific guidelines, established and endorsed through community initiatives. Journals have their own policy about requiring specific guidelines and recommendations to complement MDAR.

Adherence to community standards	Information included in the manuscript?	In which section is the information available? (Reagents and Tools Table, Materials and Methods, Figures, Data Availability Section)
State if relevant guidelines or checklists (e.g., **ICMJE, MIBBI, ARRIVE, PRISMA**) have been followed or provided.	Yes	Materials and Methods
For **tumor marker prognostic studies,** we recommend that you follow the **REMARK** reporting guidelines (see link list at top right). See author guidelines, under 'Reporting Guidelines'. Please confirm you have followed these guidelines.	Not Applicable	
For **phase II and III randomized controlled trials**, please refer to the **CONSORT** flow diagram (see link list at top right) and submit the CONSORT checklist (see link list at top right) with your submission. See author guidelines, under 'Reporting Guidelines'. Please confirm you have submitted this list.	Not Applicable	

Data Availability

Data availability	Information included in the manuscript?	In which section is the information available? (Reagents and Tools Table, Materials and Methods, Figures, Data Availability Section)
Have **primary datasets** been deposited according to the journal's guidelines (see 'Data Deposition' section) and the respective accession numbers provided in the Data Availability Section?	Not Applicable	
Were **human clinical and genomic datasets** deposited in a public access-controlled repository in accordance to ethical obligations to the patients and to the applicable consent agreement?	Not Applicable	
Are **computational models** that are central and integral to a study available without restrictions in a machine-readable form? Were the relevant accession numbers or links provided?	Not Applicable	
If publicly available data were reused, provide the respective **data citations in the reference list**.	Not Applicable	
