# Supplementary material for: RADX protects against intestinal inflammation by restraining IFI16-mediated innate immunity
Source: EMBO Mol Med. 2026 Jul 24;18(9):3570–96. doi: 10.1038/s44321-026-00494-6 (PMC13562710; doi:10.1038/s44321-026-00494-6)
Supplement: Supplementary file 3 [file 44321_2026_494_MOESM1_ESM.docx]

**Table EV1. Clinical characteristics of IBD cohort**

| No. | Gender | Group | Age of onset | Diagnosis | Disease site | Treatment | Response to treatment | Family history |
| --- | --- | --- | --- | --- | --- | --- | --- | --- |
| 1 | Male | GIS (RADX) | 9 | CD | Colon | Infliximab, glucocorticoid and mercaptopurine | Effective | No |
| 2 | Male | GIS (RADX) | 4 | CD | Colon, perianal region | Infliximab, thalidomide, glucocorticoid and enterostomy | Effective | No |
| 3 | Male | GIS (ATRX) | 1 | CD | Colon | Infliximab, methotrexate, thalidomide, glucocorticoid and mercaptopurine | Effective | No |
| 4 | Male | GIS (POLA1/KNTC1) | 3 | IBD | Ileum | Infliximab and enterostomy | Not responding | No |
| 5 | Female | GIS (MCM6) | 3 | IBD | Colon, duodenum, ileum | Glucocorticoid and mesalamine | Effective | No |
| 6 | Female | GIS (MSH2) | 3 | IBD | Colon, duodenum, ileum | Glucocorticoid and mesalamine | Effective | No |
| 7 | Male | GIS (RPA4) | 3 | IBD | Colon, duodenum, ileum | Infliximab, glucocorticoid and enterostomy | Not responding | No |
| 8 | Male | Monogenic (COL7A1) | 8 | CD | Ileum | Infliximab and thalidomide | Effective | No |
| 9 | Male | Monogenic (TTC7A) | < 1 | IBD | Colon, duodenum | NA | NA | No |
| 10 | Female | WT | 9 | CD | Colon | Thalidomide, mesalamine, infliximab and EEN | Effective | No |
| 11 | Male | WT | < 2 | IBD | Colon | NA | NA | No |
| 12 | Male | Monogenic (XIAP) | 10 | IBD | Colon | Thalidomide, infliximab, and EEN | Effective | CD (Brother) |
| 13 | Female | WT | 12 | CD | Colon | Infliximab and EEN | Effective | No |
| 14 | Male | Monogenic (NPC1) | < 2 | IBD | Colon | Thalidomide and glucocorticoid | Effective | No |
| 15 | Female | WT | 13 | CD | Colon, perianal region, rectum | Infliximab, thalidomide, and EEN | Effective | No |
| 16 | Male | WT | 13 | CD | Colon, duodenum, ileum | Infliximab and EEN | Effective | CD (Sister) |
| 17 | Male | WT | NA | CD | NA | Infliximab and azathioprine | Effective | NA |
| 18 | Male | Monogenic (SLCO2A1) | 10 | IBD | Duodenum, ileum | Infliximab, EEN and mercaptopurine | Not responding | No |
| 19 | Male | WT | 8 | CD | Colon | Infliximab, mercaptopurine and thalidomide | Effective | Thalassemia (Mother), colorectal Cancer (Maternal grandma) |
| 20 | Female | WT | 1 | IBD | Colon | Thalidomide, infliximab, and glucocorticoid | Effective | No |
| 21 | Male | WT | 13 | CD | Colon, duodenum | Adalimumab, infliximab, and EEN | Effective | No |
| 22 | Male | WT | 4 | CD | Colon, duodenum | Infliximab | Effective | No |
| 23 | Male | WT | 2 | IBD | Colon | Thalidomide | Effective | No |
| 24 | Female | Monogenic (IL10RB) | 1 | IBD | Colon, perianal region | Enterostomy | NA | No |
| 25 | Male | WT | < 1 | IBD | Colon | Infliximab and glucocorticoid | Effective | Thalassemia carrier (Mother) |
| 26 | Male | Monogenic (FOXP3) | NA | IBD | NA | NA | NA | NA |
| 27 | Female | WT | NA | IBD | NA | NA | NA | NA |
| 28 | Male | WT | 11 | CD | Colon, perianal region, duodenum, ileum | Infliximab | Effective | No |
| 29 | Male | WT | 1 | IBD | Colon | NA | NA | No |
| 30 | Female | WT | 8 | CD | Colon, ileum | Infliximab | Effective | No |
| 31 | Male | WT | 2 | IBD | Colon | NA | NA | No |
| 32 | Male | WT | 7 | UC | Ileum | Infliximab and thalidomide | Effective | Rheumatoid arthritis (Grandma), arthritis (Father) |
| 33 | Male | WT | 2 | IBD | Colon | Infliximab, thalidomide, glucocorticoid and adalimumab | Effective | No |
| 34 | Male | WT | NA | CD | NA | NA | NA | NA |
| 35 | Male | Monogenic (CYBB) | < 1 | IBD | NA | NA | NA | No |
| 36 | Male | WT | 14 | CD | Colon, duodenum | Infliximab, mesalamine, mercaptopurine and glucocorticoid | Effective | No |
| 37 | Male | WT | 2 | IBD | NA | NA | NA | No |
| 38 | Male | WT | 4 | UC | Colon, ileum | Thalidomide, mesalamine and glucocorticoid | Effective | Thalassemia (Father) |
| 39 | Female | WT | < 2 | CD | Colon, duodenum, ileum | NA | NA | No |
| 40 | Male | WT | 10 | CD | Colon | Glucocorticoid and mercaptopurine | Effective | No |
| 41 | Female | WT | 12 | CD | Colon | Infliximab | Effective | Thalassemia (Father) |
| 42 | Female | WT | 10 | CD | Colon, ileum | Adalimumab, infliximab, mesalamine, and EEN | Effective | No |
| 43 | Male | WT | 14 | CD | Ileum | Mercaptopurine and infliximab | Effective | No |
| 44 | Male | WT | 12 | CD | Colon | Thalidomide, infliximab, mercaptopurine and glucocorticoid | Effective | No |
| 45 | Male | WT | 10 | CD | Ileum | Glucocorticoid and mercaptopurine | Effective | No |
| 46 | Male | WT | 13 | CD | Colon, perianal region, ileum | Infliximab, EN and mercaptopurine | Effective | No |
| 47 | Female | WT | 14 | CD | Colon, ileum | Infliximab and EEN | Effective | No |
| 48 | Male | Monogenic (XIAP) | 8 | CD | Colon, duodenum | Glucocorticoid and EN | NA | No |
| 49 | Male | WT | < 2 | IBD | Colon, duodenum | Glucocorticoid, infliximab and EN | NA | No |
| 50 | Male | WT | < 1 | IBD | Colon, ileum | EN | Effective | G6PD deficiency (Mother) |
| 51 | Female | WT | NA | IBD | NA | NA | NA | NA |
| 52 | Male | WT | < 1 | IBD | Colon, duodenum, ileum | Glucocorticoid |  | No |
| 53 | Male | WT | < 1 | CD | Colon, duodenum | Infliximab | Effective | No |
| 54 | Female | WT | 1 | CD | Colon, duodenum, rectum | Mesalamine and glucocorticoid | Effective | Gastritis (Mother) |
| 55 | Female | WT | 4 | UC | Colon | Mesalamine and glucocorticoid | NA | No |
| 56 | Female | WT | 6 | UC | Colon | Mesalamine and glucocorticoid | Not responding | NA |
| 57 | Female | WT | < 2 | UC | Colon | Infliximab and thalidomide | Effective | No |
| 58 | Female | WT | 11 | CD | Colon | Adalimumab, infliximab, and thalidomide | Effective | No |
| 59 | Female | WT | 13 | CD | Colon | Mercaptopurine and EEN | Effective | No |
| 60 | Male | WT | 5 | CD | Colon, duodenum | Infliximab | Effective | No |
| 61 | Male | WT | NA | CD | NA | NA | NA | NA |
| 62 | Female | Monogenic (LRBA) | 8 | CD | Colon, duodenum | Infliximab and EEN | Effective | No |
| 63 | Male | WT | NA | IBD | NA | NA | NA | NA |
| 64 | Male | Monogenic (FOXP3) | < 1 | IBD | Colon, duodenum | Glucocorticoid and mesalamine | Effective | No |
| 65 | Female | WT | < 2 | IBD | NA | Infliximab | Effective | NA |
| 66 | Male | WT | 9 | CD | Colon, ileum | Glucocorticoid, EEN, and mercaptopurine | Effective | No |
| 67 | Male | WT | 3 | CD | Colon | Glucocorticoid, infliximab, and mercaptopurine | Effective | No |
| 68 | Male | WT | 3 | CD | Colon | Infliximab, glucocorticoid and methotrexate | Effective | Thalassemia carrier (Father), hematochezia (Sister) |
| 69 | Female | WT | 2 | CD | Colon | Infliximab, glucocorticoid and mercaptopurine | Effective | No |
| 70 | Male | WT | < 1 | UC | Colon | EN and glucocorticoid | Not responding | NA |
| 71 | Male | WT | 6 | CD | Colon | Infliximab | Effective | No |
| 72 | Male | WT | 1 | CD | Colon | Glucocorticoid | Effective | G6PD deficiency (Brother) |
| 73 | Female | WT | 13 | CD | Colon, ileum | Mesalamine and glucocorticoid | Effective | NA |
| 74 | Female | WT | 2 | CD | Colon, ileum | Infliximab, mesalamine and glucocorticoid | Effective | No |
| 75 | Female | Monogenic (TTC37) | 1 | CD | Colon, ileum | Glucocorticoid and EN | Effective | NA |
| 76 | Female | WT | 1 | IBD | Colon, ileum | Enterostomy | NA | NA |
| 77 | Male | Monogenic (CYBB) | 2 | CD | Colon, fistula | Infliximab and mercaptopurine | Not responding | No |
| 78 | Male | WT | < 1 | IBD | Colon, perianal region | EEN, infliximab, and adalimumab | Effective | No |
| 79 | Female | WT | 8 | CD | Colon | Glucocorticoid, mercaptopurine, and thalidomide | Effective | No |
| 80 | Male | WT | 15 | IBD | Ileum, rectum | NA | NA | No |
| 81 | Male | WT | 1 | CD | Colon | NA | NA | No |
| 82 | Male | WT | 11 | CD | Colon | Ustekinumab | Effective | No |
| 83 | Female | WT | 12 | CD | Colon, rectum | Vedolizumab | Not responding | No |
| 84 | Male | WT | 17 | CD | Colon, ileum | Ustekinumab | Effective | No |
| 85 | Female | WT | 6 | CD | Colon, ileum | Ustekinumab | Effective | No |
| 86 | Female | WT | 11 | CD | Ileum | Adalimumab | Effective | No |
| 87 | Female | WT | 12 | CD | Colon, ileum, rectum | Glucocorticoid, thalidomide, and PEN | Not responding | No |
| 88 | Female | WT | 12 | CD | Colon | Adalimumab | Effective | No |
| 89 | Female | WT | 12 | CD | Colon, ileum, rectum | Ustekinumab | Not responding | No |
| 90 | Male | WT | 14 | CD | Colon, ileum, rectum | Ustekinumab and adalimumab | Effective | No |
| 91 | Female | WT | 15 | CD | Colon, ileum, rectum | Ustekinumab | Not responding | No |
| 92 | Male | WT | 16 | CD | Colon, rectum | Adalimumab | Effective | No |
| 93 | Female | WT | 16 | CD | Colon, ileum, rectum | Ustekinumab and methotrexate | Not responding | No |

NA: Not available. EN: Enteral nutrition. EEN: Exclusive enteral nutrition. PEN: Partial enteral nutrition.
